# Posterior wall ablation by pulsed-field ablation: procedural safety, efficacy, and findings on redo procedures

**DOI:** 10.1093/europace/euae006

**Published:** 2024-01-16

**Authors:** Thomas Kueffer, Hildegard Tanner, Antonio Madaffari, Jens Seiler, Andreas Haeberlin, Jens Maurhofer, Fabian Noti, Claudia Herrera, Gregor Thalmann, Nikola A Kozhuharov, Tobias Reichlin, Laurent Roten

**Affiliations:** Department of Cardiology, Inselspital, Bern University Hospital, University of Bern, Bern, Switzerland; ARTORG Center for Biomedical Engineering Research, University of Bern, Bern, Switzerland; Department of Cardiology, Inselspital, Bern University Hospital, University of Bern, Bern, Switzerland; Department of Cardiology, Inselspital, Bern University Hospital, University of Bern, Bern, Switzerland; Department of Cardiology, Inselspital, Bern University Hospital, University of Bern, Bern, Switzerland; Department of Cardiology, Inselspital, Bern University Hospital, University of Bern, Bern, Switzerland; ARTORG Center for Biomedical Engineering Research, University of Bern, Bern, Switzerland; Department of Cardiology, Inselspital, Bern University Hospital, University of Bern, Bern, Switzerland; Department of Cardiology, Inselspital, Bern University Hospital, University of Bern, Bern, Switzerland; Department of Cardiology, Inselspital, Bern University Hospital, University of Bern, Bern, Switzerland; Department of Cardiology, Inselspital, Bern University Hospital, University of Bern, Bern, Switzerland; Department of Cardiology, Inselspital, Bern University Hospital, University of Bern, Bern, Switzerland; Department of Cardiology, Inselspital, Bern University Hospital, University of Bern, Bern, Switzerland; Department of Cardiology, Inselspital, Bern University Hospital, University of Bern, Bern, Switzerland

**Keywords:** Atrial fibrillation, Pulsed-field ablation, Reconnection, Pulmonary vein isolation, Posterior wall ablation

## Abstract

**Aims:**

The left atrial posterior wall is a potential ablation target in patients with recurrent atrial fibrillation despite durable pulmonary vein isolation or in patients with roof-dependent atrial tachycardia (AT). Pulsed-field ablation (PFA) offers efficient and safe posterior wall ablation (PWA), but available data are scarce.

**Methods and results:**

Consecutive patients undergoing PWA using PFA were included. Posterior wall ablation was performed using a pentaspline PFA catheter and verified by 3D-electroanatomical mapping. Follow-up was performed using 7-day Holter ECGs 3, 6, and 12 months after ablation. Recurrence of any atrial arrhythmia lasting more than 30 s was defined as failure. Lesion durability was assessed during redo procedures. Posterior wall ablation was performed in 215 patients (70% males, median age 70 [IQR 61–75] years, 67% redo procedures) and was successful in all patients (100%) by applying a median of 36 (IQR 32–44) PFA lesions. Severe adverse events were cardiac tamponade and vascular access complication in one patient each (0.9%). Median follow-up was 7.3 (IQR 5.0–11.8) months. One-year arrhythmia-free outcome in Kaplan–Meier analysis was 53%. A redo procedure was performed in 26 patients (12%) after a median of 6.9 (IQR 2.4–11) months and showed durable PWA in 22 patients (85%) with only minor lesion regression. Among four patients with posterior wall reconnection, three (75%) presented with roof-dependent AT.

**Conclusion:**

Posterior wall ablation with this pentaspline PFA catheter can be safely and efficiently performed with a high durability observed during redo procedures. The added value of durable PWA for the treatment of atrial fibrillation remains to be evaluated.

## Graphical Abstract

**Graphical Abstract:**
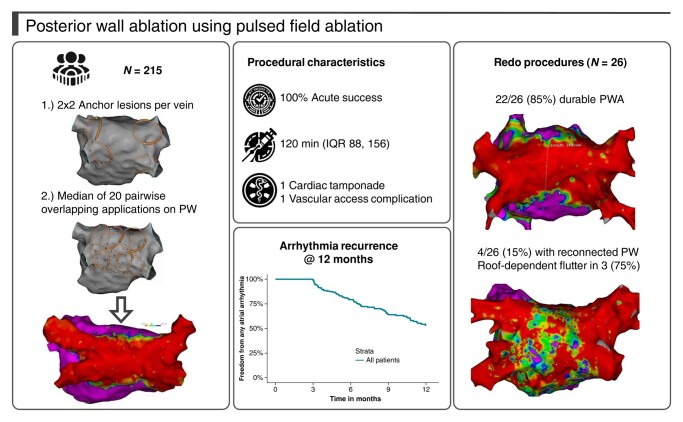

**What’s new?**

- Pulsed-field ablation of the left atrial posterior wall with a pentaspline catheter effectively and efficiently isolates the posterior wall in all cases and has an excellent safety profile.
- Repeat procedures confirm high lesion durability and only minor lesion regression.
- Roof-dependent atrial tachycardia are common in posterior wall reconnection, emphasizing durable ablation's importance.

## Introduction

Pulmonary vein isolation (PVI) is the treatment of choice in the interventional management of atrial fibrillation (AF).^1^ Despite successful, durable PVI, AF recurs in 10–15% of patients with paroxysmal AF and even more frequently in patients with persistent AF.^2^ In these patients, ablation of additional sites harbouring AF triggers or perpetuators is necessary. The left atrial (LA) posterior wall (PW) is one of the main targets beyond PVI, as it shares a common embryologic origin with the pulmonary veins and hence is thought to be arrhythmogenic.^3,4^ The posterior wall may also have to be ablated in patients with a roof-dependent LA tachycardia.^5^

So far, randomized controlled trials adding posterior wall isolation (PWI) to PVI in patients with persistent AF yielded conflicting results. While some smaller studies showed a benefit,^6–8^ both the POBI-AF study nor the recent CAPLA trial did.^9,10^ Importantly, all these studies used thermal ablation technologies for PWI that has significant limitations: (1) failure to complete PWI in a significant number of cases; (2) reconnection of PWI during follow-up; and (3) limitation of energy delivery on the posterior wall due to safety concerns, in particular damage to the adjacent oesophagus. Hence, the true value of PWI for treating AF has yet to be established using efficient and safe tools for complete and durable PWI.

Pulsed-field ablation (PFA) is a non-thermal ablation technology that specifically targets the cardiac muscle fibres and leaves surrounding structures unharmed. It applies microsecond-scale pulses that disrupt cell membranes, leading to cell death. The first PFA catheter gained CE approval in January 2021. This PFA catheter was designed for PVI and can change its configuration from a basket to a flower configuration. In its flower configuration, the catheter is well-suited for posterior wall ablation (PWA) and may overcome the limitations of PWI by thermal ablation.

## Methods

### Study population

All consecutive patients with paroxysmal AF, persistent AF, or LA tachycardia undergoing an LA ablation procedure including PWA using the FARAPULSE PFA system (Boston Scientific, USA) at the Bern University Hospital until the end of the year 2022 were analysed. All patients were enrolled in a prospective institutional ablation registry. The registry was approved by the local ethics committee and carried out in accordance with the Declaration of Helsinki. The authors had full access to the data and bear full responsibility for its accuracy.

### Ablation procedure

Posterior wall ablation was performed using the PFA platform consisting of a generator, a long steerable sheath, and an ablation catheter (FARASTAR, FARADRIVE, and FARAWAVE, Boston Scientific, Menlo Park, CU, USA). The generator of the platform delivers high-intensity, bipolar, and biphasic electric pulses to the catheter electrodes, creating an electric field that disrupts the membranes of adjacent cells and leads to irreversible electroporation and cell death.^11^

Prior to the procedure, patients underwent transoesophageal echocardiography and computed tomography to exclude intracardiac thrombi and to obtain a detailed understanding of the left atrial anatomy. Deep conscious sedation using propofol and fentanyl was used, guided by a physician-led, nurse-administered protocol, while patients with a high risk of sedation complications underwent general anaesthesia.^12^ Left atrial access was obtained by fluoroscopy-guided transseptal puncture either using a standard SL1 sheath, followed by an exchange to the 13F FARADRIVE sheath, or through a direct puncture using the 13F sheath, depending on the physician’s preference.^13^

**Figure 1:**
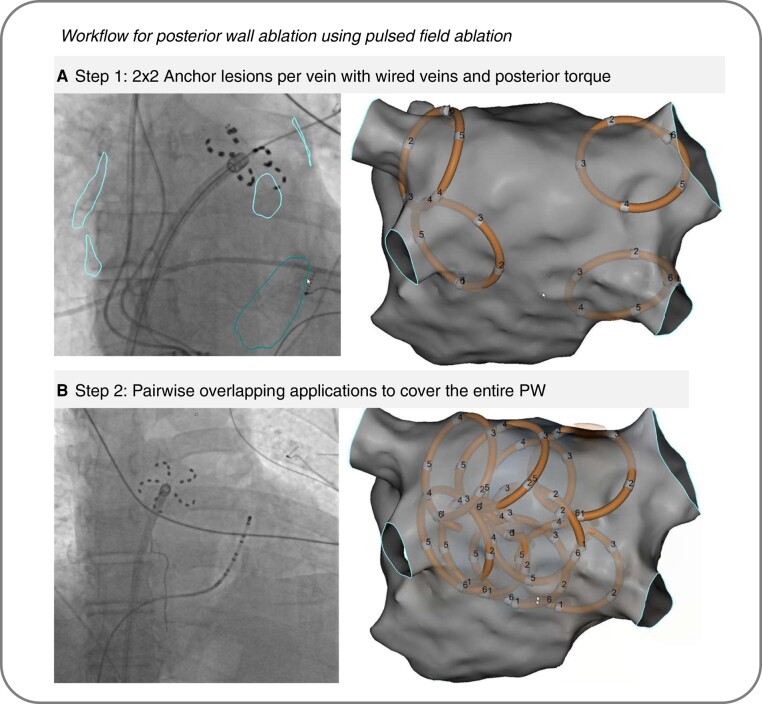
Workflow for posterior wall ablation using pulsed-field ablation. After pulmonary vein isolation, four additional anchor lesions per vein in flower configuration and with posterior torque on the FARADRIVE sheath were placed to extend the lesion further into the posterior wall (*A*). Successive ablation of the posterior wall was achieved by targeting the entire space between the left and right pulmonary veins with pairwise, overlapping applications and without rotation of the device. PW, posterior wall.

In the majority of patients, a 3D-electroanatomical mapping (3D-EAM) system (CARTO 3, Biosense Webser, Irvine, CA, USA) and a high-density multi-electrode mapping catheter (PENTARAY or OCTARAY, Biosense Webser, Irvine, CA, USA) were used before PWA to identify previously isolated PVs, as applicable, and to characterize left atrial substrate. Before PWA, PVI was performed as necessary with the PFA system and according to standard practice.^14^ Posterior wall ablation was performed in two steps: first, posterior anchor lesions were applied to each vein with the device in flower configuration and the guidewire placed in a vein, applying strong posterior torque to the sheath (*Figure 1A*). For each vein, four anchor lesions were placed, and the catheter rotated by 30° after the first two applications. Second, the guidewire was retracted and pairwise, overlapping lesions were placed without catheter rotation between applications, covering the entire posterior wall from the upper end of the superior veins to the lower end of the inferior veins (*Figure 1B*). Voltage amplitude for PFA was changed from 1.9 kV to 2.0 kV following the recommendation of the manufacturer in September 2021. Catheter size selection (31 vs. 35 mm) was at the discretion of the operator.

Following PWA, a detailed 3D-EAM was again generated with the multi-spline catheter and additional PFA lesions were applied as necessary to complete PWA. Depending on the individual case, additional ablation of a posterior or anterior mitral isthmus line, LA substrate ablation, ablation of the cavotricuspid isthmus, isolation of the superior vena cava, and right atrial substrate ablation were performed using either PFA or radiofrequency ablation.

### Follow-up

Patients were followed with 7-day Holter ECGs 3, 6, and 12 months after the ablation procedure. Recurrence was defined as any atrial tachyarrhythmia (AF, atrial flutter, or AT) lasting longer than 30 s between Days 91 and 365 post-ablation of the posterior wall. In case of a symptomatic recurrence, further options were discussed with the patient and a redo procedure offered.

### Mapping protocol at repeat ablation

All repeat ablation procedures were again performed using a 3D-EAM system and a high-density multi-electrode mapping catheter. 3D-EAM was used to identify the tachycardia mechanism, lesion durability, and regression, and to identify LA substrate. Pre-ablation maps of the redo procedure were compared to post-ablation maps of the previous procedure.

Based on the high-density 3D maps, the target lesion set for the repeat procedure was determined and additional ablation was performed with either point-by-point radiofrequency ablation or PFA, according to the operator’s preference.

### Statistical analysis

Continuous data are shown as mean (± standard deviation) or median (interquartile range) as appropriate and were compared with the Mann–Whitney *U* test, the *t*-test, or the Kruskal–Wallis test, as appropriate. Categorical variables were reported as counts (percentage) and compared with a Pearson χ^2^ test or the Fisher exact test, as appropriate. Kaplan–Meier analyses with log-rank test were performed for arrhythmia recurrence. The statistical analysis was conducted using R 4.2.2 (R Core Team, Vienna, Austria). A two-sided *P*-value of ≤0.05 was considered significant.

## Results

### Patient population and ablation history

From May 2021 to December 2022, 215 patients underwent an LA ablation procedure including PWA using PFA at our institution. Of these, 64 (30%) were female, median age was 69 (IQR 61–75) years and median CHADS-VASC score 2 (IQR 1–4). Posterior wall ablation was targeted during a first procedure in 70 (33%) patients and in 145 (66%) during a redo procedure. The arrhythmia that led to the first left atrial ablation procedure was persistent AF in 151 (70%), paroxysmal AF in 56 (26%), and left AT in 8 (4%). Patients coming for a redo procedure had a median of 1 previous procedures (IQR 1–2). Baseline patient characteristic and details about previous ablation targets can be found in *Table 1*.

**Table 1:** Baseline patient characteristics

Variable	Overall
*n*	215
Age—years	69.5 [61.3, 75.3]
Sex, male	151 (70.2)
Body mass index—kg/m^2^	27.7 [24.6, 32.5]
Primary AF type—no. (%)	
Persistent	151 (70.2)
Paroxysmal	56 (26.0)
AT	8 (3.7)
Duration of persistent AF—months	26.0 [6.0, 67.5]
CHA_2_DS_2_-VASc score	2.0 [1.0, 4.0]
Redo LA ablation procedure—no. (%)	145 (67.4)
Ablation technology of the previous intervention—no. (%)	
RFA	87 (60.0)
CBA	30 (20.7)
PFA	22 (15.2)
Surgical	6 (4.1)
Previous DCCV—no. (%)	107 (49.8)
Hypertension—no. (%)	133 (61.9)
Previous heart failure—no (%)	51 (23.7)
Diabetes—no. (%)	28 (13.0)
Previous stroke or TIA—no. (%)	16 (7.4)
Chronic obstructive pulmonary disease—no. (%)	15 (7.0)
Sleep apnoea—no. (%)	36 (16.7)
Class III AAD—no. (%)	70 (32.6)
Left atrial volume index—mL/m^2^	46.5 [37.0, 55.2]
Left ventricular ejection fraction—%	55.0 [45.0, 60.0]
Left common ostium—no. (%)	40 (18.6)

Numbers are median [IQR] unless otherwise noted.

AAD, antiarrhythmic drug; AF, atrial fibrillation; AT, atrial tachycardia; CBA, cryoballoon ablation; DCCV, direct-current cardioversion; IQR, interquartile range; LA, left atrial; PFA, pulsed-field ablation; RFA, radiofrequency ablation; TIA, transient ischaemic attack.

### Ablation procedure including posterior wall ablation

**Figure 2:**
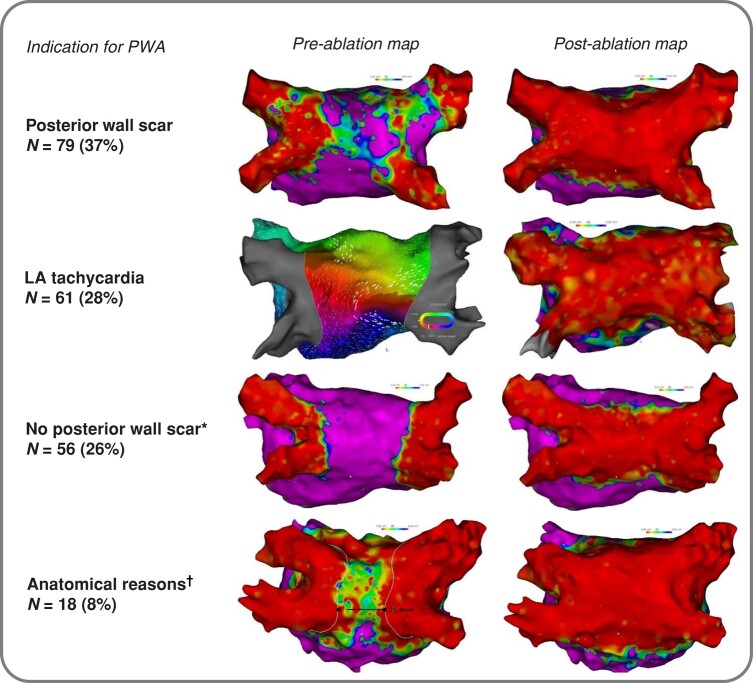
Indications for posterior wall ablation. Indication to perform posterior wall ablation was either: *A*) presence of posterior wall scar, identified by 3D-EAM; *B*) left atrial tachycardia; *C*) no posterior wall scar (asterisk): PWA was performed either due to recurrence despite durable PVI or as a first-line treatment strategy based on the patient’s medical history; and *D*) anatomical reasons (dagger): PWA was performed either due to a narrow surviving channel after PVI or due to an anomalous roof vein. 3D-EAM, 3D-electroanatomical mapping; LA, left atrial; PWA, posterior wall ablation.

Indication to perform PWA was the presence of PW scar in 79 patients (37%), no PW scar with either recurrence despite durable PVI or as a first-line treatment decision based on patient history in 56 (26%), LA tachycardia in 62 (29%), and anatomical reasons (narrow, remaining gap of tissue on the posterior wall after PVI using PFA; or anomalous accessory roof or posterior pulmonary vein) in 18 (8%) (*Figure 2*). The 31 mm sized PFA device was used in 200 (93%) of patients. A high-density map was generated prior to PWA in 191 (89%) of patients. In the remaining cases, PWA was performed solely using fluoroscopy for catheter guidance. In all patients, 16 anchor lesions were applied (4 per vein) plus a median number of 20 (IQR 16–28) PFA lesions to complete PWA. High-density maps showed successful PWA in 215/215 (100%) patients. No radiofrequency ablation was necessary to complete PWA in any patient. Additional ablation targets besides PWA in the same procedure were left to the discretion of the operator and included a posterior mitral isthmus line in 14 patients (7%), anterior mitral isthmus line in 28 (13%), LA substrate ablation in 20 (9%), ablation of the cava-tricuspid isthmus in 37 (17%), and isolation of the superior vena cava in 7 (3%). The supplementary table describes indications for PWA and additional ablation targets in patients with paroxysmal or persistent AF or left atrial tachycardia as the index arrhythmia for the first left atrial ablation procedure. Pericardial effusion requiring pericardiocentesis occurred in one patient and one patient had a small arteriovenous fistula at the puncture site, which was successfully managed by targeted compression. In the 43 patients undergoing a first left atrial ablation procedure including PVI and PWA, median procedure duration was 104 (IQR 79–128) min and median fluoroscopy time 24 (17–30) min. Procedural characteristics and lesion sets can be found in *Table 2* and *Figure 2*.

**Table 2:** Procedural data, complications, and follow-up

Indication for PWA	Overall	PW scar	LA flutter	No PW scar	Anatomy	*P*
*n*	215	79	62	56	18	
Procedure						
Redo procedure—no. (%)	145 (67.4)	55 (69.6)	44 (71.0)	37 (66.1)	9 (50.0)	0.382
Pre-map (3D-EAM)—no. (%)	192 (89.3)	79 (100.0)	61 (98.4)	36 (64.3)	16 (88.9)	<0.001
31 mm device	200 (93.0)	70 (88.6)	58 (93.5)	55 (98.2)	17 (94.4)	0.189
Number of PFA applications for anchor lesions	16.0 [16.0, 16.0]	16.0 [16.0, 16.0]	16.0 [16.0, 16.0]	16.0 [16.0, 16.0]	16.0 [16.0, 16.0]	–
Number of PFA applications for PWA	20.0 [16.0, 28.0]	20.0 [18.0, 28.0]	20.0 [16.0, 22.0]	24.0 [20.0, 32.0]	16.0 [12.5, 19.0]	<0.001
Total number of PFA applications for PWA	36.0 [32.0, 44.0]	36.0 [34.0, 44.0]	36.0 [32.0, 38.0]	40.0 [36.0, 48.0]	32.0 [28.5, 35.0]	<0.001
Successful PWA with PFA alone—no. (%)	1.0 [1.0, 1.0]	1.0 [1.0, 1.0]	1.0 [1.0, 1.0]	1.0 [1.0, 1.0]	1.0 [1.0, 1.0]	–
Posterior MIL—no. (%)	14 (6.5)	1 (1.3)	12 (19.4)	1 (1.8)	0	<0.001
Anterior MIL—no. (%)	28 (13.0)	6 (7.6)	21 (33.9)	0	1 (5.6)	<0.001
LA substrate ablation—no. (%)	20 (9.3)	4 (5.1)	13 (21.0)	3 (5.4)	0	0.002
CTI ablation using RFA—no. (%)	37 (17.2)	13 (16.5)	16 (25.8)	7 (12.5)	1 (5.6)	0.120
SVC isolation—no. (%)	7 (3.3)	3 (3.8)	3 (4.8)	1 (1.8)	0	0.669
Procedure time—min	120.0 [88.0, 156.0]	117.0 [88.0, 138.5]	162.5 [132.5, 204.2]	95.0 [76.8, 123.2]	97.5 [78.2, 110.8]	<0.001
Fluoroscopy time—min	21.0 [15.0, 29.1]	20.0 [14.9, 28.6]	24.6 [17.2, 36.0]	19.2 [13.2, 24.1]	23.7 [18.9, 26.5]	0.011
Fluoroscopy dose—Gycm^2^	6.4 [3.1, 11.8]	6.5 [2.9, 13.0]	6.7 [3.3, 13.1]	6.3 [3.4, 10.4]	5.6 [3.0, 7.6]	0.808
Complications	1.0 [1.0, 1.0]	1.0 [1.0, 1.0]	1.0 [1.0, 1.0]	1.0 [1.0, 1.0]	1.0 [1.0, 1.0]	–
Cardiac tamponade—no. (%)	1 (0.5)	1 (1.3)	0	0	0	0.630
Vascular complication—no. (%)	1 (0.5)	1 (1.3)	0	0	0	0.630
Cerebral complication—no. (%)	0	0	0	0	0	–
Oesophageal complication—no. (%)	0	0	0	0	0	–
Phrenic paresis—no. (%)	0	0	0	0	0	–
Follow-up	1.0 [1.0, 1.0]	1.0 [1.0, 1.0]	1.0 [1.0, 1.0]	1.0 [1.0, 1.0]	1.0 [1.0, 1.0]	–
Follow-up—months	7.3 [5.0, 11.8]	7.7 [5.0, 11.7]	5.9 [3.4, 11.9]	7.9 [6.0, 11.8]	9.4 [5.9, 11.5]	0.267
Recurrence of atrial arrhythmia—no. (%)	78 (36.8)	33 (42.9)	24 (39.3)	18 (32.1)	3 (16.7)	0.169
Time to recurrence—months	5.8 [3.7, 8.8]	6.2 [3.7, 7.9]	4.8 [3.2, 6.6]	7.7 [6.3, 10.8]	5.4 [4.2, 7.9]	0.014
Type of recurrence—no. (%)						0.001
AT	34 (45.9)	13 (39.4)	19 (82.6)	1 (6.7)	1 (33.3)	
Paroxysmal AF	17 (23.0)	7 (21.2)	3 (13.0)	6 (40.0)	1 (33.3)	
Persistent AF	23 (31.1)	13 (39.4)	1 (4.3)	8 (53.3)	1 (33.3)	
LA redo procedure—no. (%)	26 (12.1)	10 (12.7)	13 (21.0)	3 (5.4)	0	0.024
Time to redo procedure—months	6.9 [2.4, 11.0]	8.0 [1.2, 10.8]	6.4 [4.6, 11.9]	6.9 [0.0, 7.1]	0	0.193

Numbers are median [IQR] unless otherwise noted.

3D-EAM, 3D-electroanatomical mapping; AF, atrial fibrillation; AT, atrial tachycardia; CTI, cavotricuspid isthmus; IQR, interquartile range; MIL, mitral isthmus line; PFA, pulsed-field ablation; PWA, posterior wall ablation; RFA, radiofrequency ablation; SVC, superior vena cava.

### Follow-up and recurrence

**Figure 3:**
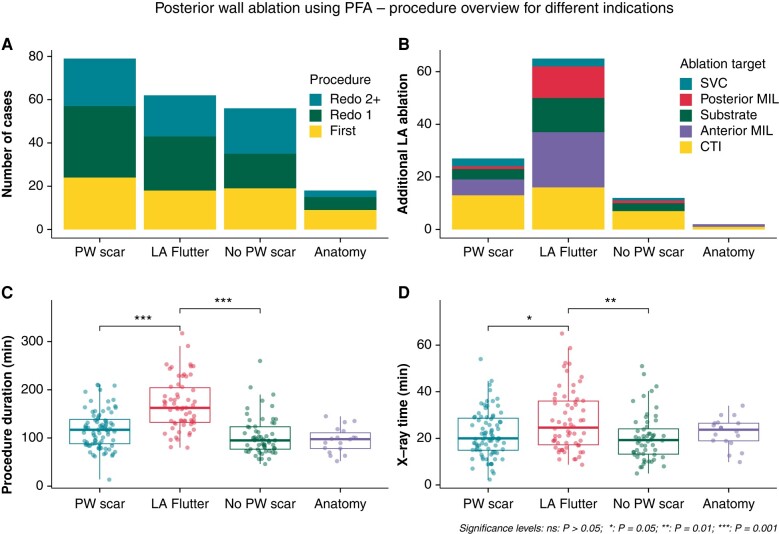
Procedure overview. Overview and procedural details for posterior wall ablation using PFA in 215 patients. *A*) Number of cases for each indication. *B*) Extra-pulmonary vein targets additional to PWA for each indication. Substrate ablation denotes the elimination of additional atrial tachycardia originating from a scar area that is not amenable to linear ablation. *C* and *D*) Differences in procedure duration and X-ray time. CTI, cavotricuspid isthmus; LA, left atrium; MIL, mitral isthmus line; PFA, pulsed-field ablation; PWA, posterior wall ablation; SVC, superior vena cava.

**Figure 4:**
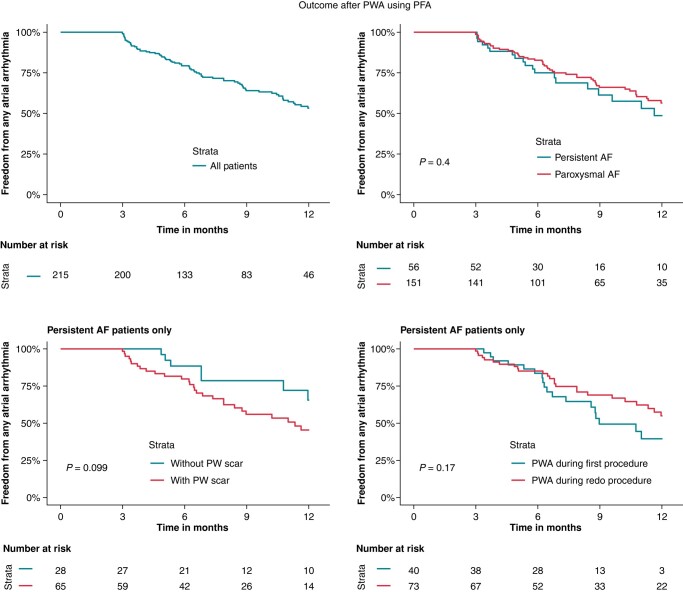
Kaplan-Meier analysis. Outcome after posterior wall ablation using pulsed-field ablation in 215 patients. No difference in arrhythmia-free outcome was found for persistent AF vs. paroxysmal AF patients, for patients with vs. without posterior wall scar, and for performing PWA during the first vs. during a redo procedure. AF, atrial fibrillation; PWA, posterior wall ablation.

The median follow-up time after PWA was 7.3 (IQR 5.0–11.8) months. An arrhythmia recurred in 78 patients (37%), a median of 5.8 (IQR 3.7–8.8) months after PWA. Arrhythmia recurrence was AT in 34 patients (46%), persistent AF in 23 (31%), and paroxysmal AF in 17 (23%) (*Figure 3*). Kaplan-Meier analysis showed survival free from any atrial arrhythmia of 79% after 6 months and 53% after 12 months in the complete cohort. There was no difference in survival free from arrhythmia between patients with persistent or paroxysmal AF (56% vs. 49%, *P* = 0.4) after one year. In patients treated for persistent AF, the presence of PW scar did not influence survival free from arrhythmia (45% vs. 65%, *P* = 0.099). Similarly, PWA during the first vs. PWA during a redo procedure did not affect survival free from arrhythmia in patients treated for persistent AF (40% vs. 55%, *P* = 0.17) (*Table 2*, *Figure 4*).

### Findings on redo procedures

A total of 26/68 (38%) patients with arrhythmia recurrence underwent a LA redo ablation procedure, 5 (19%) for AF and 21 (81%) for AT. Tachycardia mechanism for the latter was roof-dependent flutter in 3 (12%) cases, perimitral flutter in 4 (15%) cases, multiple LA AT’s in 3 (12%) cases, antral, scar-dependent re-entry in 3 (12%) cases, CTI, biatrial flutter, and an unidentified AT in 1 case each, and no AT could be induced in the remaining 5 cases. Details on redo ablation can be found in *Table 3*. High-density 3D-EAM identified 22/26 (85%) of the posterior walls with durable isolation, i.e. no reconnection across the posterior wall. In cases without reconnection the minimal width of the ablated area measured from the inferior to the superior scar border was reduced from 44 mm (IQR 40–50) to 41 mm (IQR 36–46). A large lesion regression of >10 mm was found in 4 (15%) cases (see Supplementary material online). In these, the scar border distance regressed from 50 mm (IQR 47–53) to 29 mm (IQR 26–30). Importantly, these 4 cases still showed conduction block across the posterior wall, preventing PW-dependent AT. In 4/26 cases (15%), the PW was no longer isolated, allowing for conduction across it. In 3 of these cases (75%), PW-dependent AT was identified and the remaining patient presented with perimitral AT. No procedural adverse events occurred during the redo procedures. Maps acquired during redo procedures and corresponding post-ablation maps from the index procedure can be found in the Supplementary material online.

**Table 3:** Redo procedure

Variable	Overall	Durable PWA	Non-durable PWA	*P*
*n*	26	22	4	
Time to recurrence—months	6.0 [3.8, 8.9]	6.0 [4.4, 8.9]	6.3 [3.5, 8.9]	0.696
Type of arrhythmia—no. (%)				0.570
AT	21 (80.8)	17 (77.3)	4 (100.0)	
Paroxysmal AF	1 (3.8)	1 (4.5)	0	
Persistent AF	4 (15.4)	4 (18.2)	0	
Time to redo procedure—months	8.8 [6.4, 11.8]	8.0 [6.4, 11.5]	10.7 [8.0, 11.9]	0.749
Lesion regression—mm	2.6 [0.1, 7.2]	2.6 [0.1, 7.2]	–	–

Numbers are median [IQR] unless otherwise noted.

AF, atrial fibrillation; AT, atrial tachycardia; IQR, interquartile range; PWA, posterior wall ablation.

## Discussion

In this large study of 215 consecutive patients undergoing PWA using a median of 36 PFA applications, our main findings are:

1. PWA using PFA was safe, efficient, and effective.
2. Remapping in patients with arrhythmia recurrence showed durable posterior wall isolation in 85% with only minor lesion regression.
3. The overall arrhythmia recurrence rate after 12 months was 47%.
4. A roof-dependent atrial tachycardia occurred in 75% of patients with posterior wall reconnection.

### Acute results

Our study shows that by using this pentaspline PFA device, we can achieve fast and effective, acute, complete posterior wall ablation in all patients. Similarly, the pre-market PersAFOne trial reported successful PWA in 25/25 (100%) patients.^15^ In comparison, acute posterior wall isolation by radiofrequency ablation was achieved in only 86.5% of patients in the recently published CAPLA trial, and half of the patients required additional ablation within the posterior wall to complete PWI.^10^ Pak *et al*. reported successful PWI in 94% of patients with radiofrequency ablation and used a protocol that mandated voltage map-guided ablation of any remnant atrial potentials on the posterior wall as well as an electrical exit block as an endpoint. In the later POBI-AF trial by the same group and using the same protocol and endpoints, complete PWI was also reported in all patients.^9^ With cryoballoon ablation of the posterior wall, Ahn *et al*.^6^ described successful PWI in 62% of the patients only. In another trial comparing PVI with PVI plus PWI, Aryana *et al*.^7^ reported successful PWI in all 55 patients using cryoballoon ablation, but touch-up radiofrequency ablation was necessary in almost half of the patients.

The pentaspline PFA catheter used in our study, in its flower pose, is ideally suited for PWA and highly effective without the need for touch-up thermal ablation. Importantly, it does require a large number of overlapping applications to completely cover the posterior wall and good tissue contact has to be ensured to achieve durable lesions. To this end, the integration and visualization of the PFA catheter into a 3D-EAM is very helpful, although PWA can also be achieved by fluoroscopy guidance alone. In our workflow, we always start PWA by applying 4 ‘anchor’ lesions on the posterior aspect of each pulmonary vein. If combined with PVI and to shorten procedure duration, these ‘anchor’ lesions can be applied after the standard 4 + 4 PFA applications per vein, with the guidewire still within the pulmonary vein. After completion of the ‘anchor’ lesions and retraction of the guidewire, two to three lines (depending on left atrial size) of PFA lesions are applied to cover the tissue in-between the left- and right-sided ‘anchor’ lesions.

### Procedural characteristics

With this technique, median procedure duration for PVI plus PWA in patients undergoing a first left atrial ablation procedure was 104 (IQR 49–128) min. In comparison, median procedure duration in the CAPLA trial and the POBI-AF trial for PVI plus PWA was 142 min and 227 min, respectively.^9,10^ With cryoballoon ablation, PVI plus PWA required a mean of 168 min in the study by Aryana *et al*.^7^ Therefore, PWA with PFA offers clearly improved procedural efficiency compared to thermal ablation technologies both in terms of acute success rate as well as procedure duration.

Adverse events were low in our study and well within rates reported by others.^16^ While thermal complications like oesophageal damage or phrenic nerve palsy are less probable with PFA, mechanical complications like pericardial effusions and vascular injuries can still occur. This is also true for air embolism or left atrial clot formation.^17^ However, a considerably larger number of cases will have to be treated with PFA, in particular including PWA, to give the final ‘all-clear’ regarding oesophageal damage caused by PFA. In the meantime, our study provides reassuring results by including the largest number of cases to date.

### Recurrence and durability

Arrhythmia recurrence after one year was still high in our population, and in the range reported by the CAPLA trial.^10^ However, we included a case mix of patients, many of whom had already failed several previous ablation procedures and most of them had persistent AF and advanced atrial cardiomyopathy. Hence, we cannot draw any firm conclusions regarding the value of PWA to avoid arrhythmia recurrence and randomized controlled trials with more strict inclusion criteria that evaluate the benefit of PVI plus PWA by PFA as compared to PVI alone will be needed.

For PWA to be successful, lesion durability is a key factor. In the PersAFOne study, all patients underwent a redo procedure after PWA by PFA and no reconnection was reported.^15^ Without mandated redo procedures, rates of durably isolated pulmonary veins as well as durably isolated posterior walls are generally underestimated because of a selection bias. This is because only patients with arrhythmia recurrence and among those only patients with sufficient arrhythmia burden will undergo a redo ablation procedure. In our study, 12% of patients had a redo procedure that is similar to the 10% in both the CAPLA trial and the POBI-AF trial.^9,10^ Durability of PW isolation was found in 31.2% of patients in CAPLA, and in 50% in POBI-AF. After PWA using PFA, we observed durable PWA in 85% of patients, and with only minor lesion regression. Although such indirect comparisons need to be taken with caution, it is remarkable that such low reconnection rates can be achieved with the first clinically available PFA catheter, which was neither optimized for PWA nor offers intrinsic 3D mapping integration. The latter will likely further ameliorate lesion precision and durability if feedback on tissue contact can be provided, as sufficient tissue contact is probably a key factor for durable lesions. Nevertheless, lesion durability is paramount for PWA by PFA, given that 75% of the patients with a reconnected posterior wall had roof-dependent AT in our study.

### Limitations

The following limitations of the present study merit consideration. First, this is a retrospective observational single-centre study. In particular, we did not have a specific ablation strategy. The decision to perform PWA and to add other ablation targets was at the discretion of the operator, as was the endpoint of the ablation procedure. Our results must be interpreted in conjunction with similar data reported from other groups. Secondly, only 26/68 (38%) patients with recurrence of an arrhythmia underwent repeat ablation, which might have introduced a selection bias. Thirdly, median follow-up time after PWA was only 7 months and longer-term follow-up data is needed to confirm our results. Finally, due to the low rate of reconnected posterior walls, a meaningful analysis regarding predictors of posterior wall reconnection was not possible in our study and larger data sets are necessary.

## Conclusion

Posterior wall ablation with this pentaspline PFA catheter is safe, effective, and efficient both with regard to the completeness of PWA and procedure duration. Posterior wall ablation is durable with only minor lesion regression in the majority of patients with arrhythmia recurrence. Most patients with posterior wall reconnection present with PW-dependent AT. The added value of durable PWA for the treatment of AF remains to be evaluated.

## Supplementary Material

euae006_Supplementary_Data

## Data Availability

The data that support the findings of this study are available from the corresponding author upon reasonable request.
