# Supplementary material for: Posterior wall ablation by pulsed-field ablation: procedural safety, efficacy, and findings on redo procedures
Source: Europace. 2024 Jan 16;26(1):euae006. doi: 10.1093/europace/euae006 (PMC10803044; doi:10.1093/europace/euae006)
Supplement: euae006_Supplementary_Data [file euae006_supplementary_data.docx]

Supplemental Material

**Posterior wall ablation by pulsed-field ablation – procedural safety, efficacy and findings on redo procedures**

Thomas Kueffer MSc1,2, Hildegard Tanner MD1, Antonio Madaffari MD1, Jens Seiler MD1, Andreas Haeberlin MD1,2, Jens Maurhofer MD1, Fabian Noti MD1, Gregor Thalmann MD1, Nikola A. Kozhuharov MD1, Tobias Reichlin MD1, Laurent Roten MD1

Page 2 – 10: Side to side comparison for 26 patients undergoing PWA by PFA. Pre-PWA maps are shown on the left, post-PWA maps in the middle and maps acquired at the start of the redo procedure on the right.

Page 11: Supplementary Table. Procedural characteristics and lesion set stratified by initial presenting arrhythmia

| Before posterior wall ablation | After posterior wall ablation | During redo procedure |
| --- | --- | --- |
| 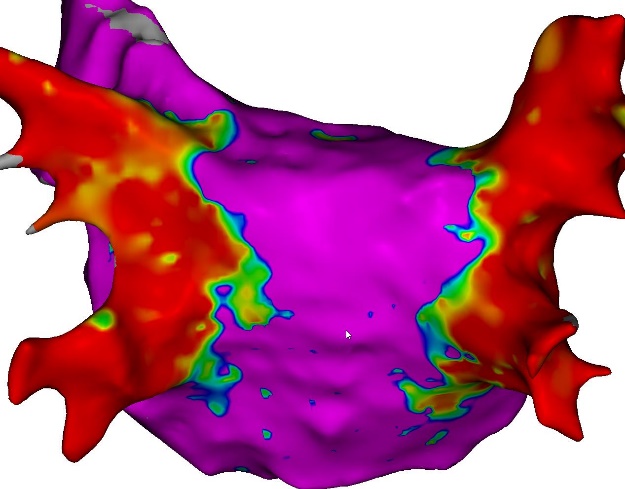 | 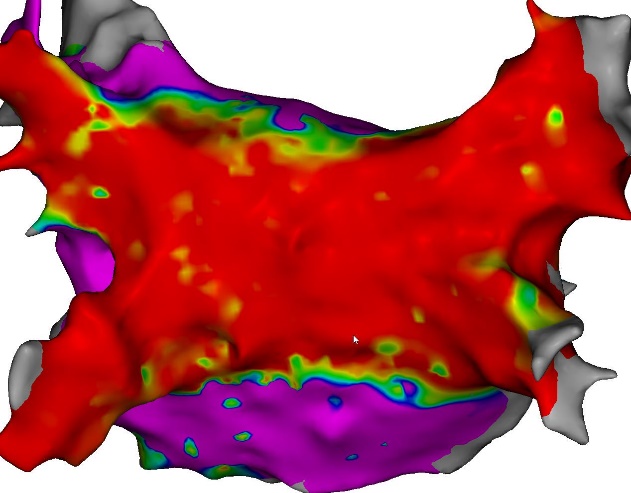 | 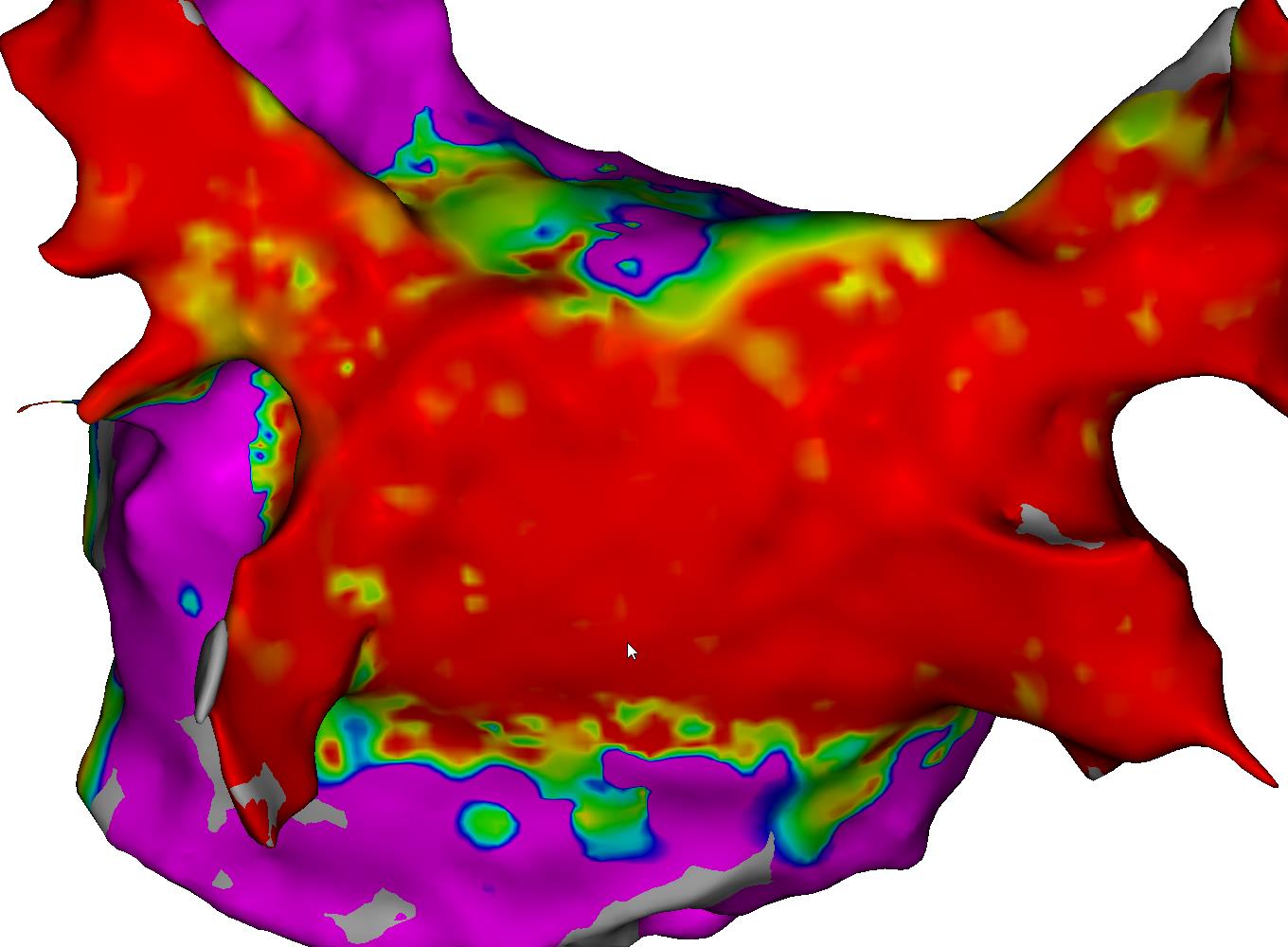 |
| 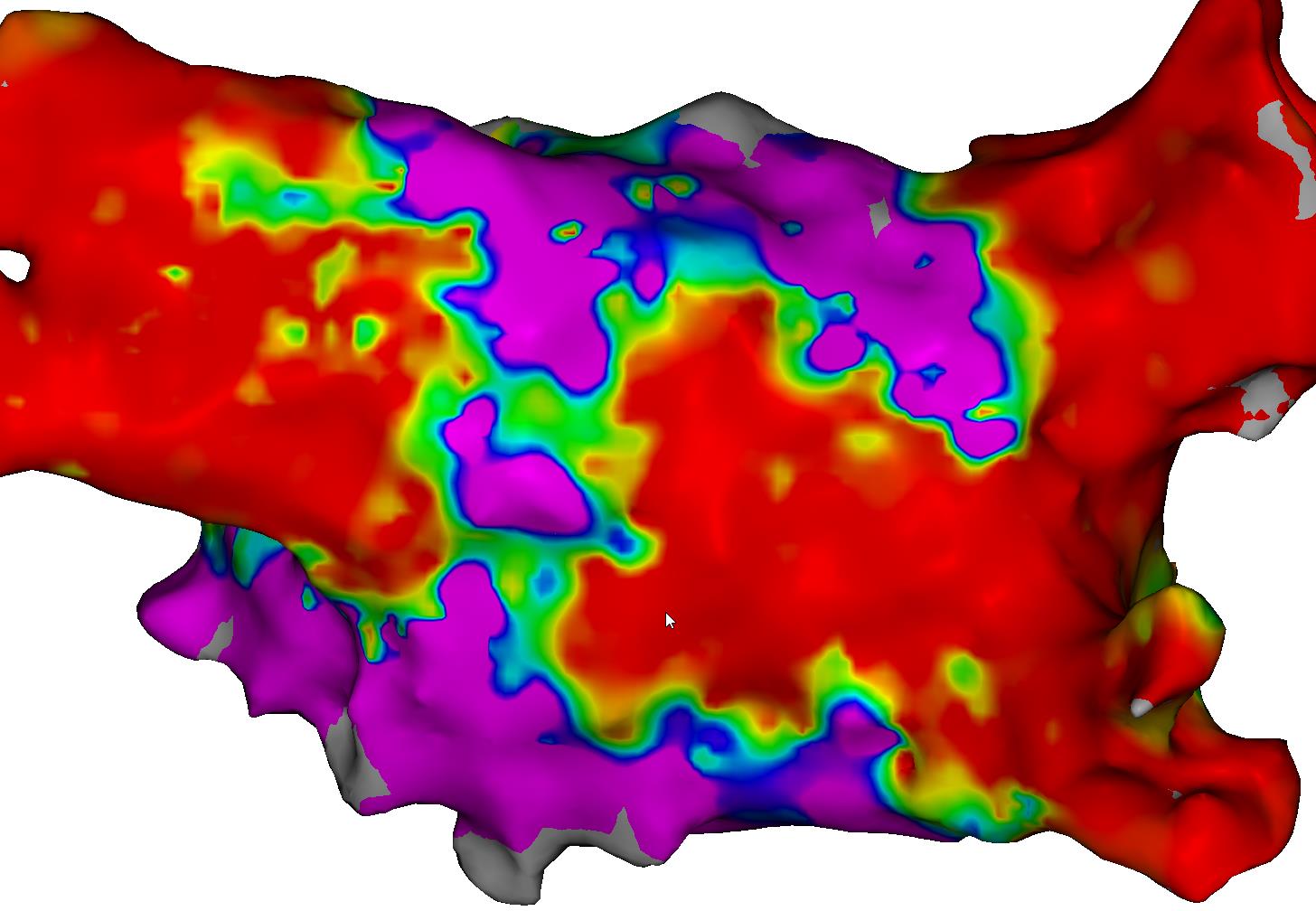 | 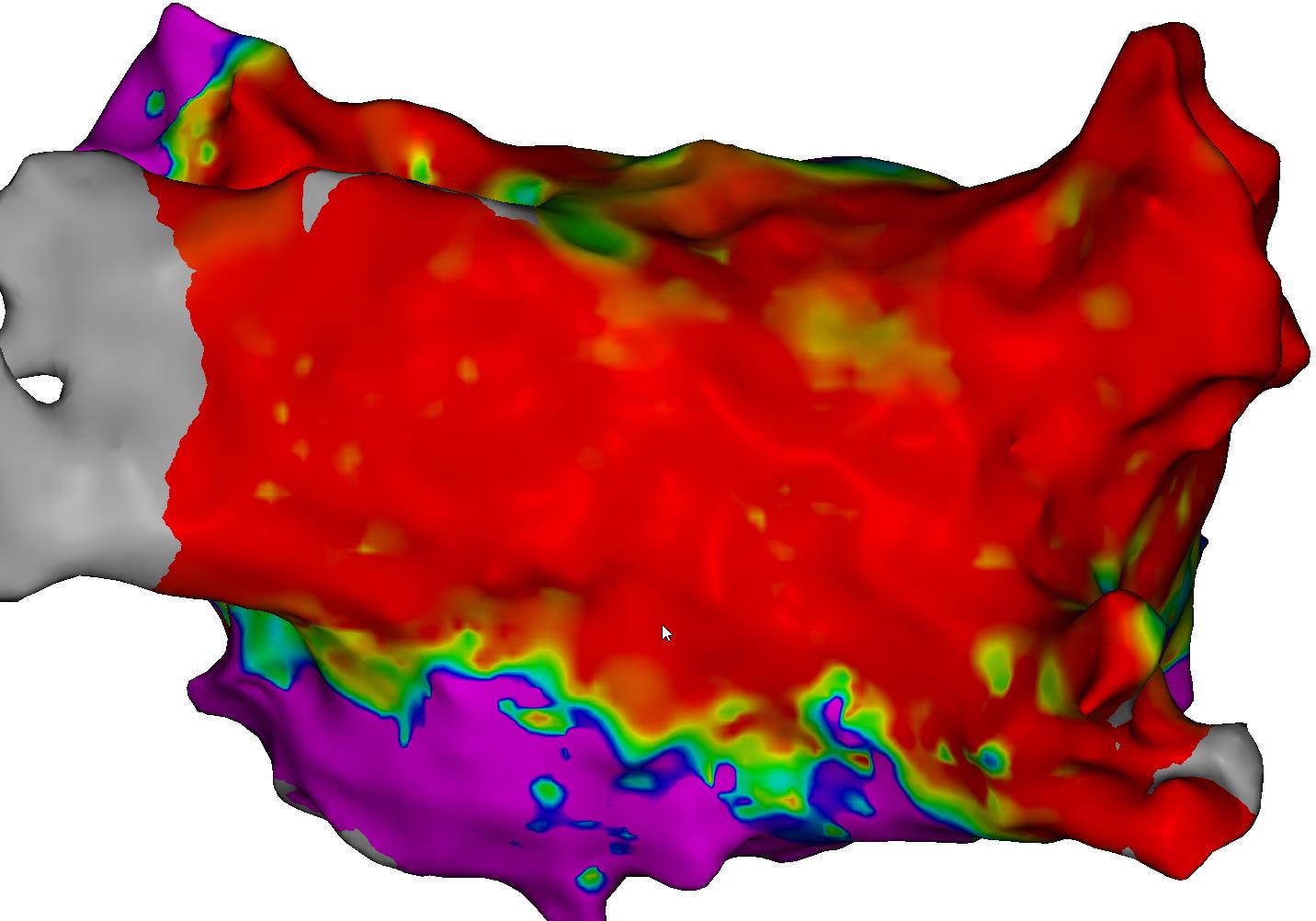 | 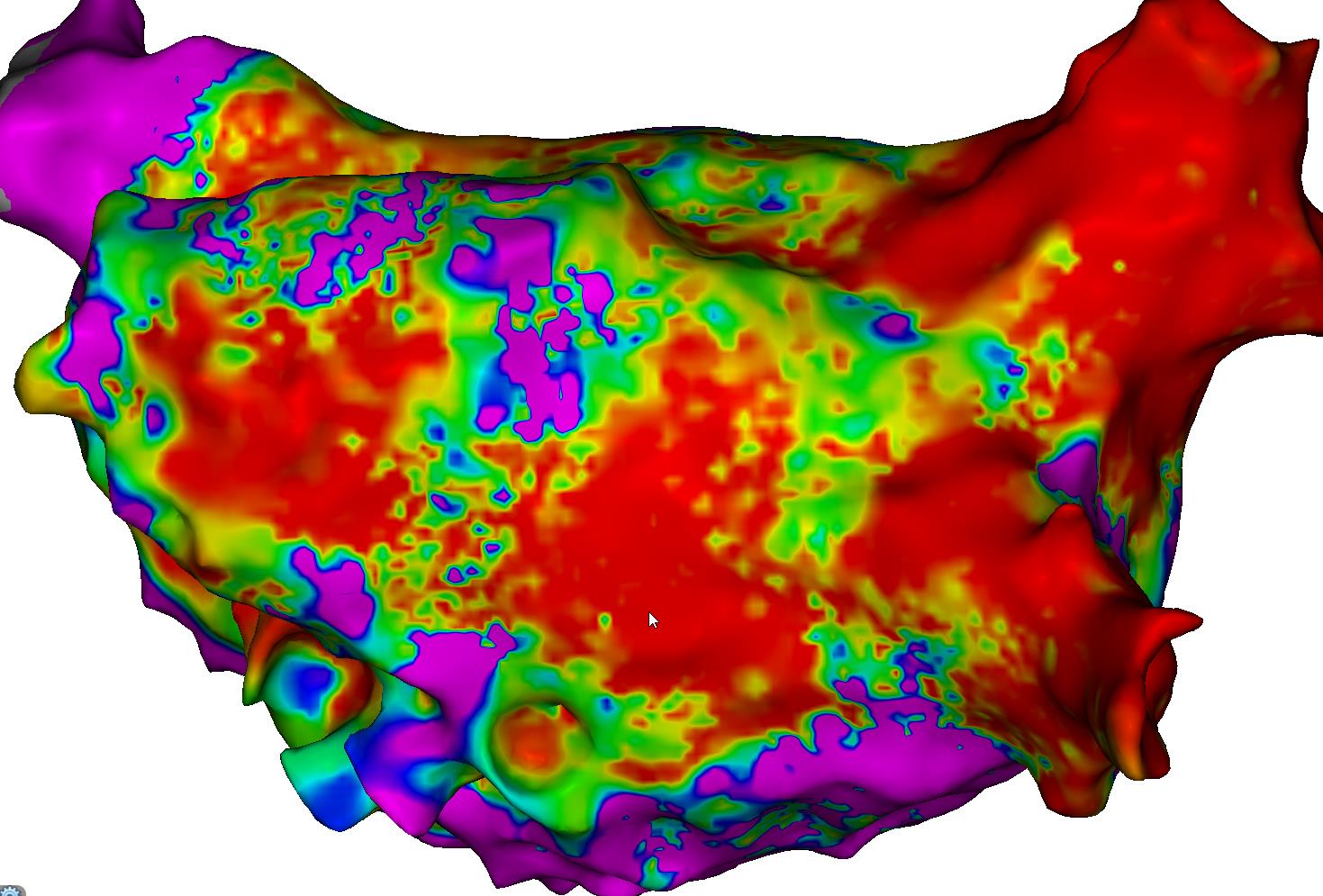 Reconnection, PW-dependent AT |
| 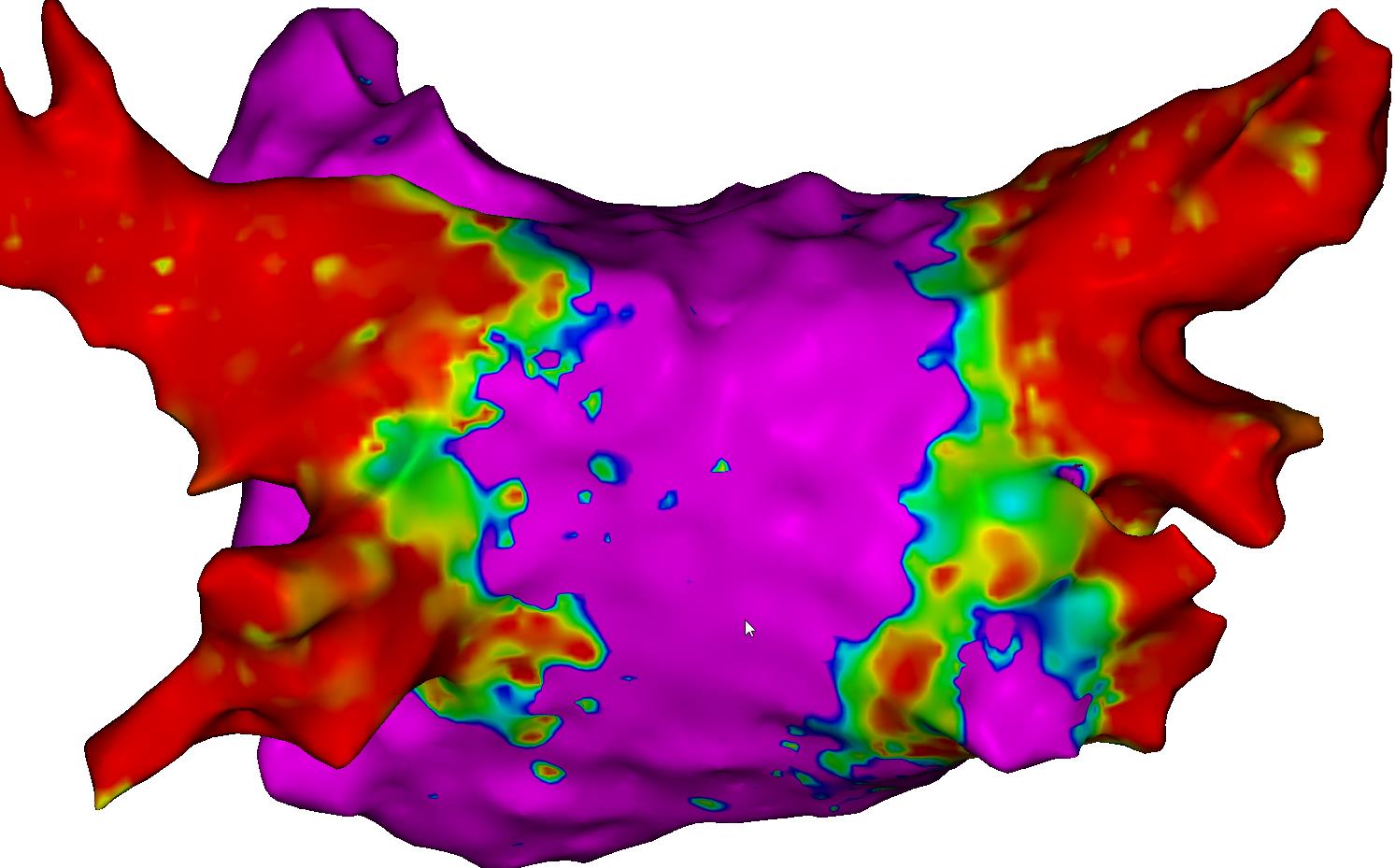 | 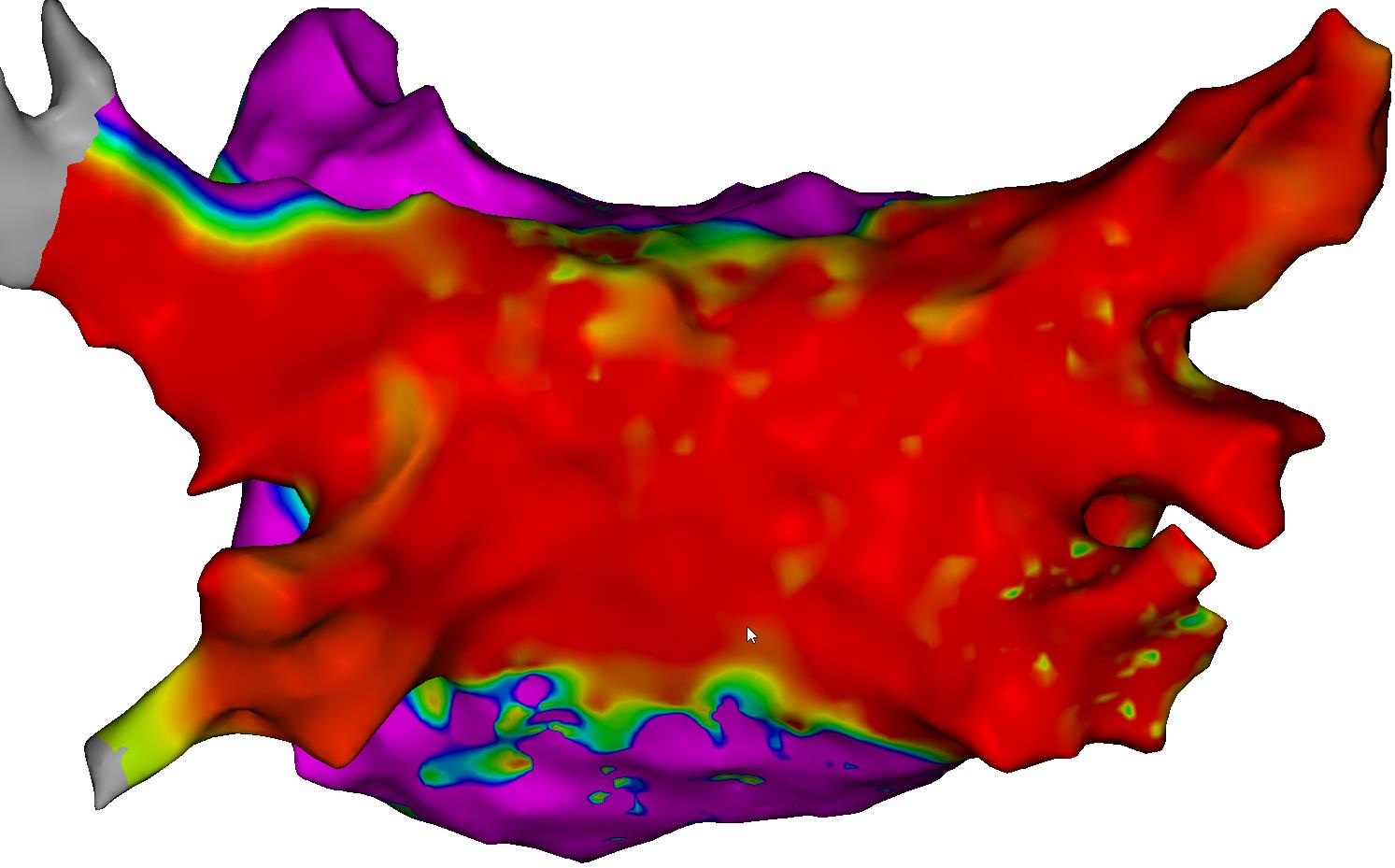 | 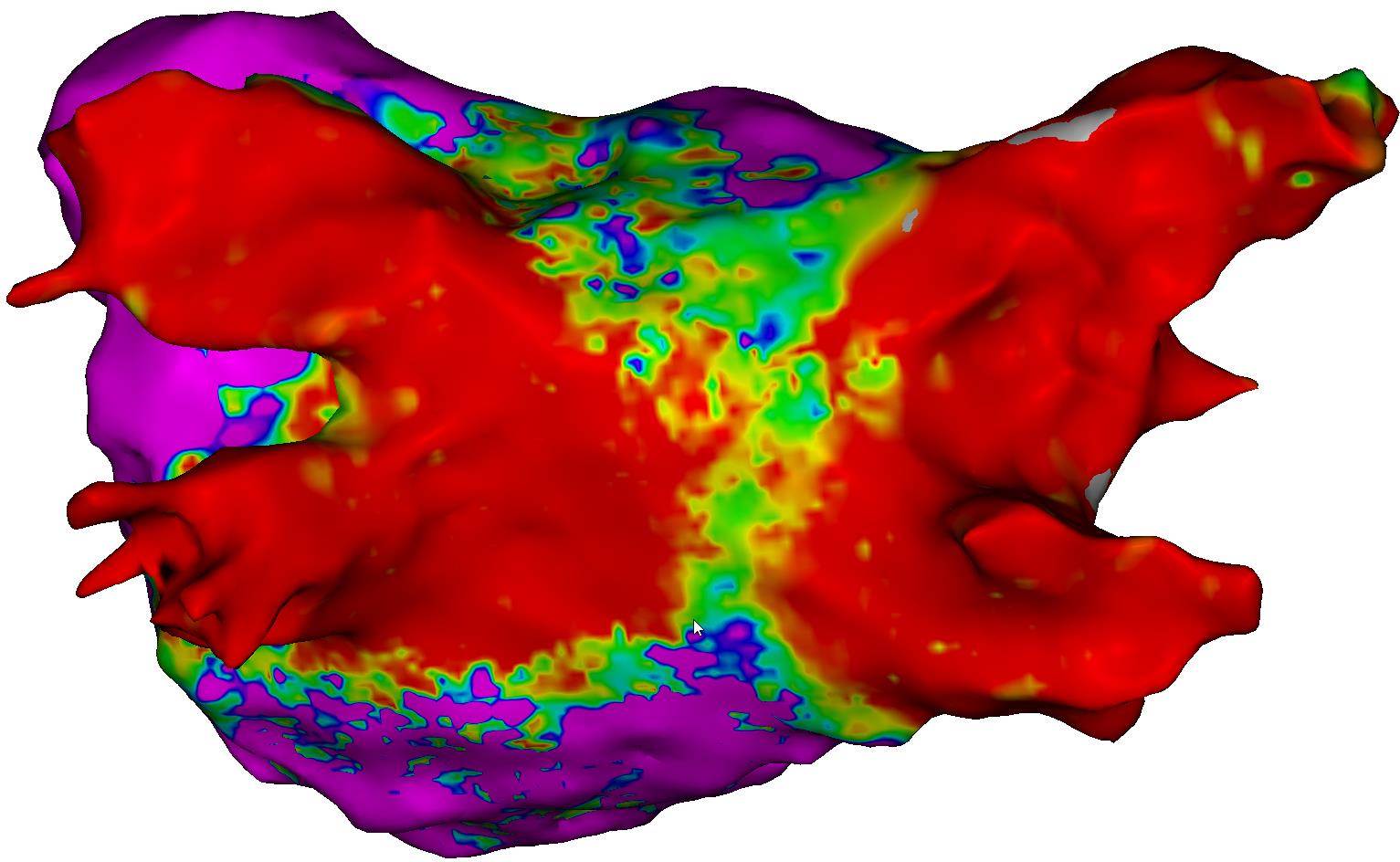 Reconnection, perimitral AT |
| 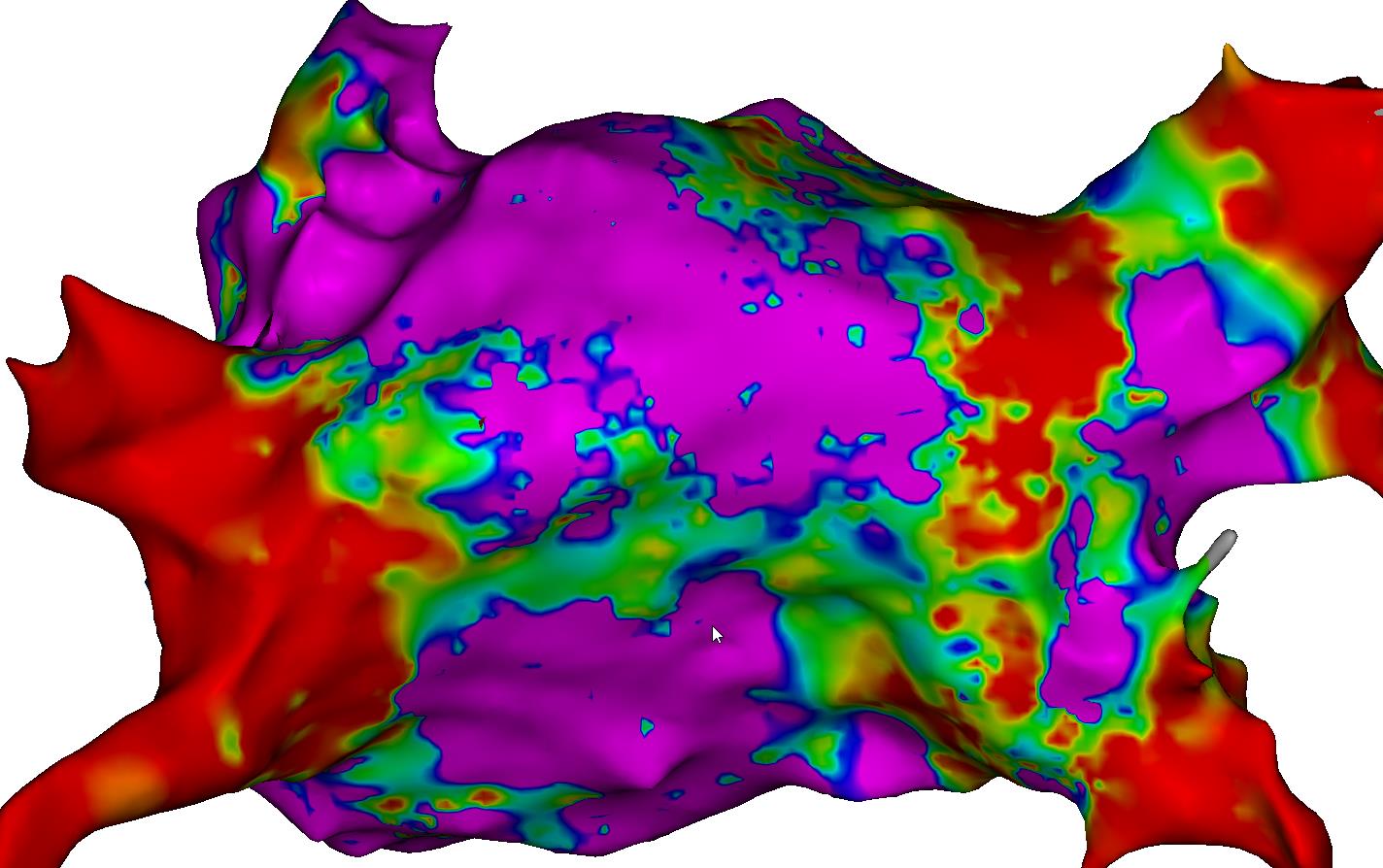 | 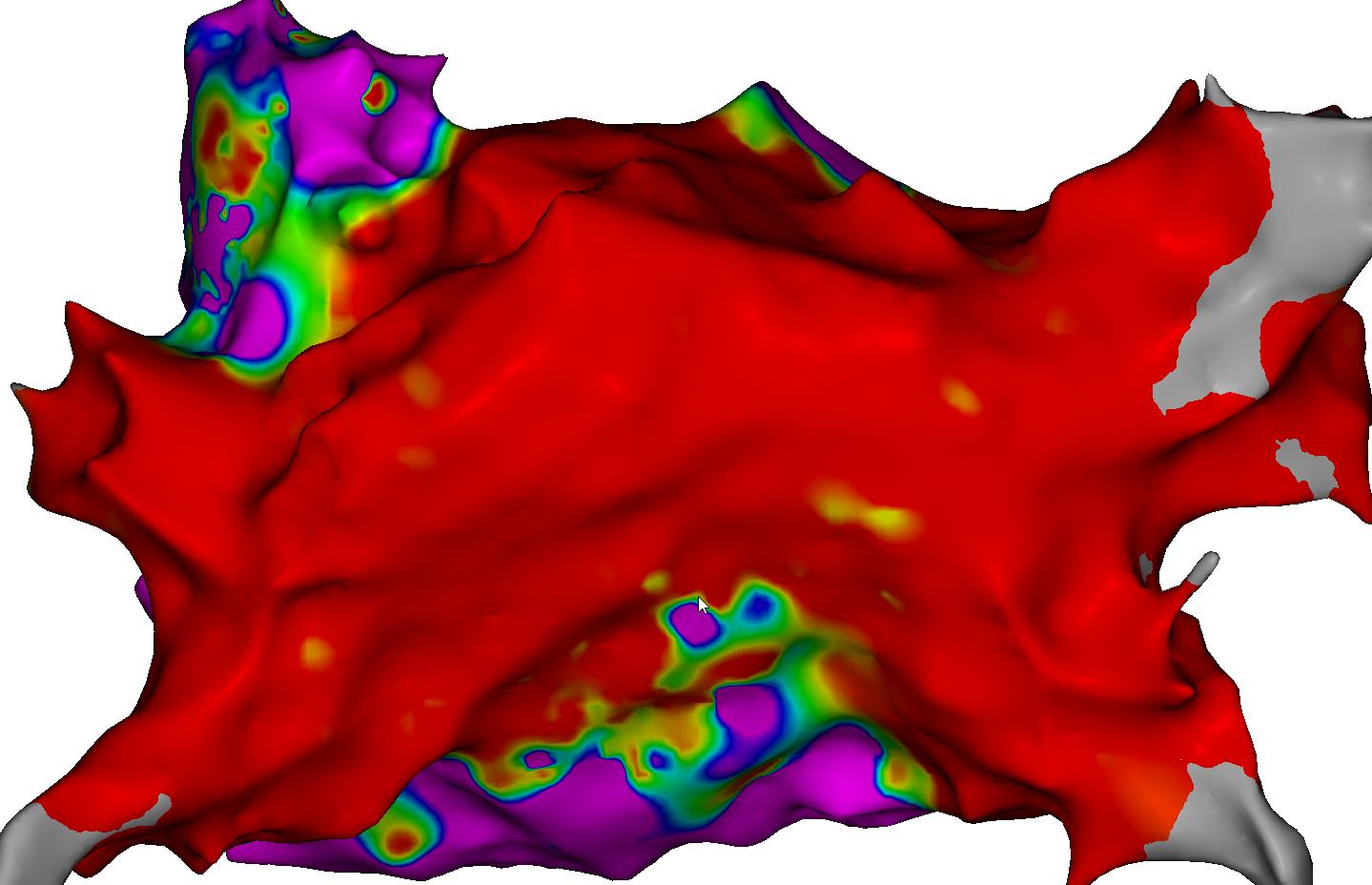 | 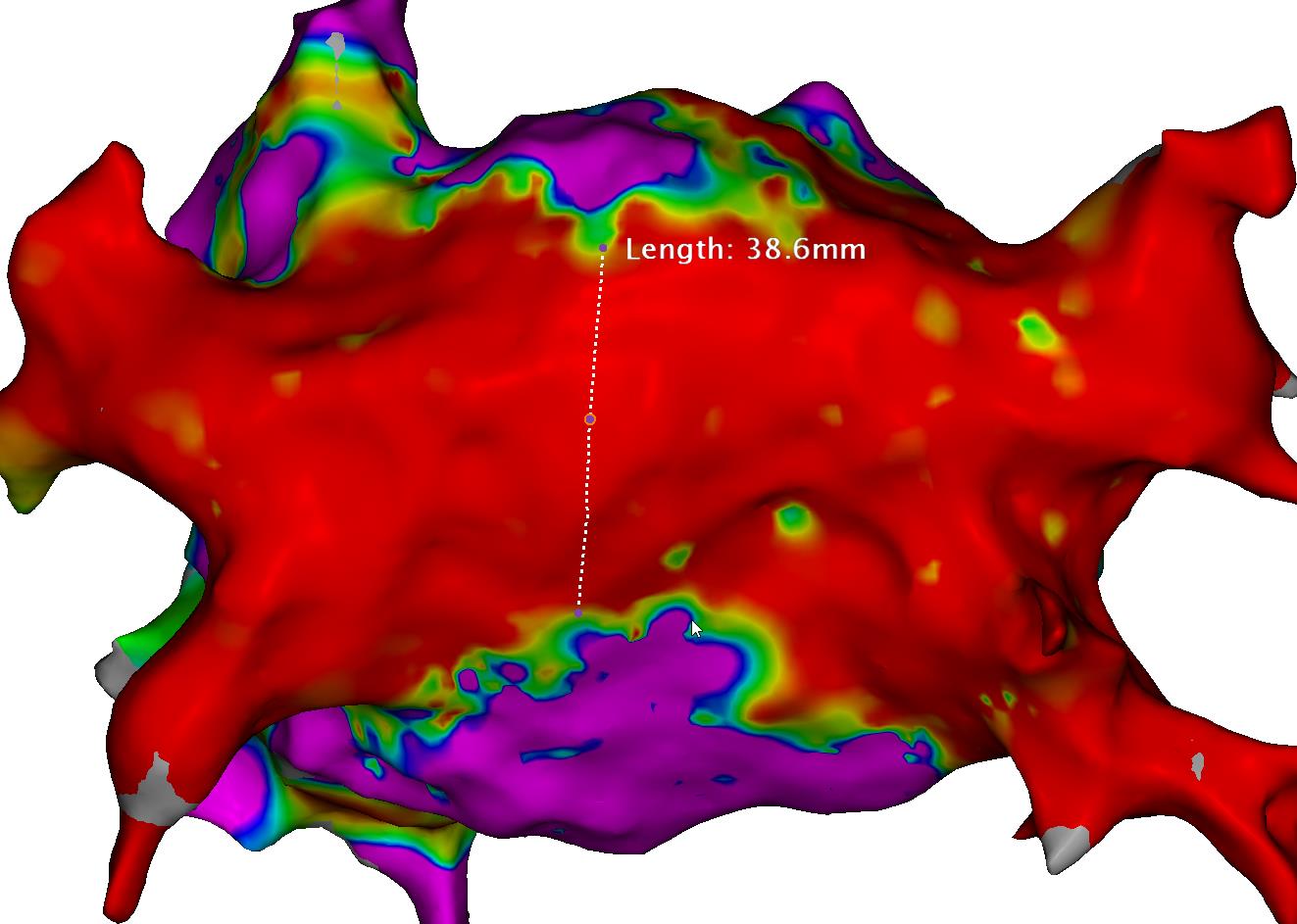 |
| 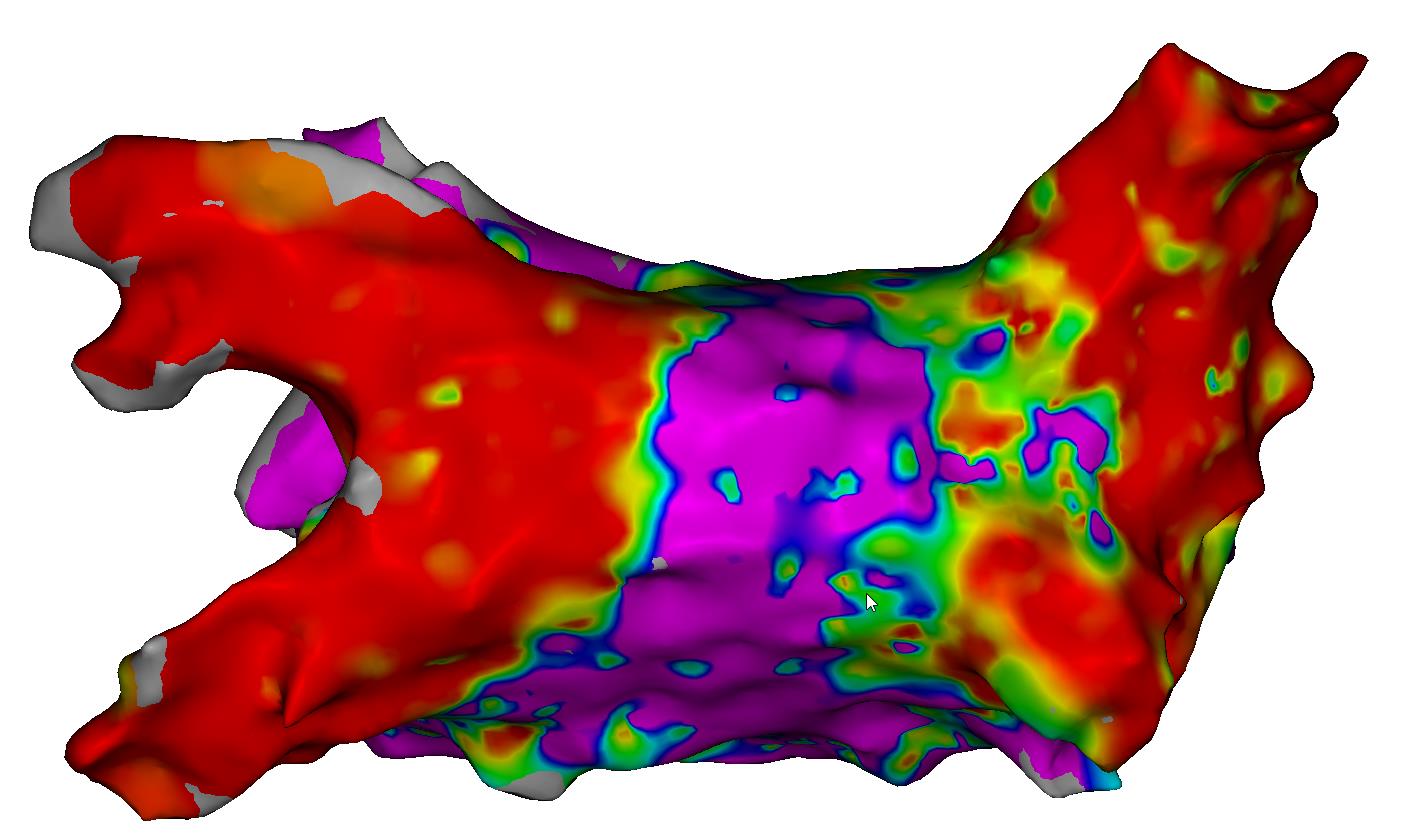 | 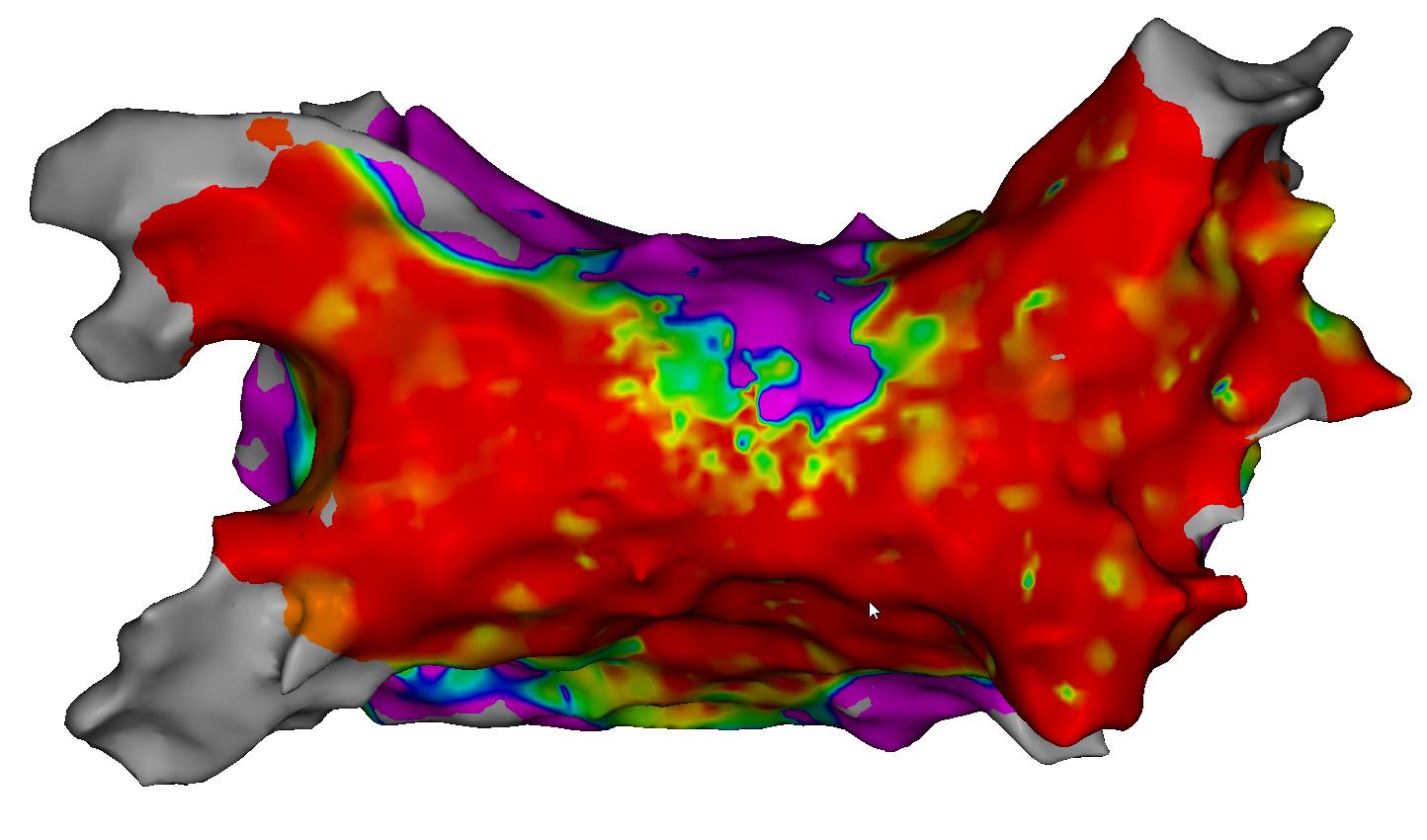 | 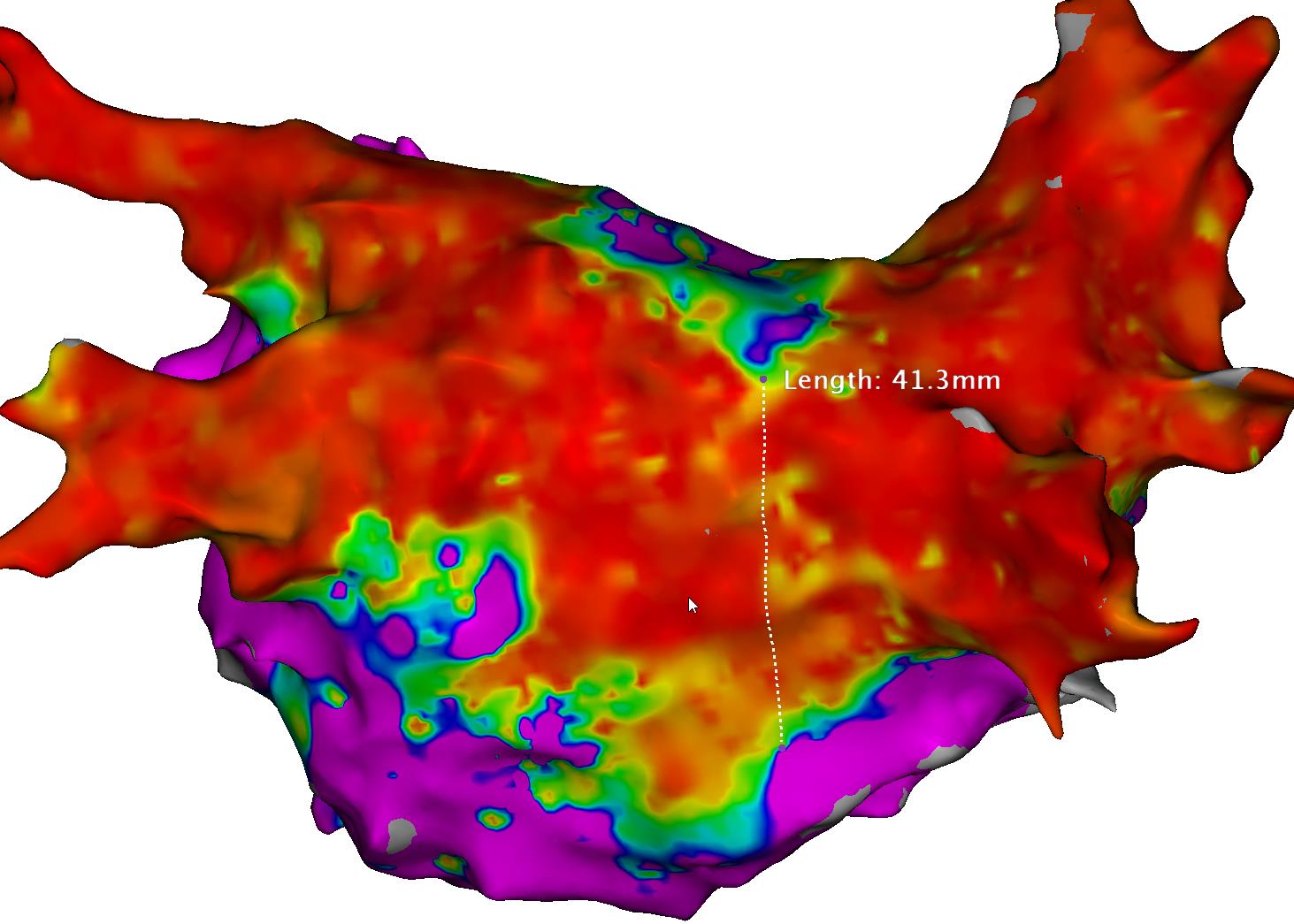 |
| 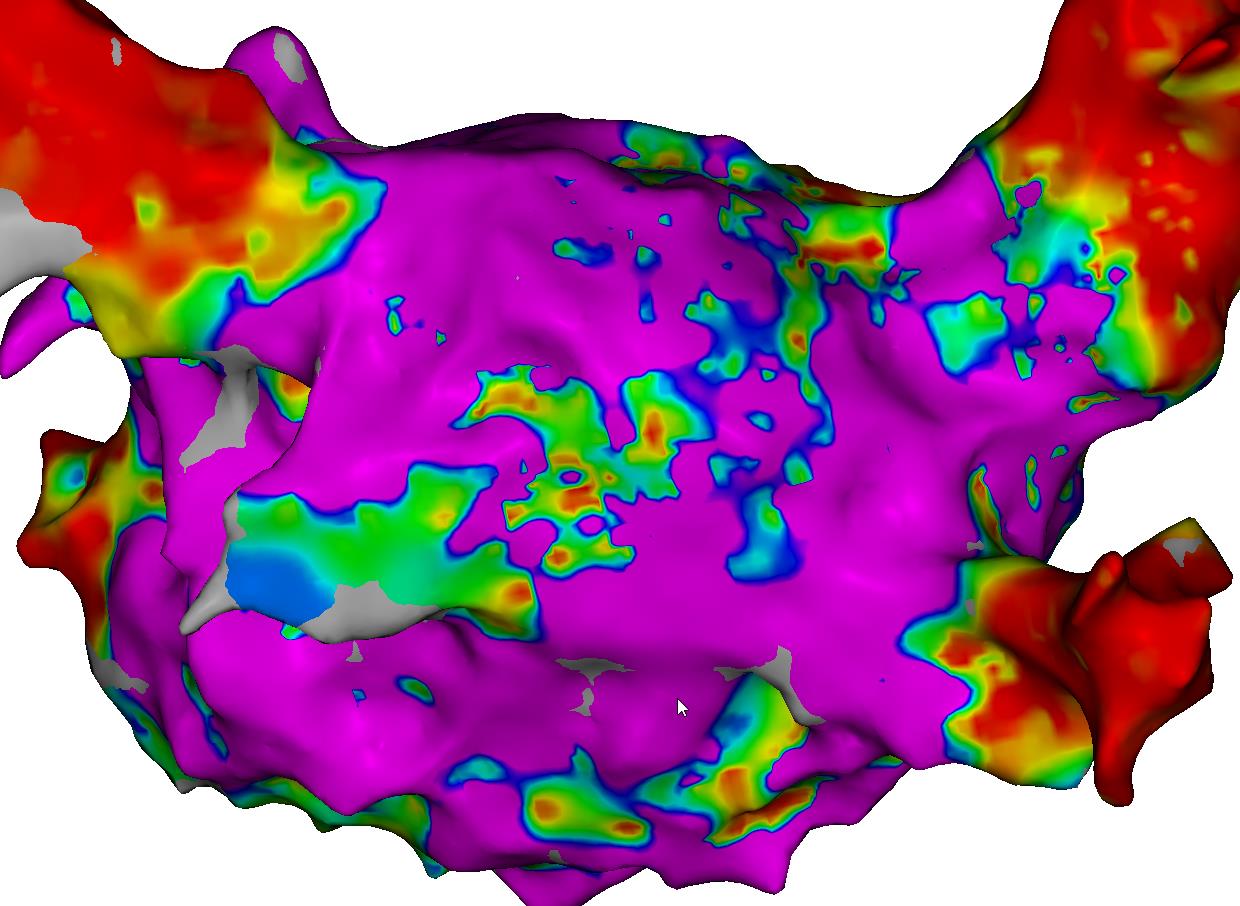 | 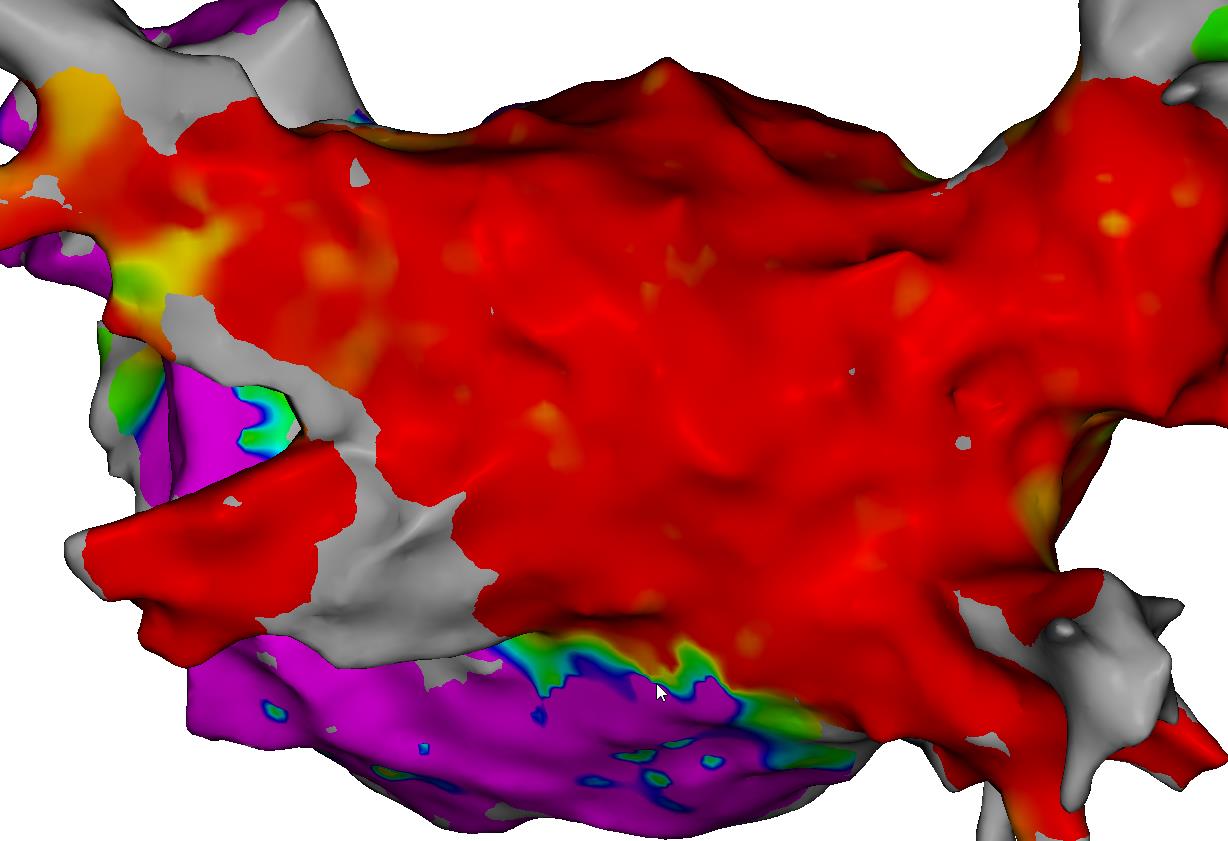 | 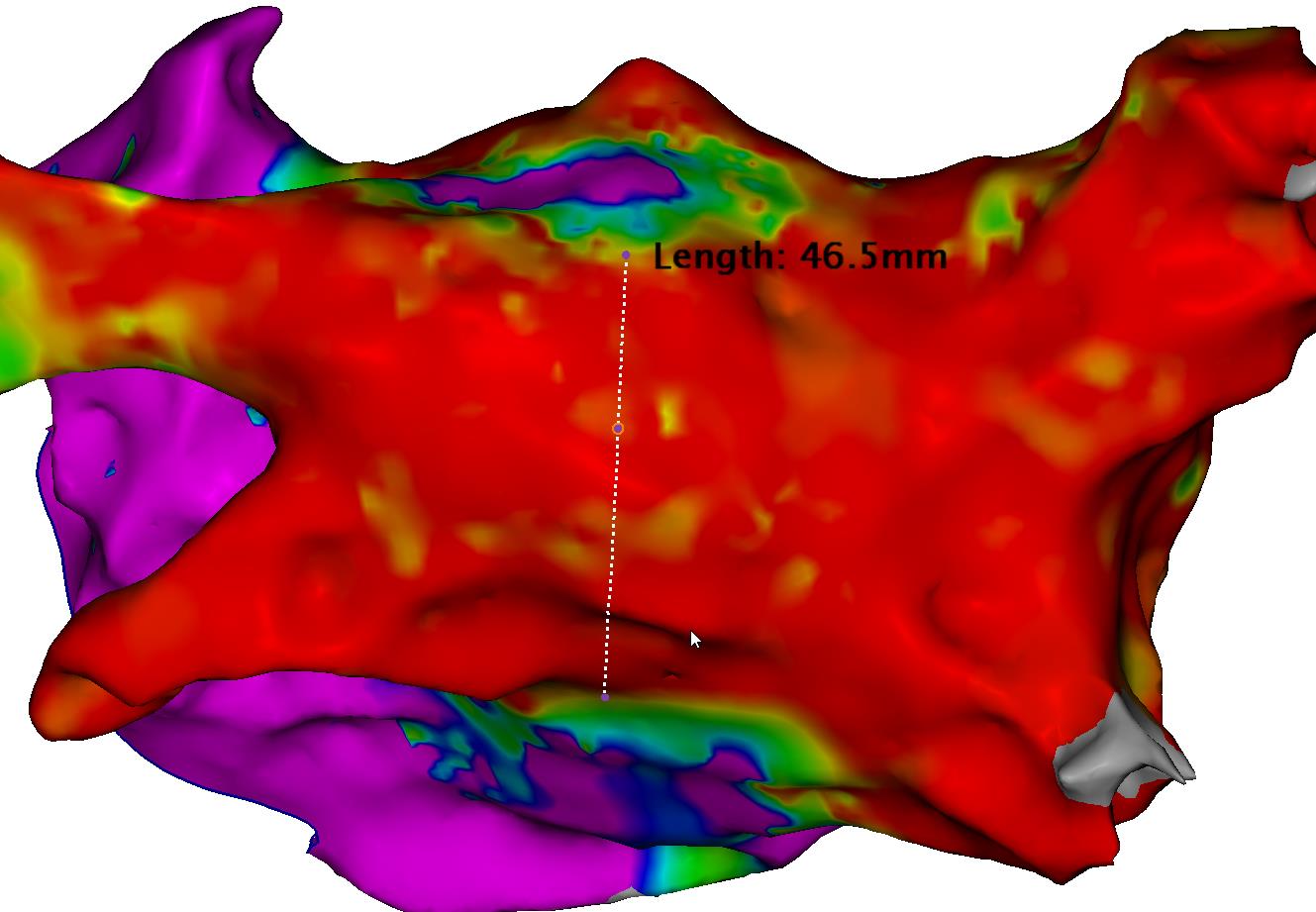 |
| 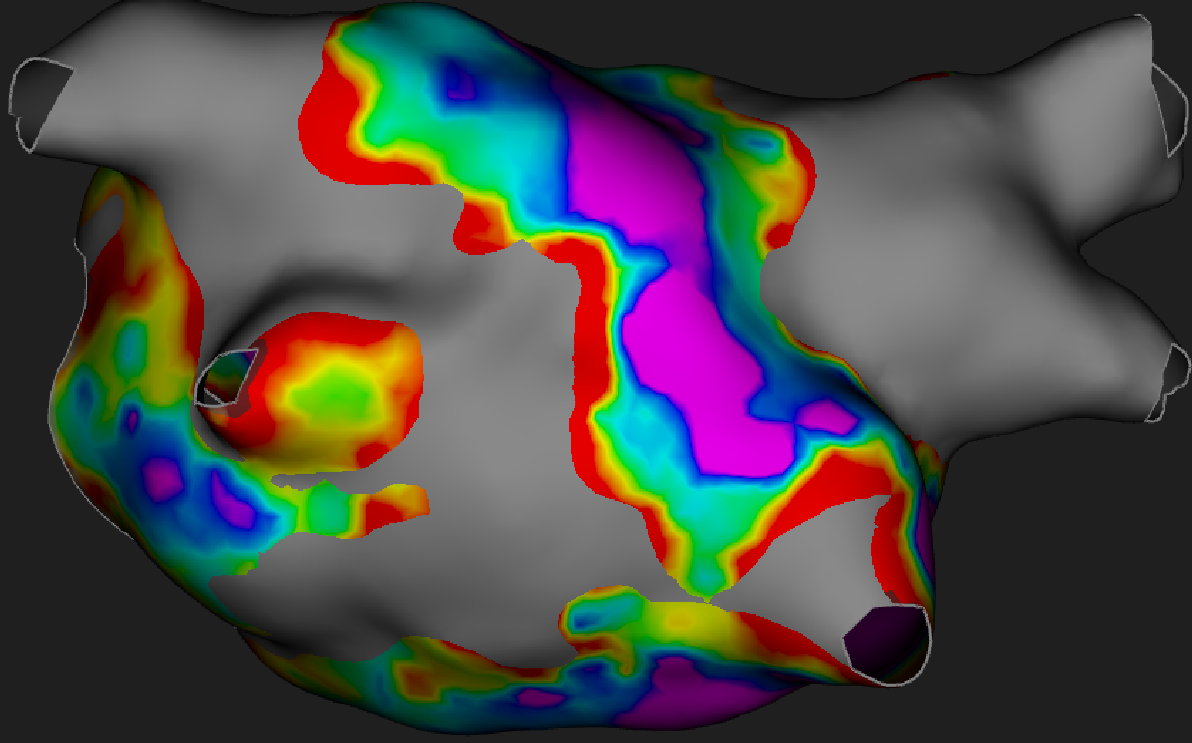 | No map acquired | 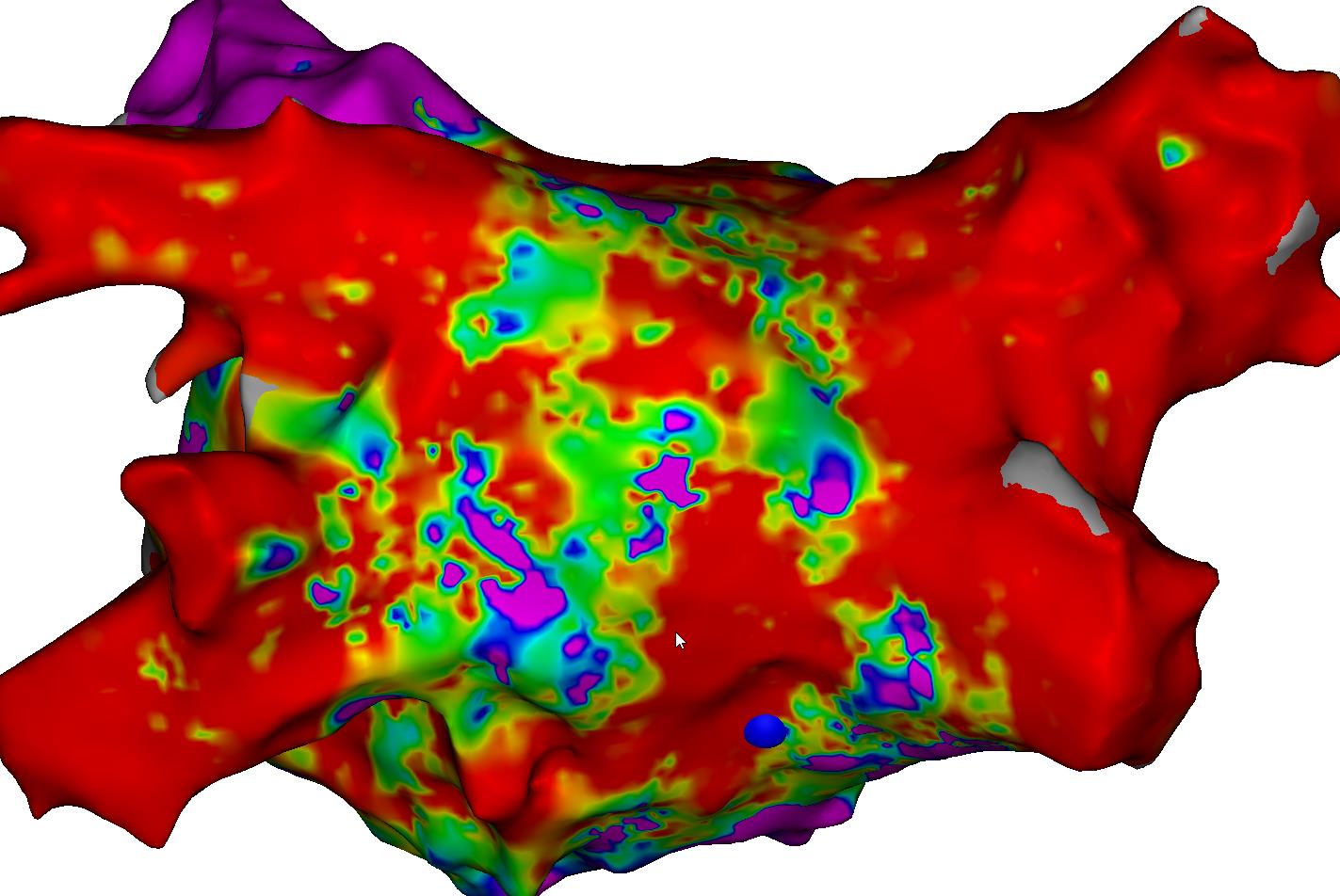 Reconnection, PW-dependent AT |
| 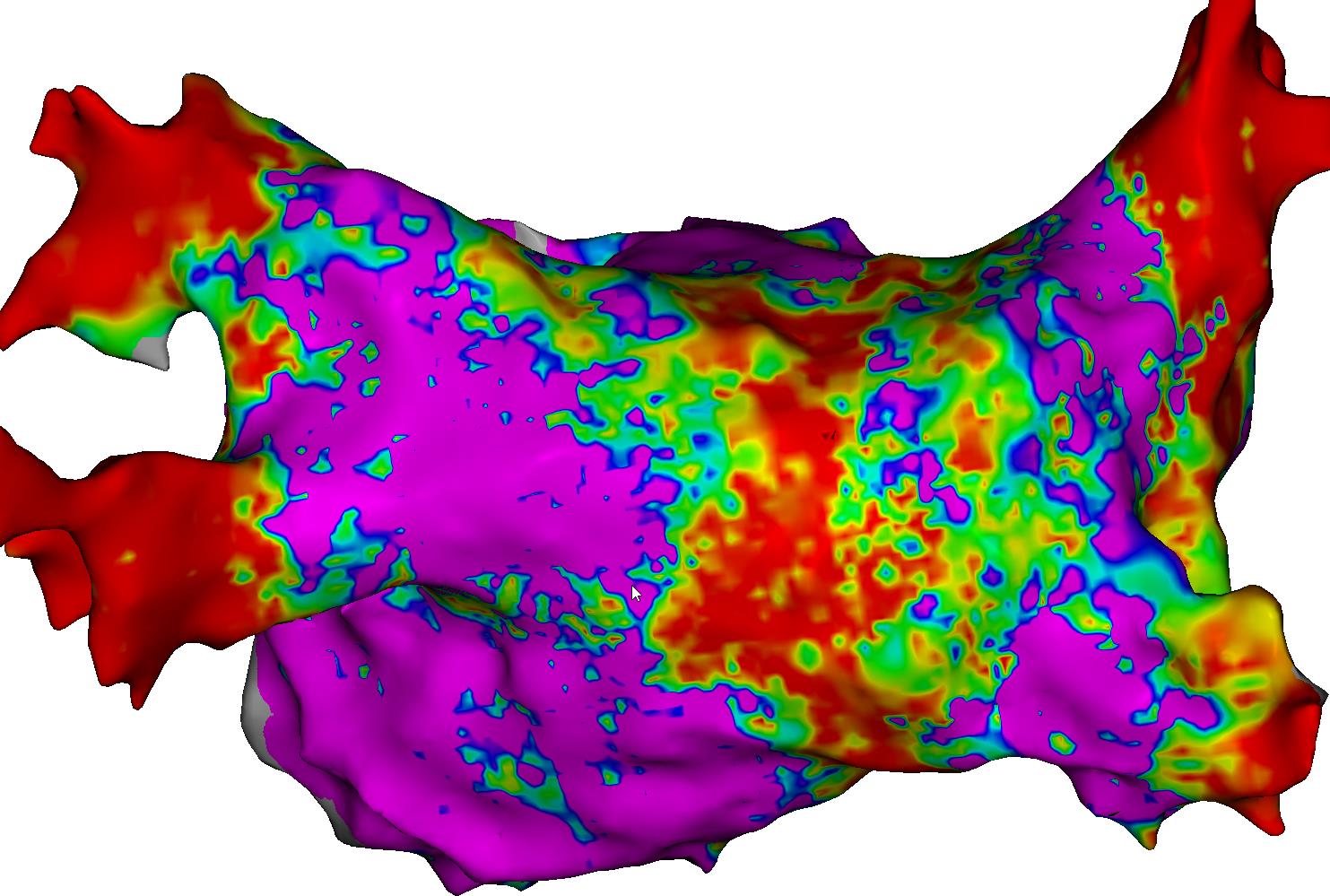 | 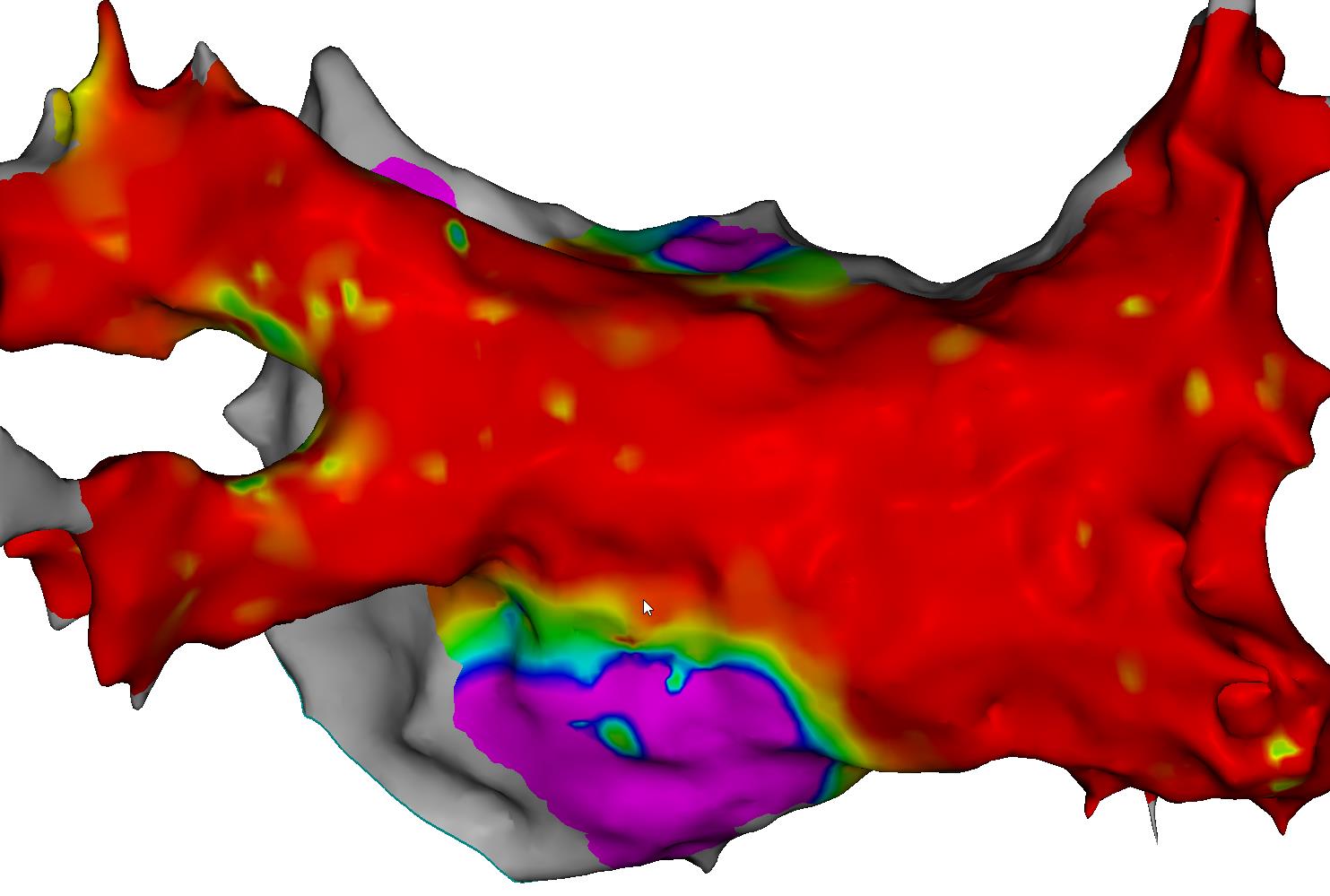 | 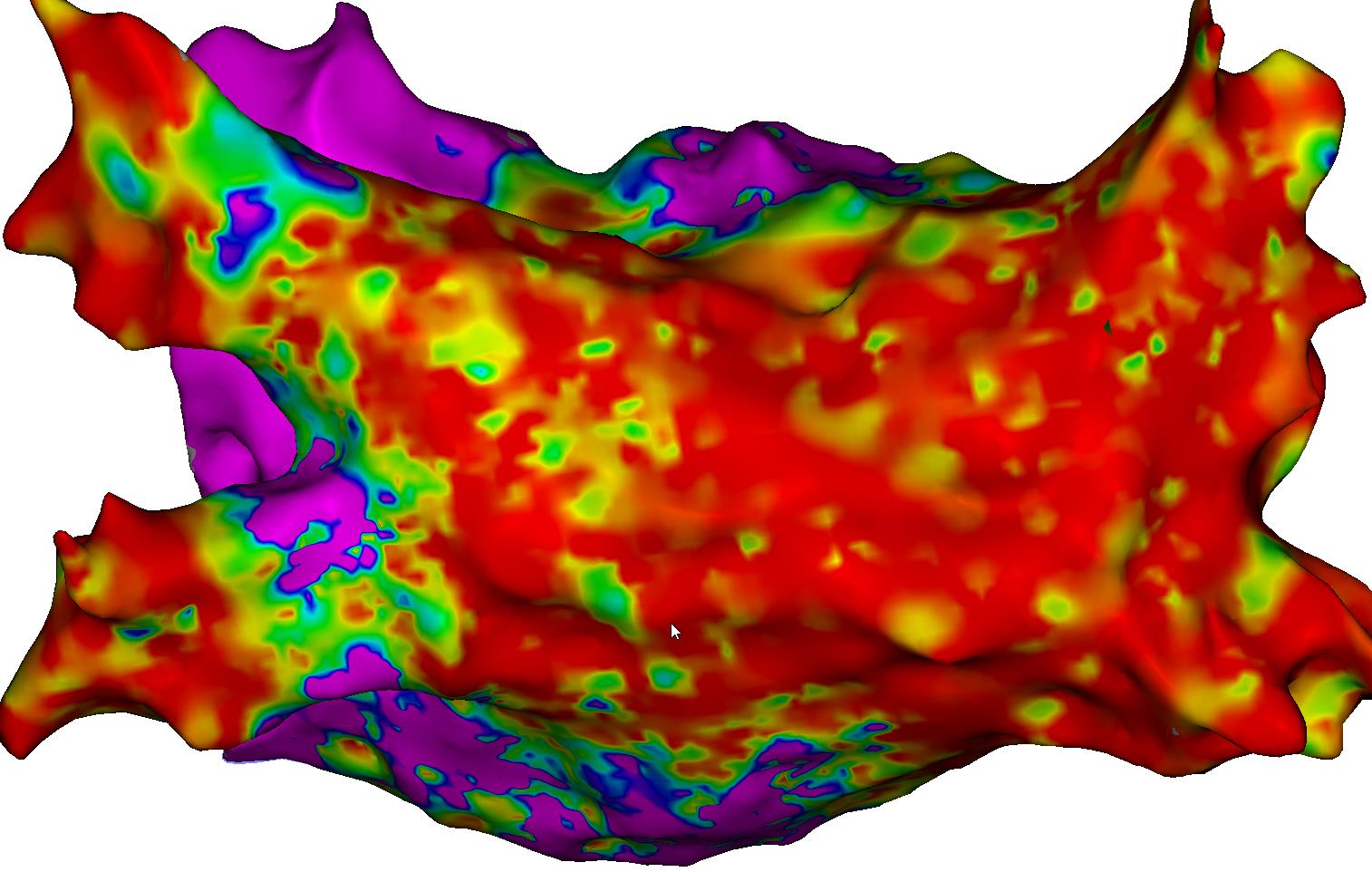 Reconnection, PW-dependent AT |
| 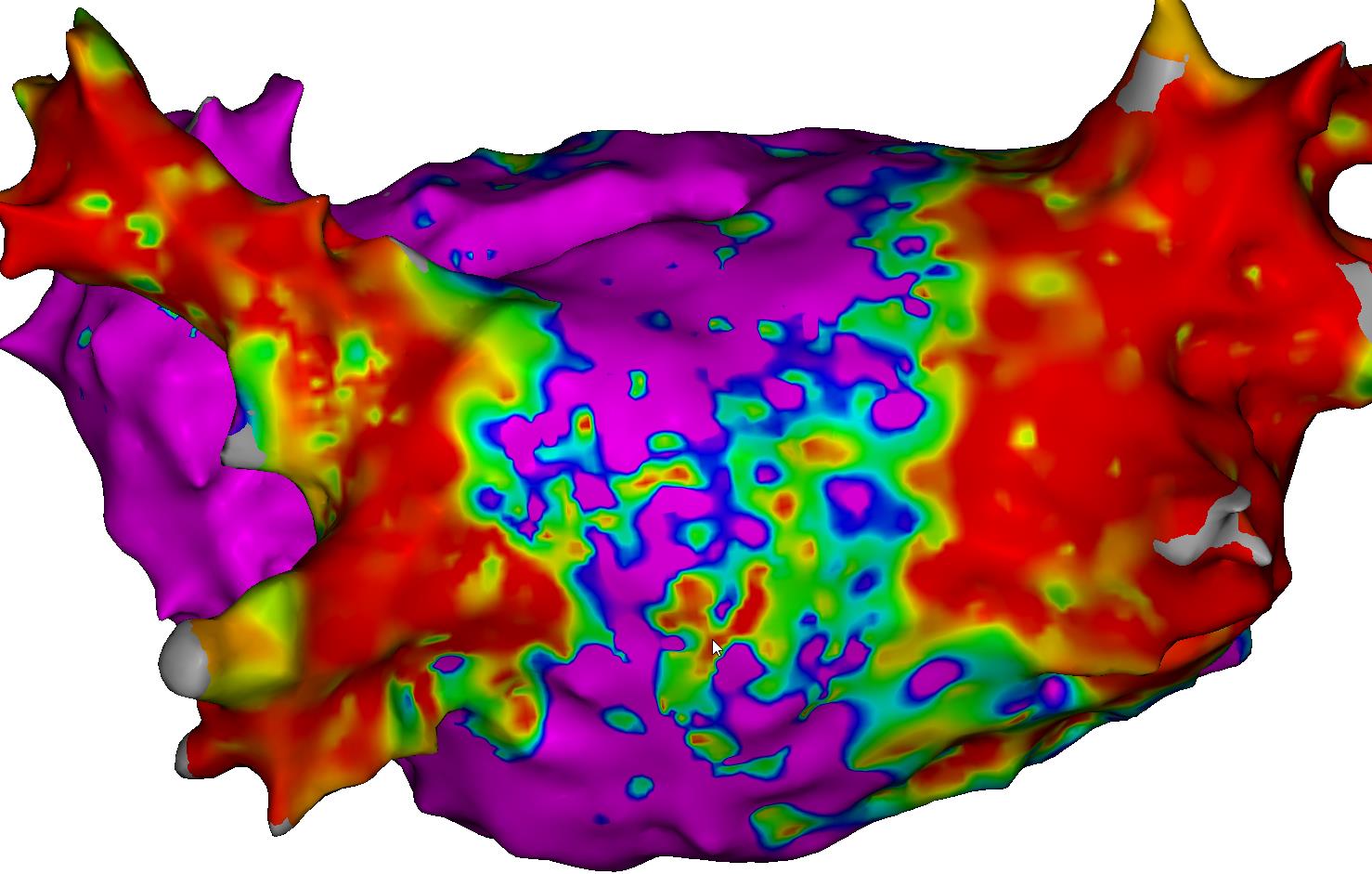 | 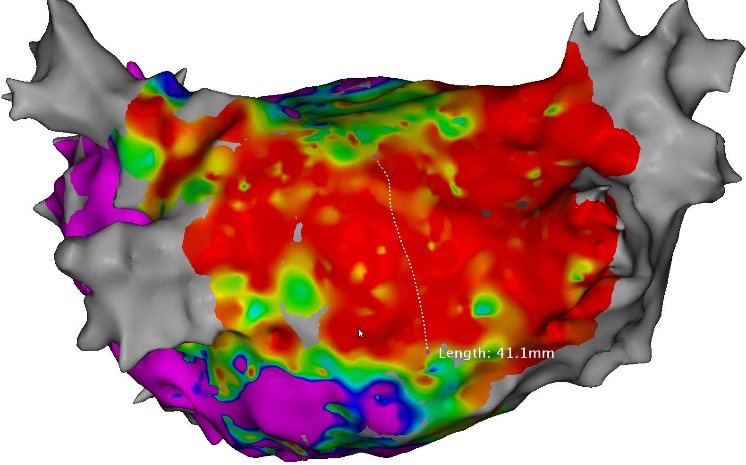 | 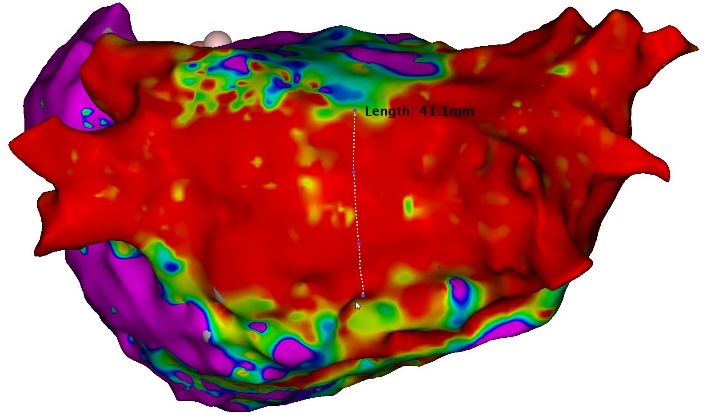 |
|  |  |  |
| 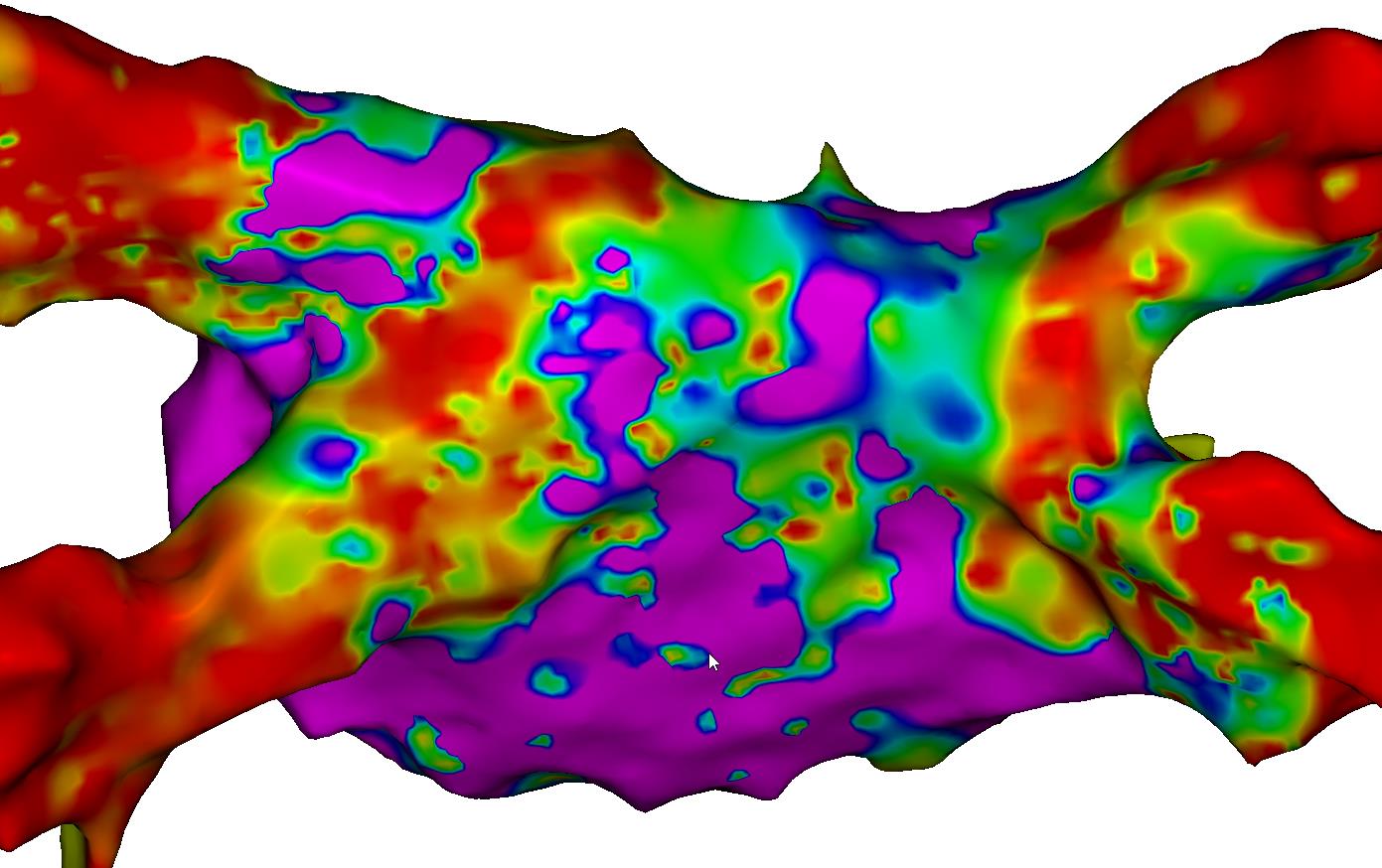 | 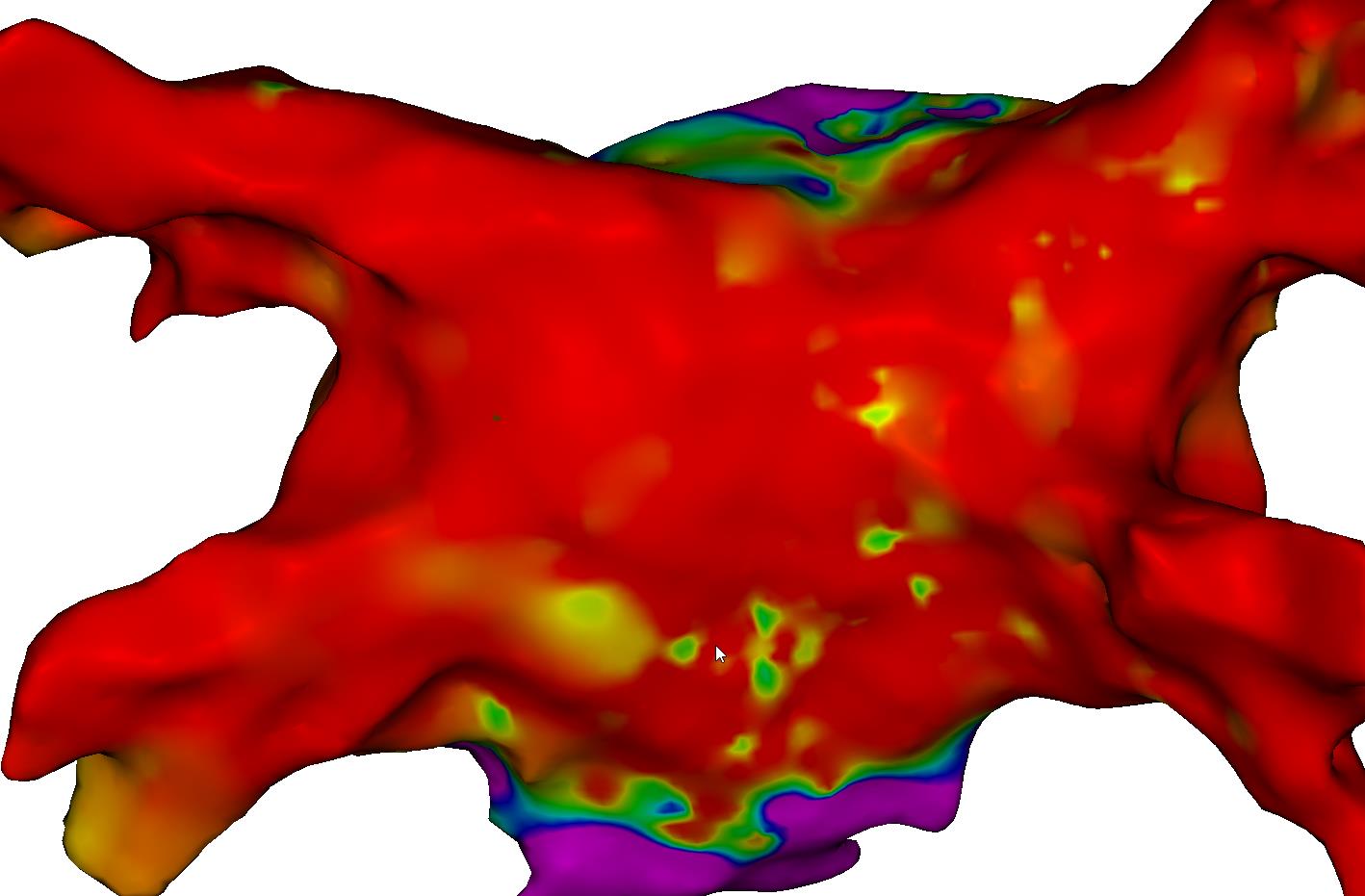 | 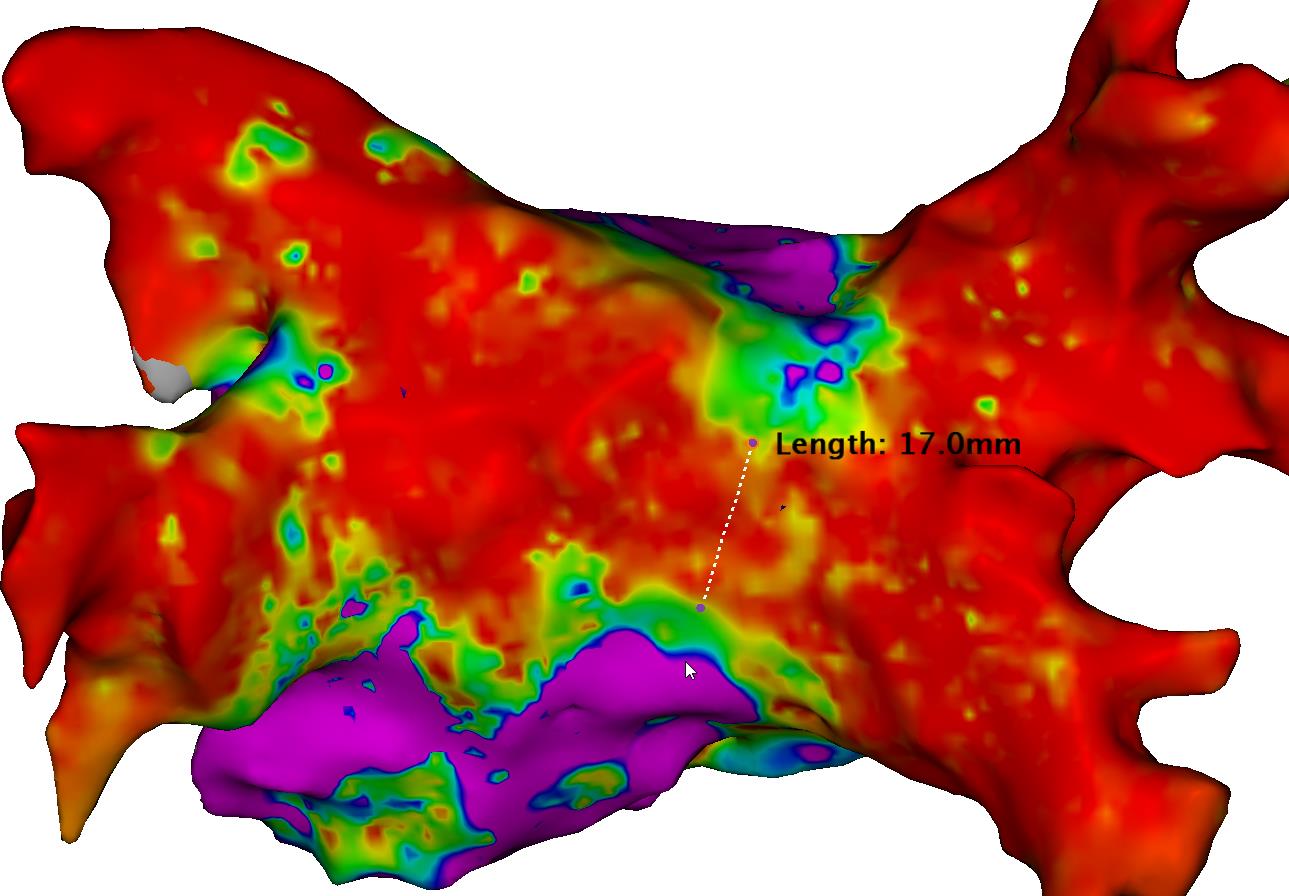 Regression but no reconnection |
| 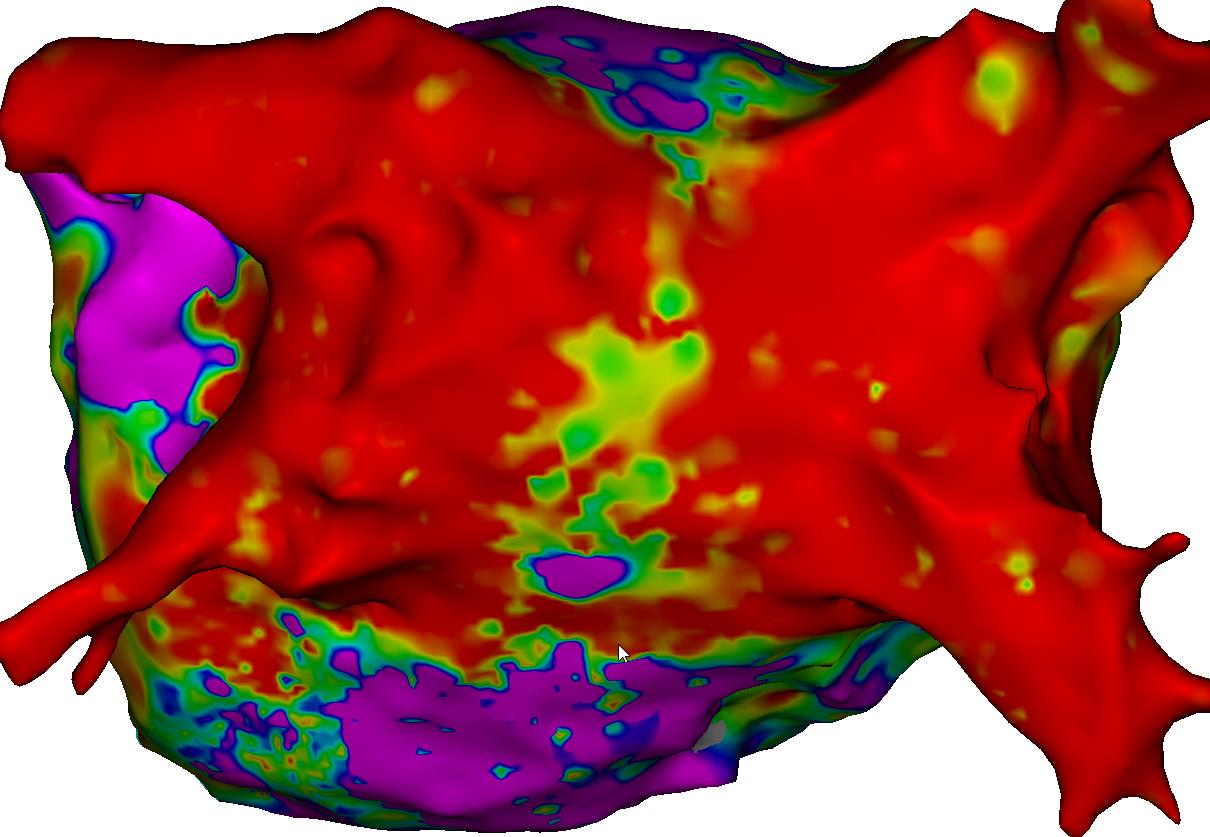 | 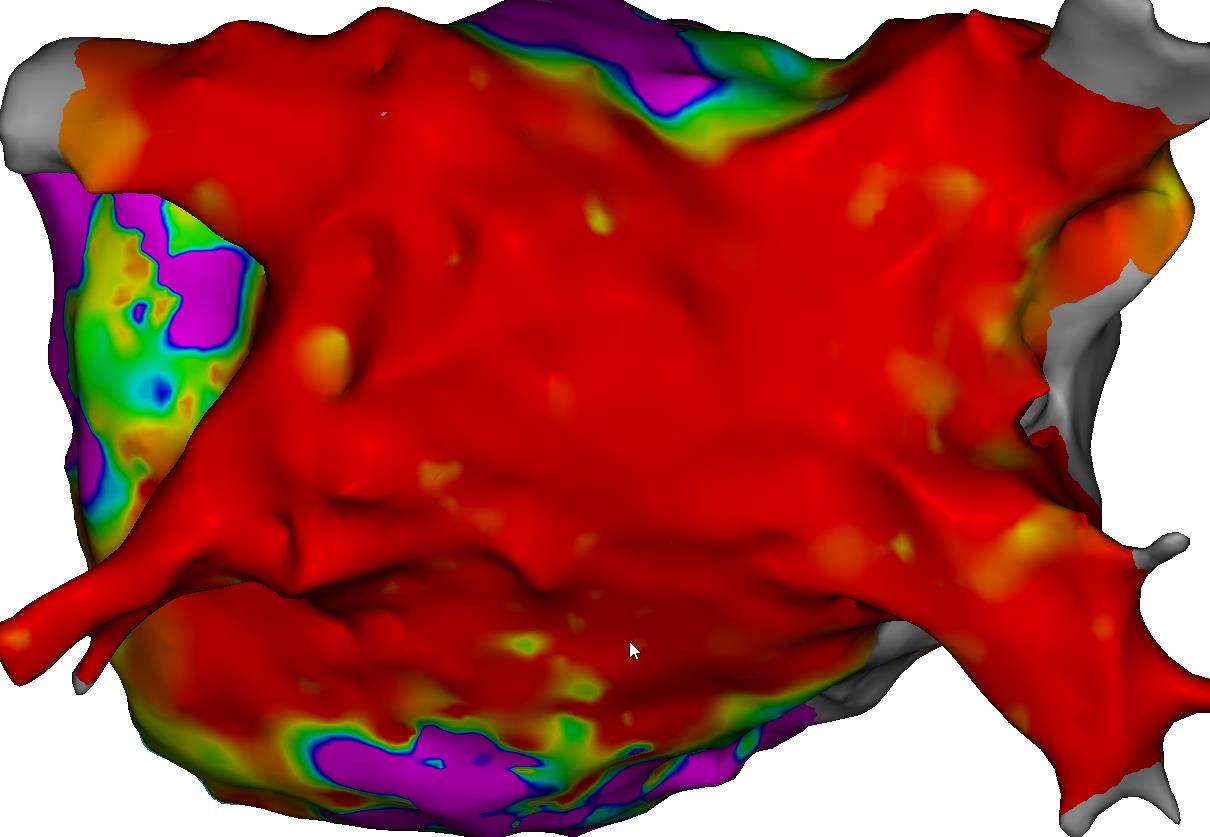 | 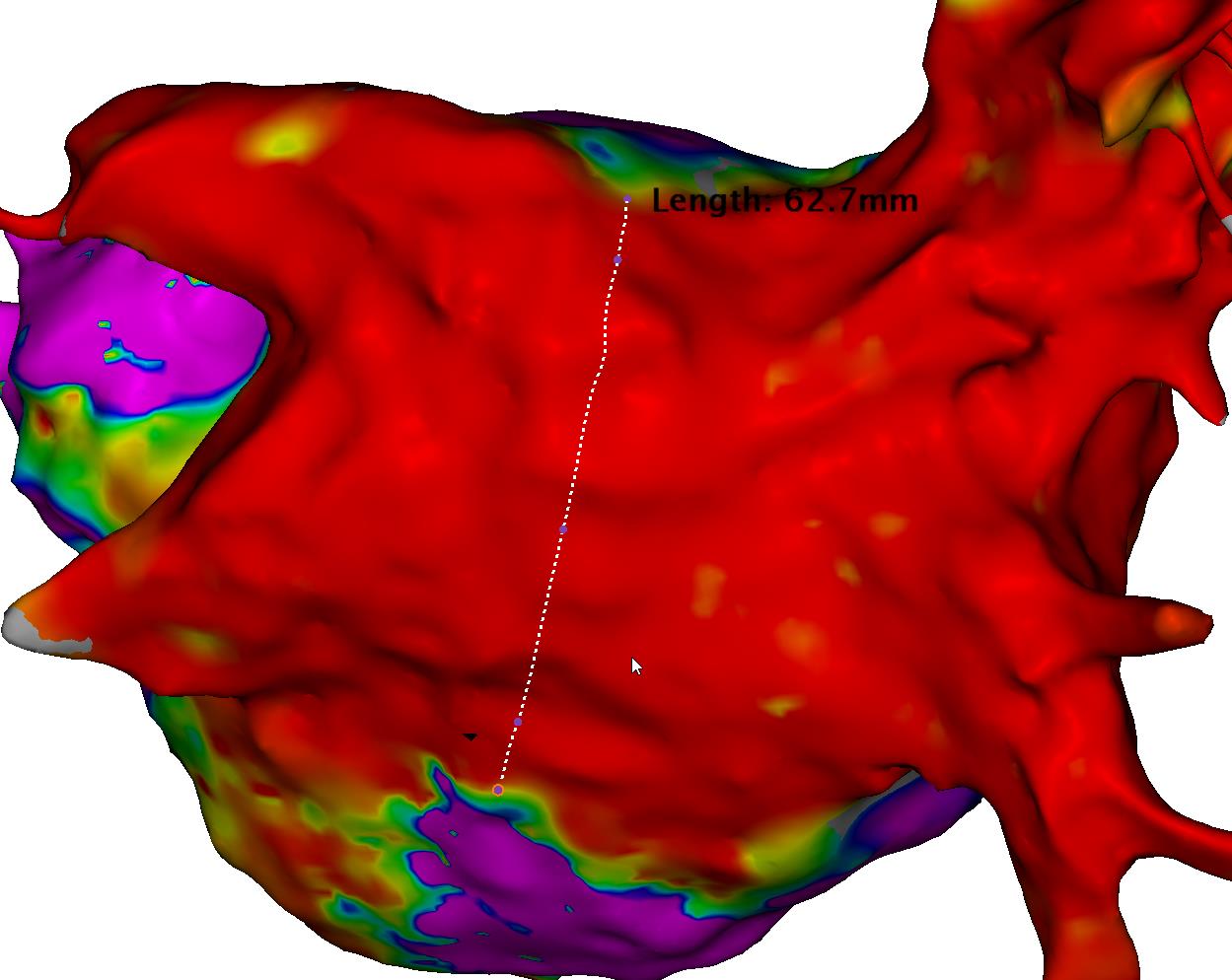 |
| 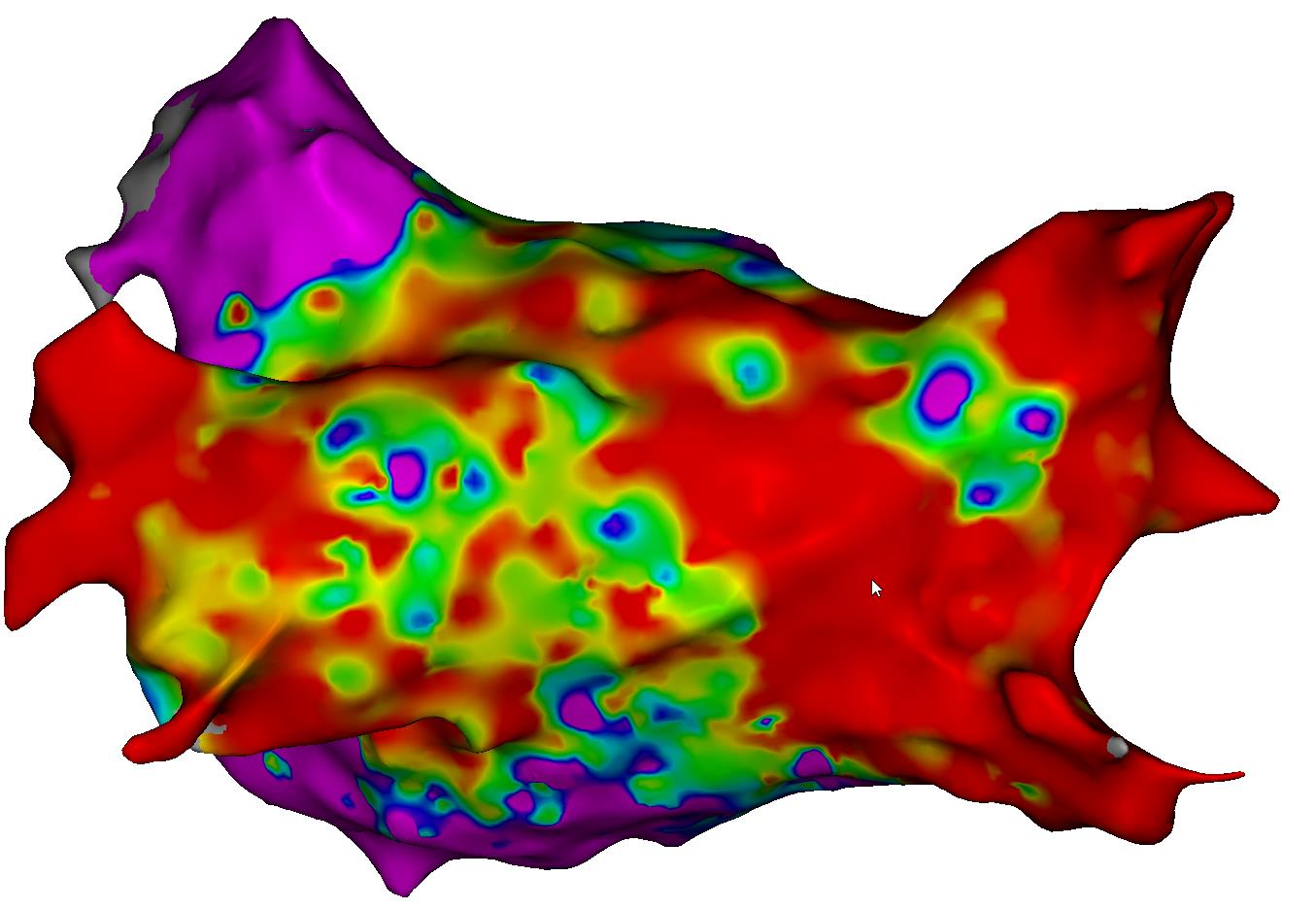 | 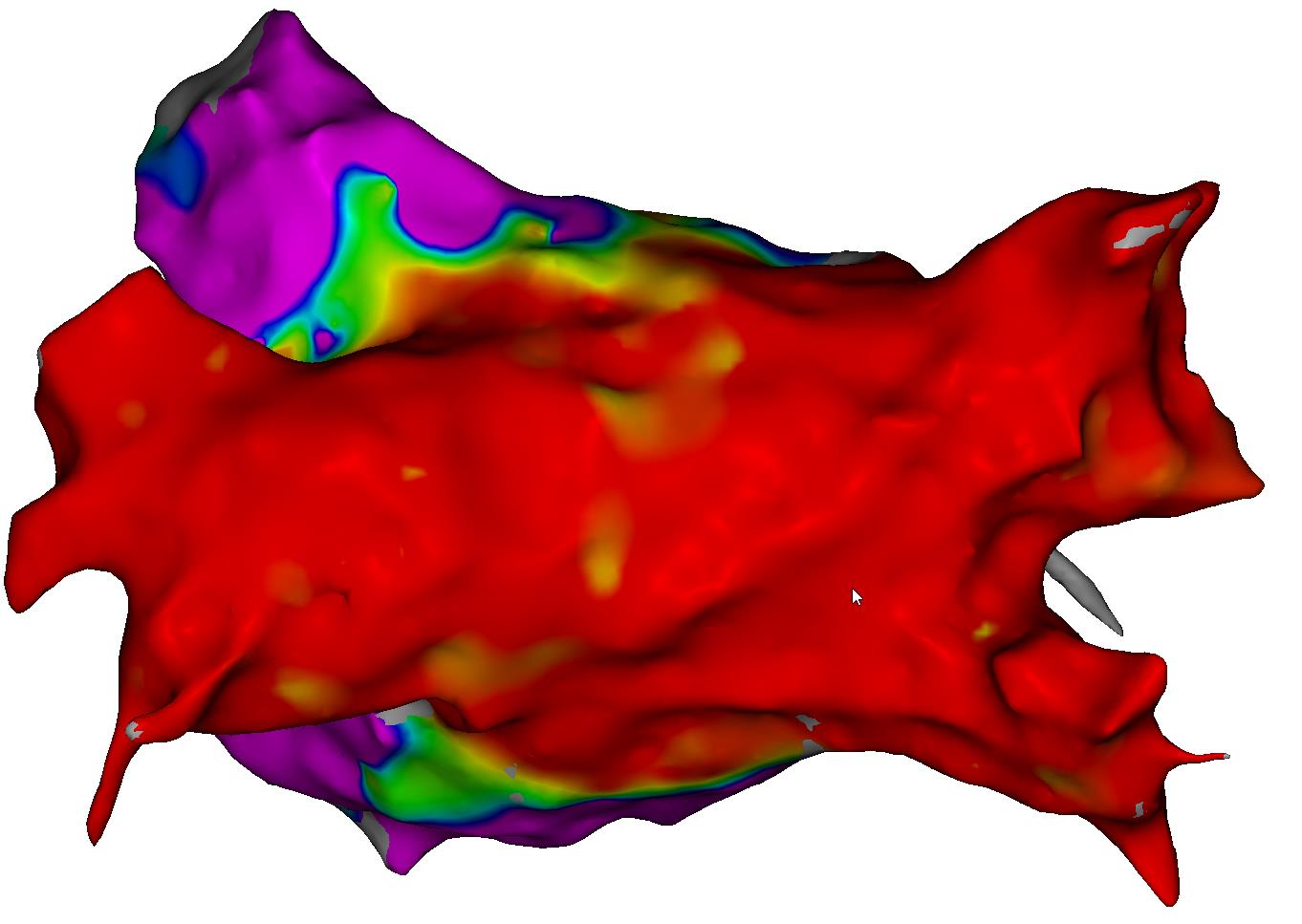 | 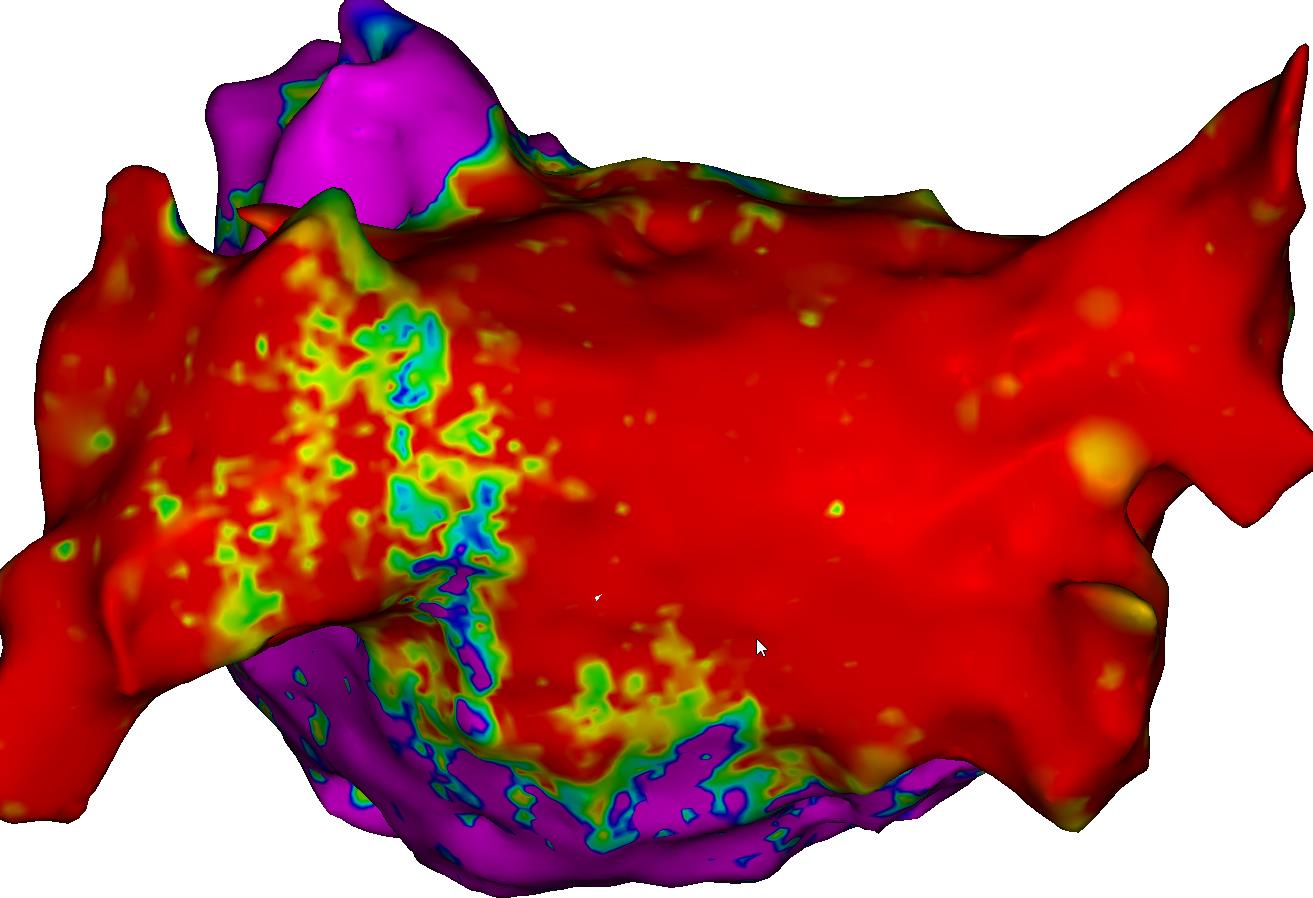 Regression but no reconnection |
| 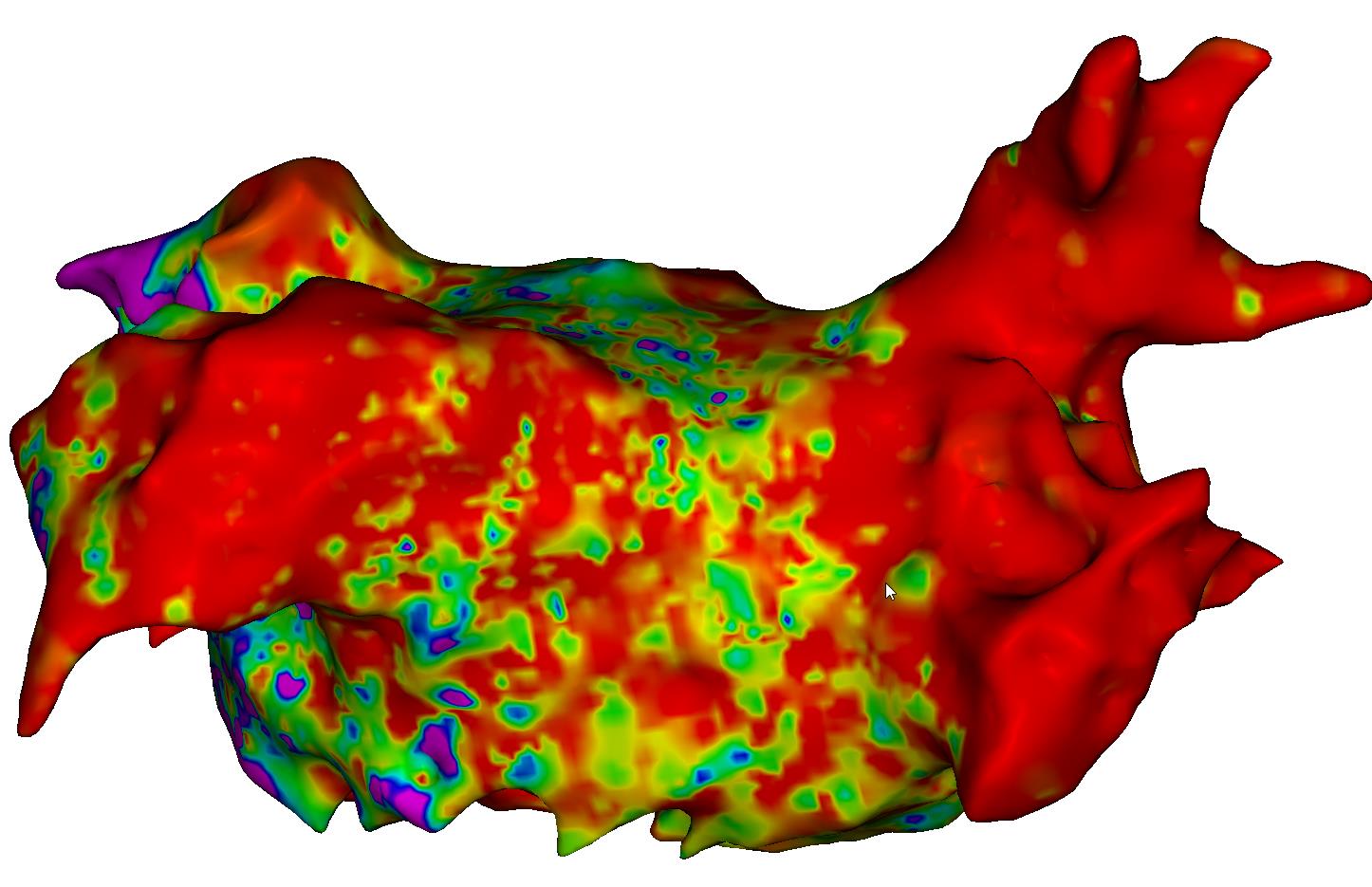 | 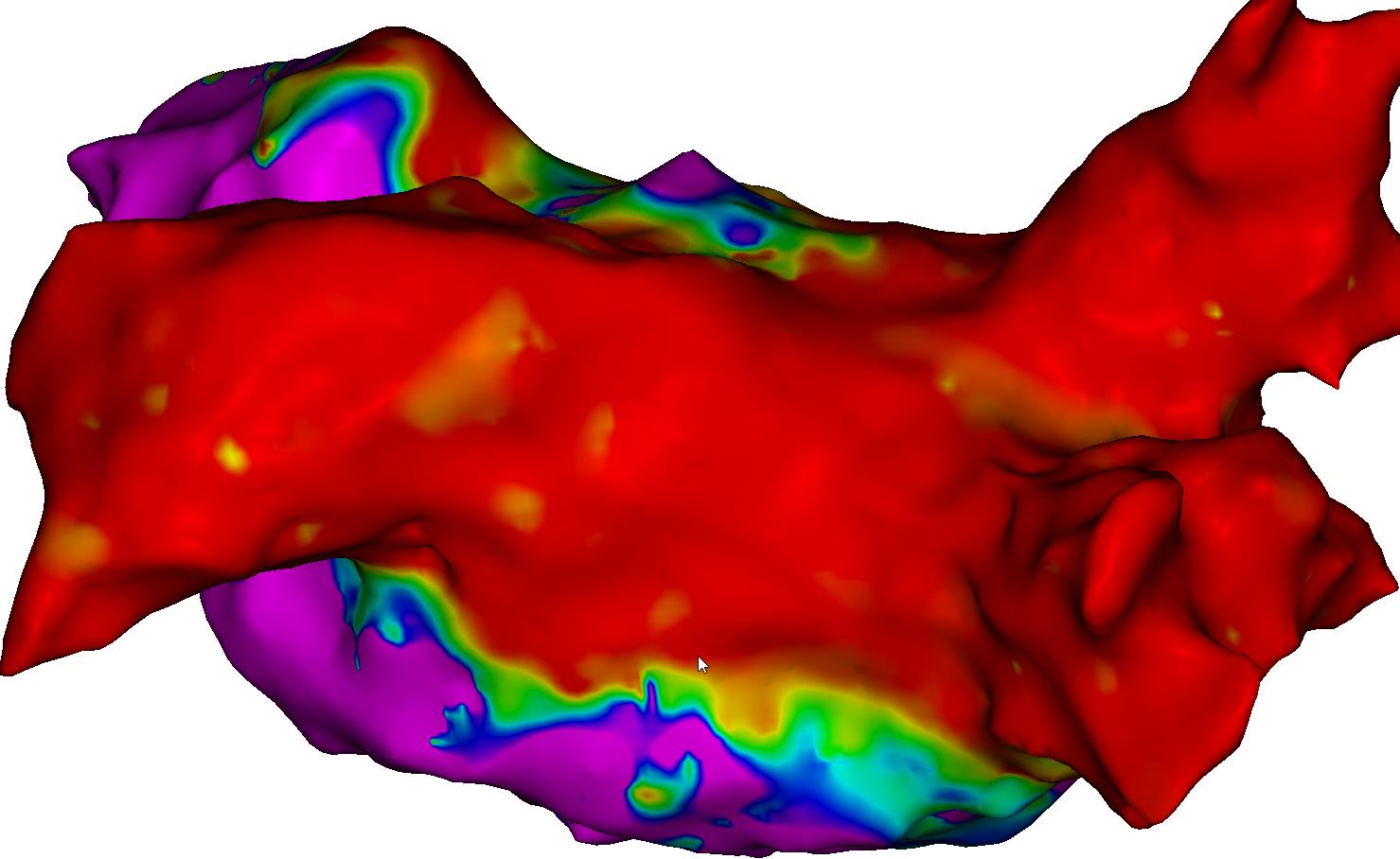 | 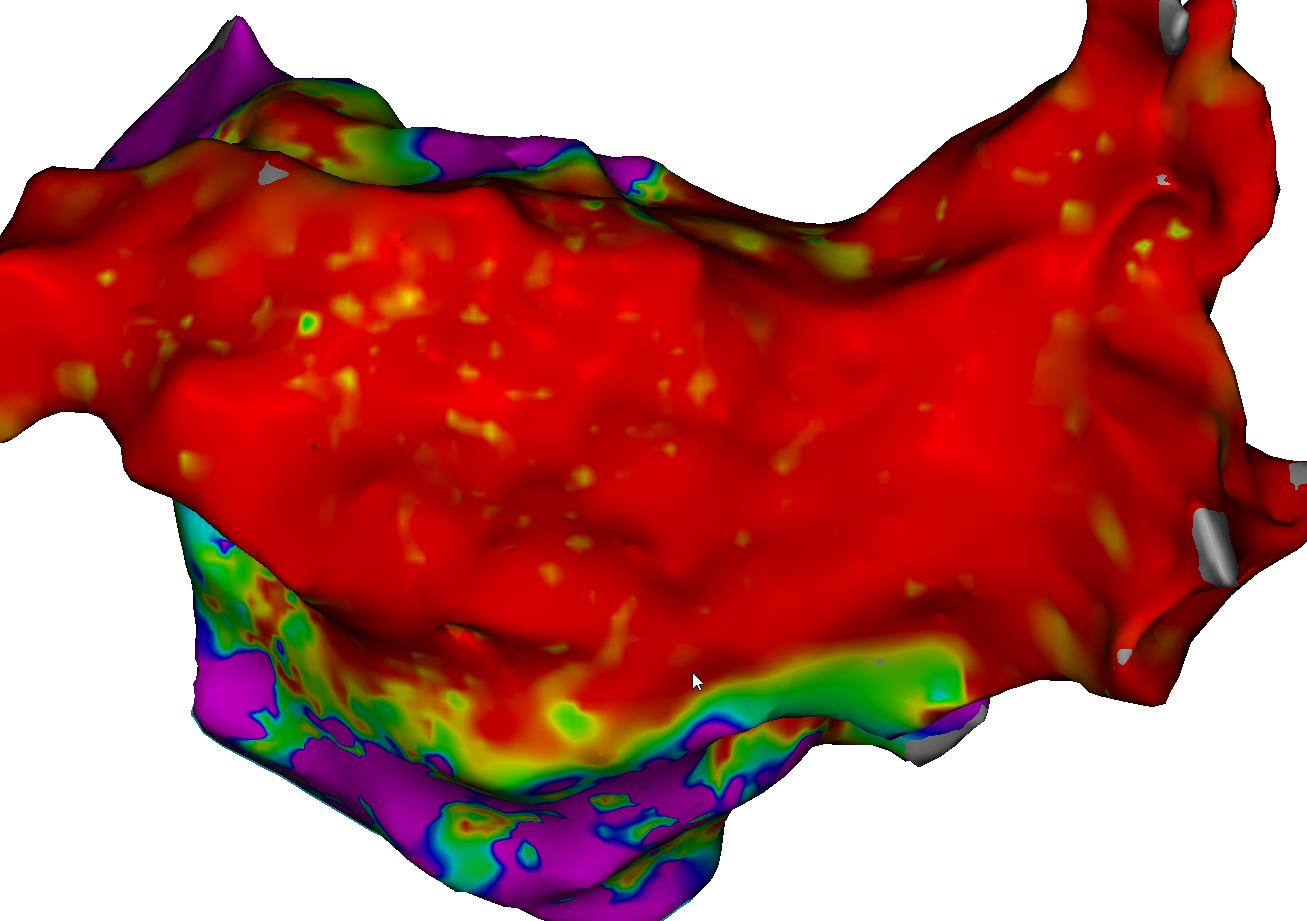 |
| 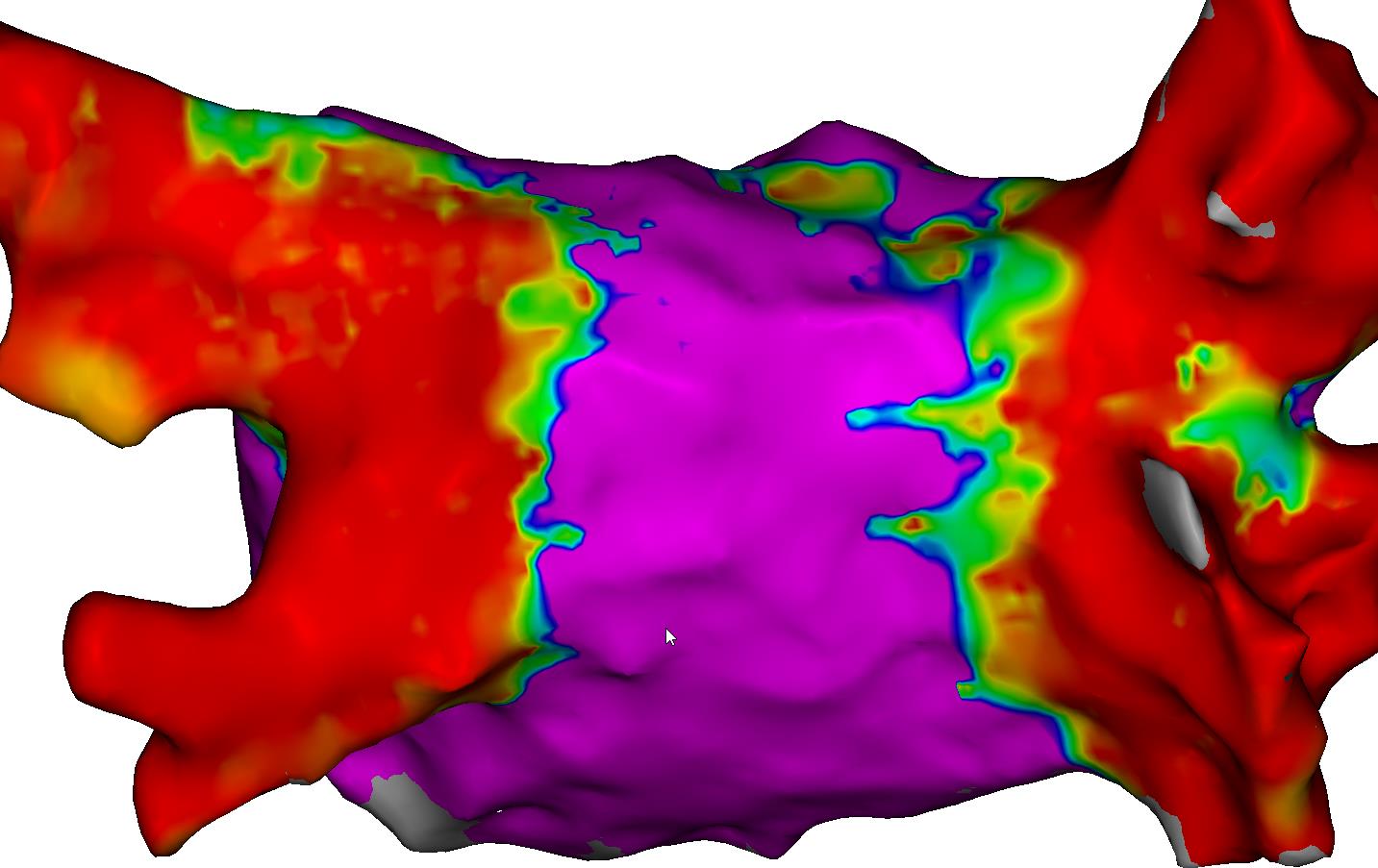 | 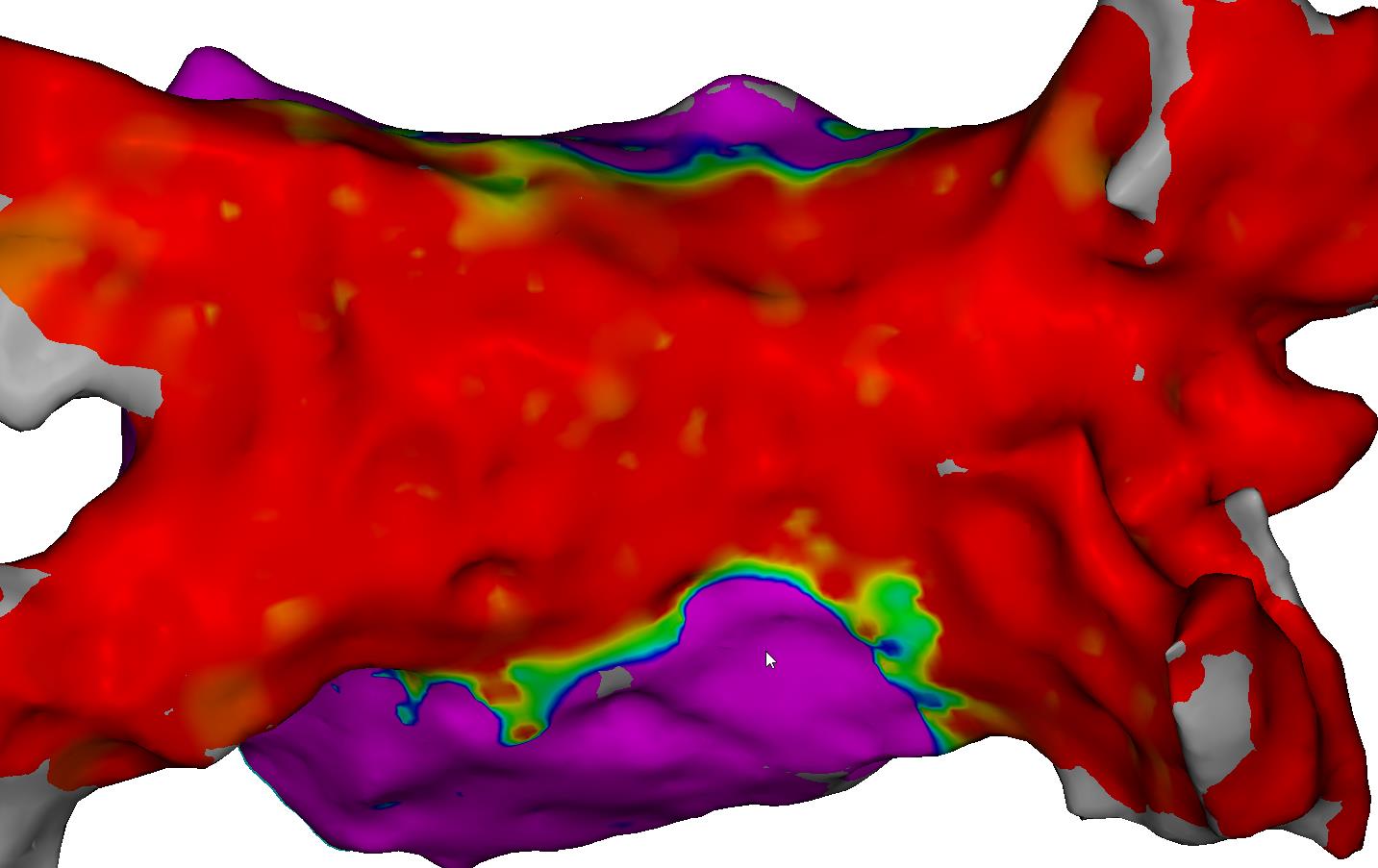 | 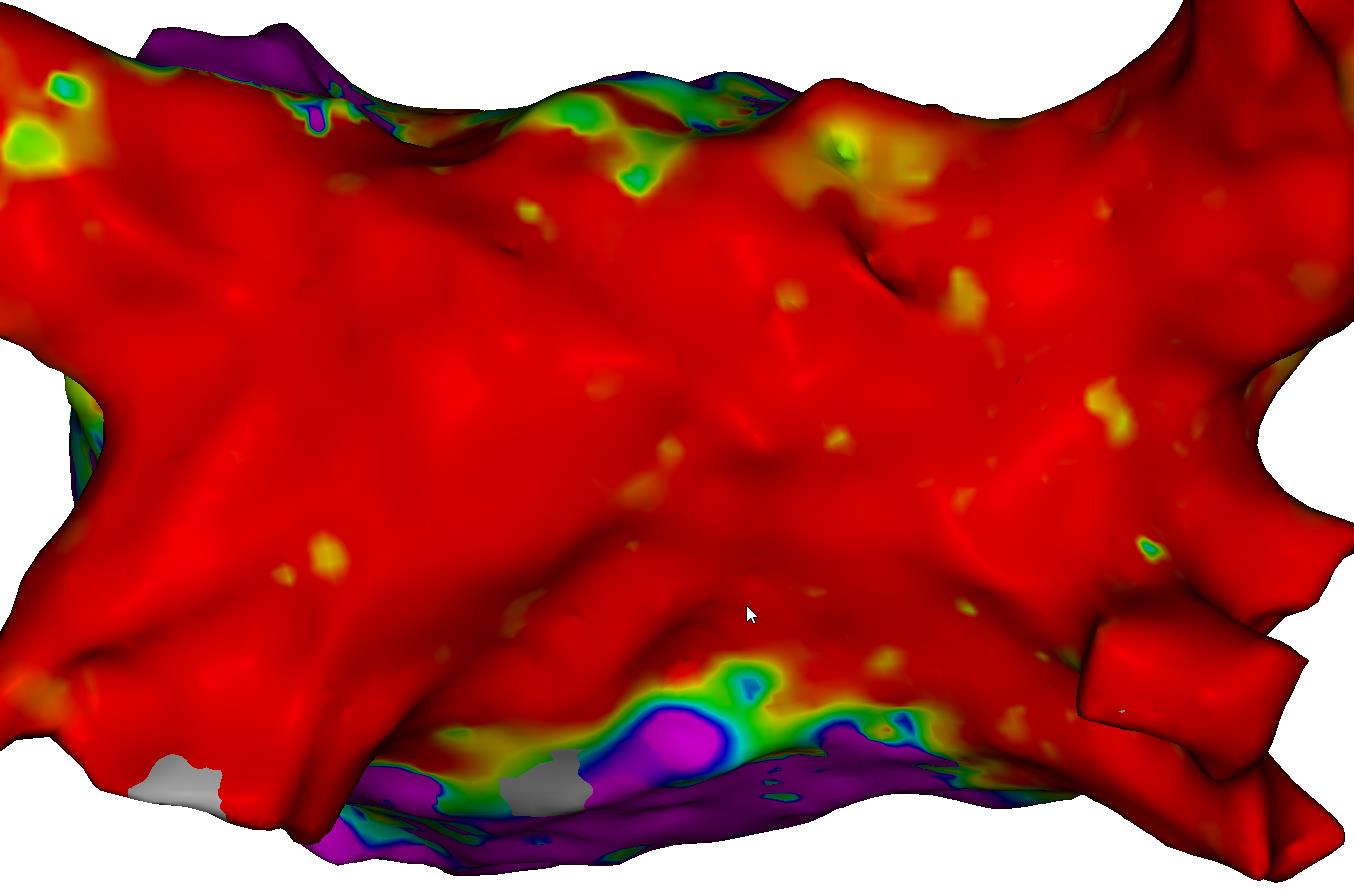 |
| 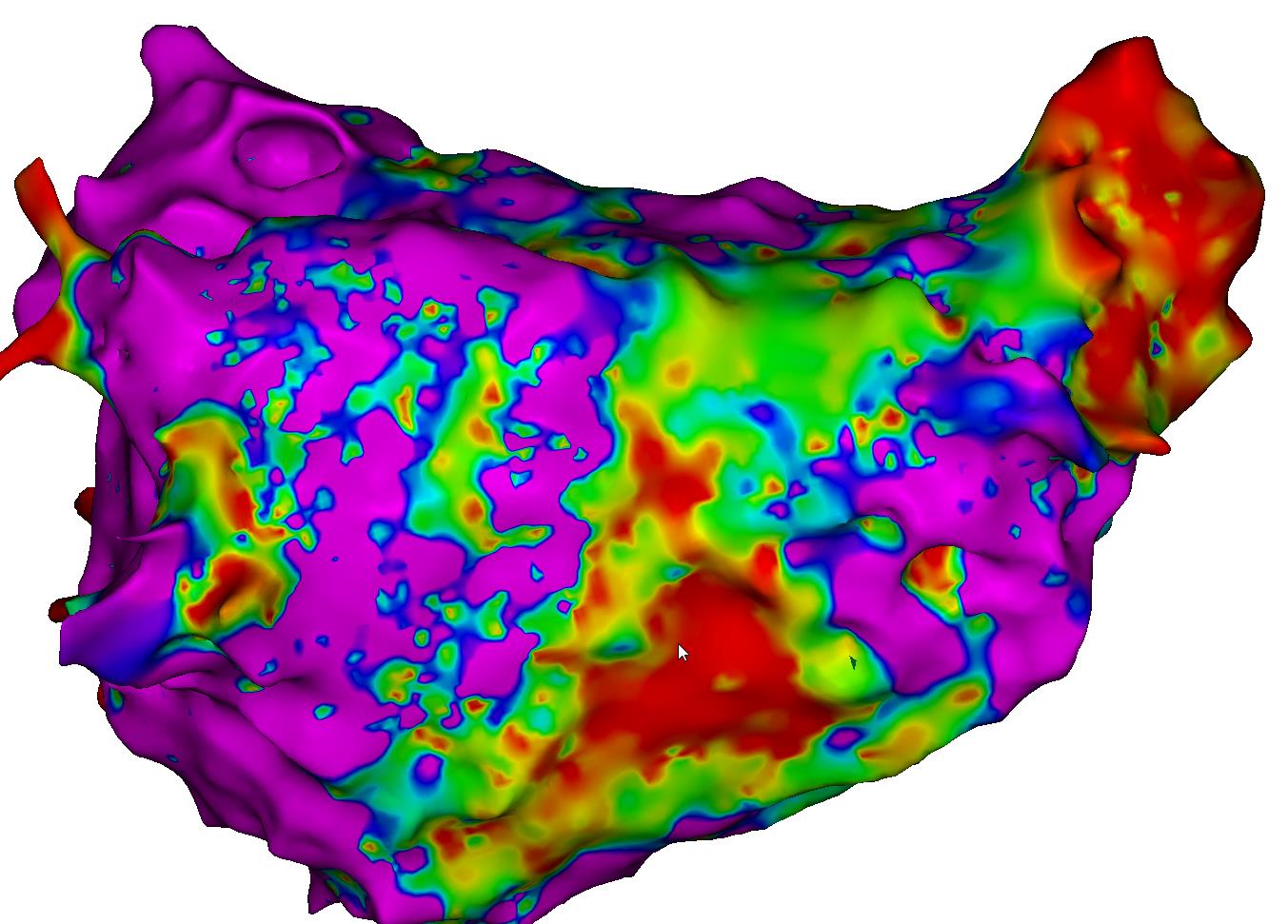 | 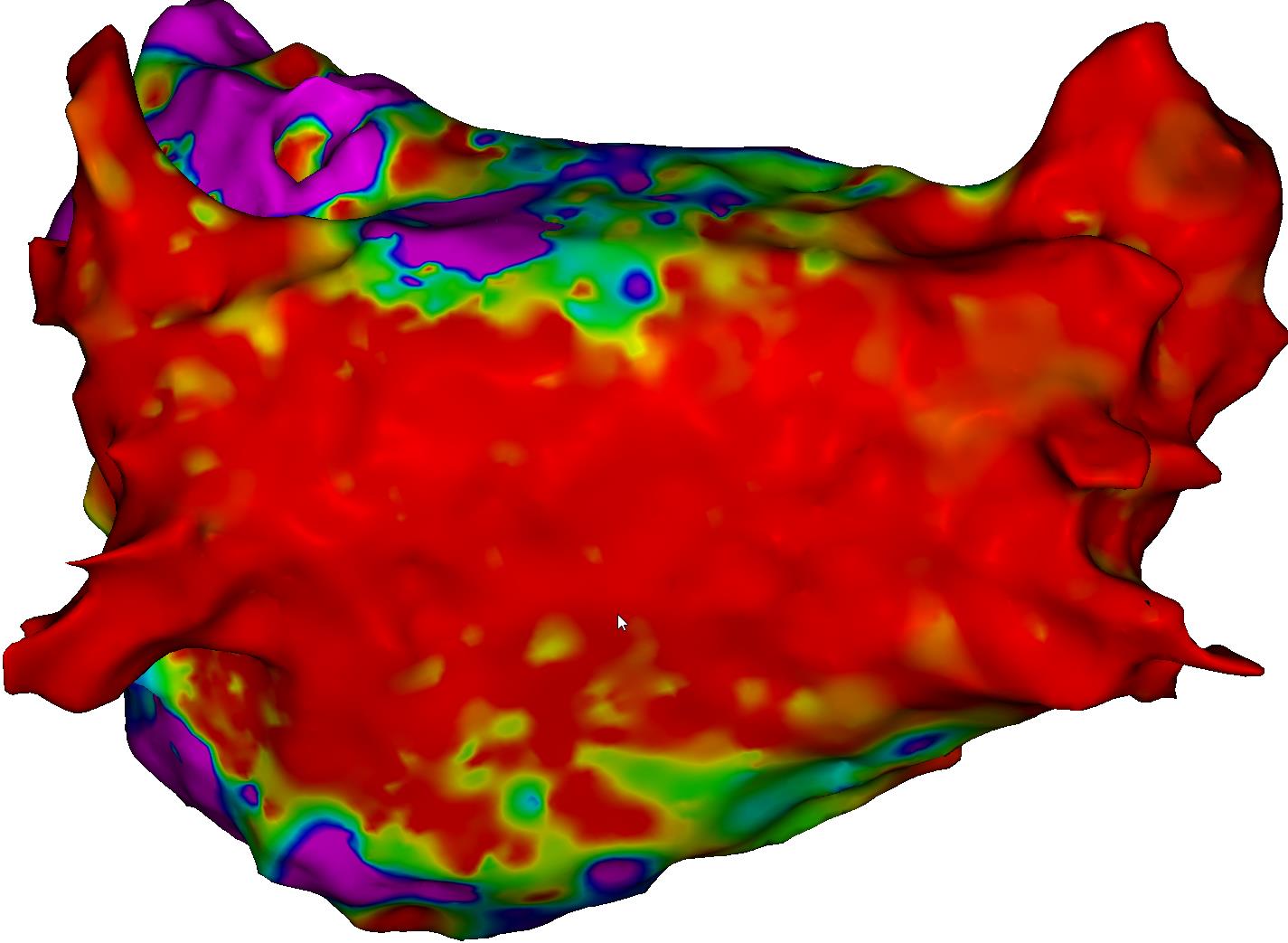 | 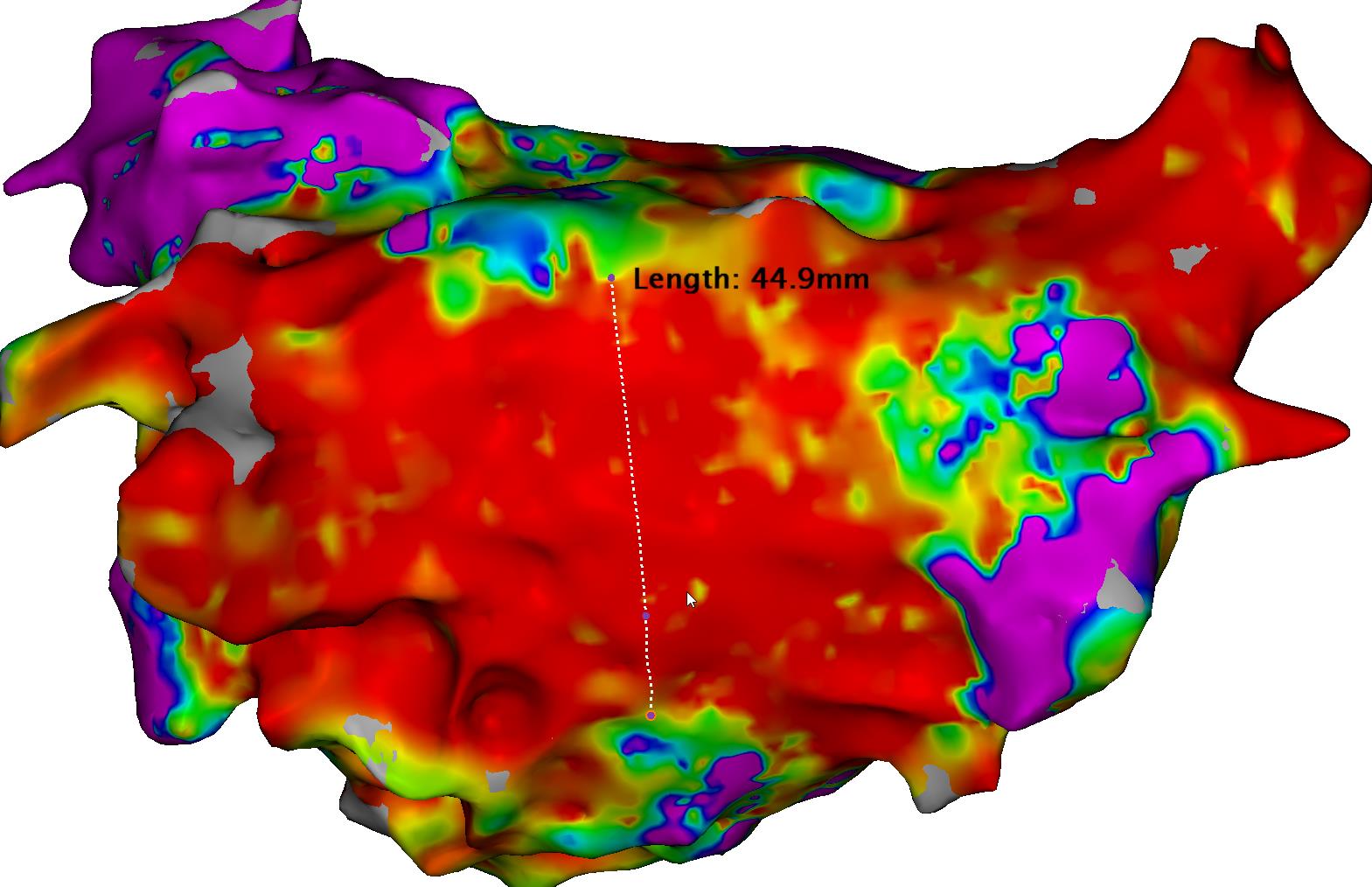 |
| 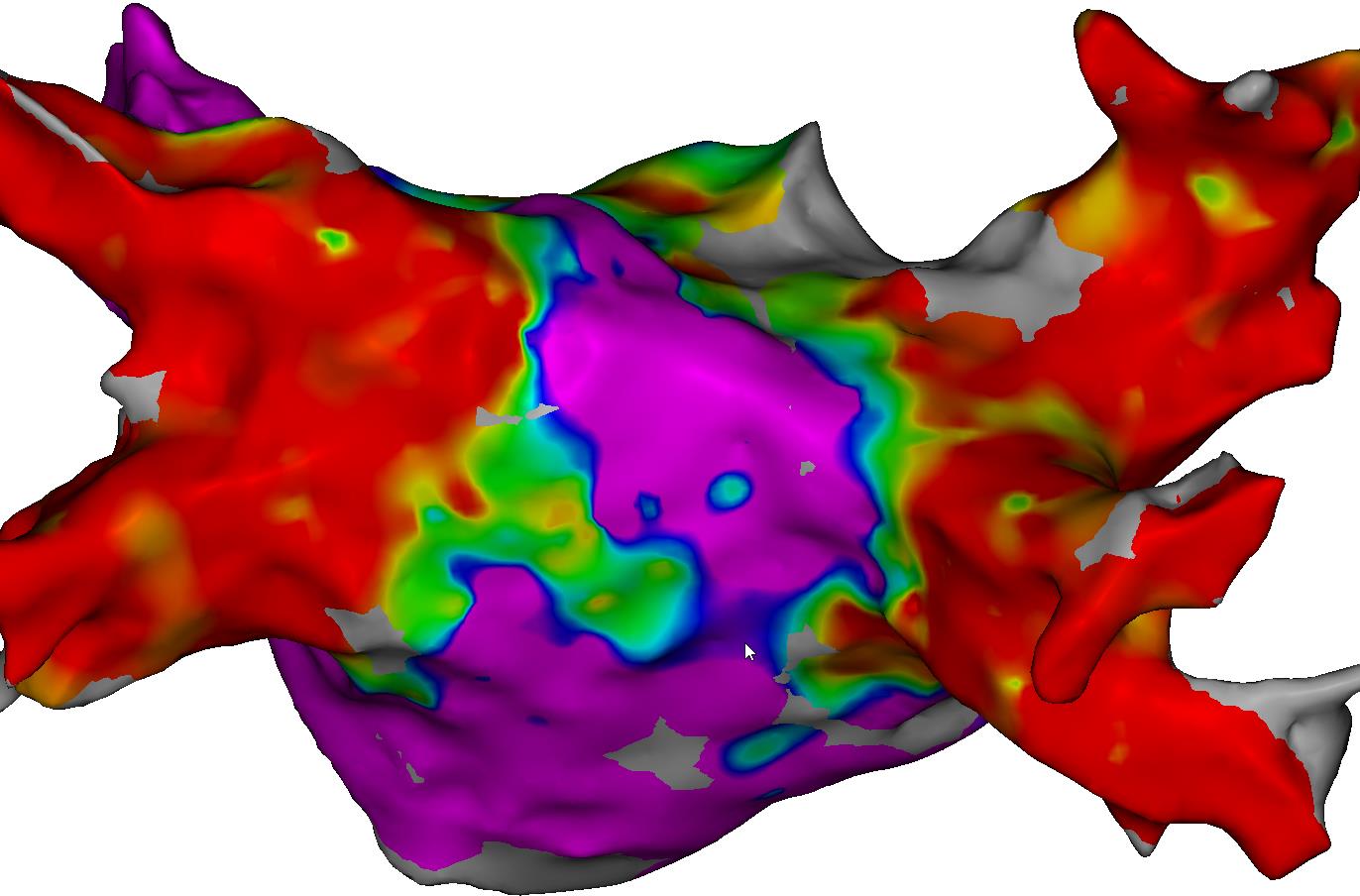 | 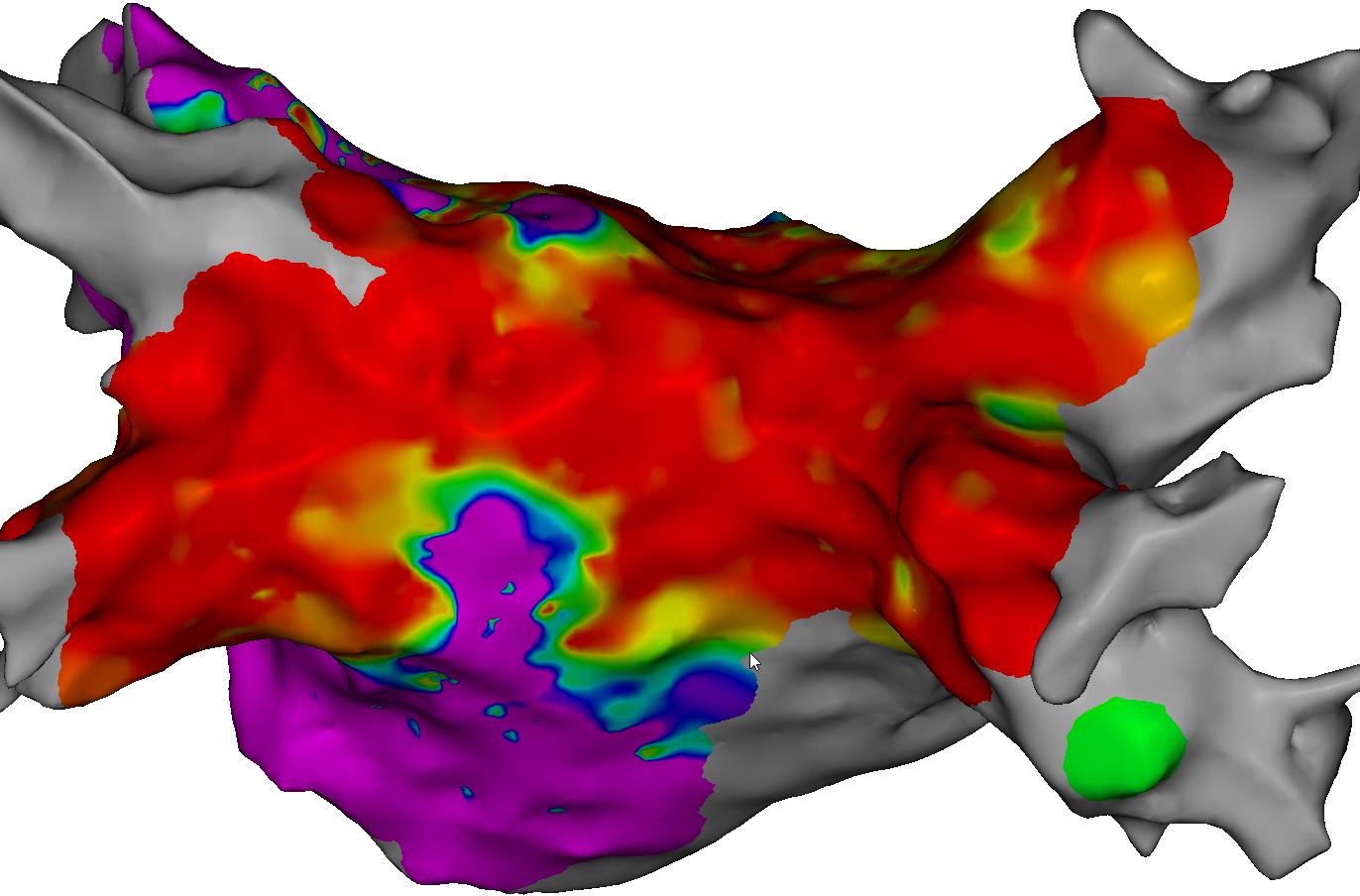 | 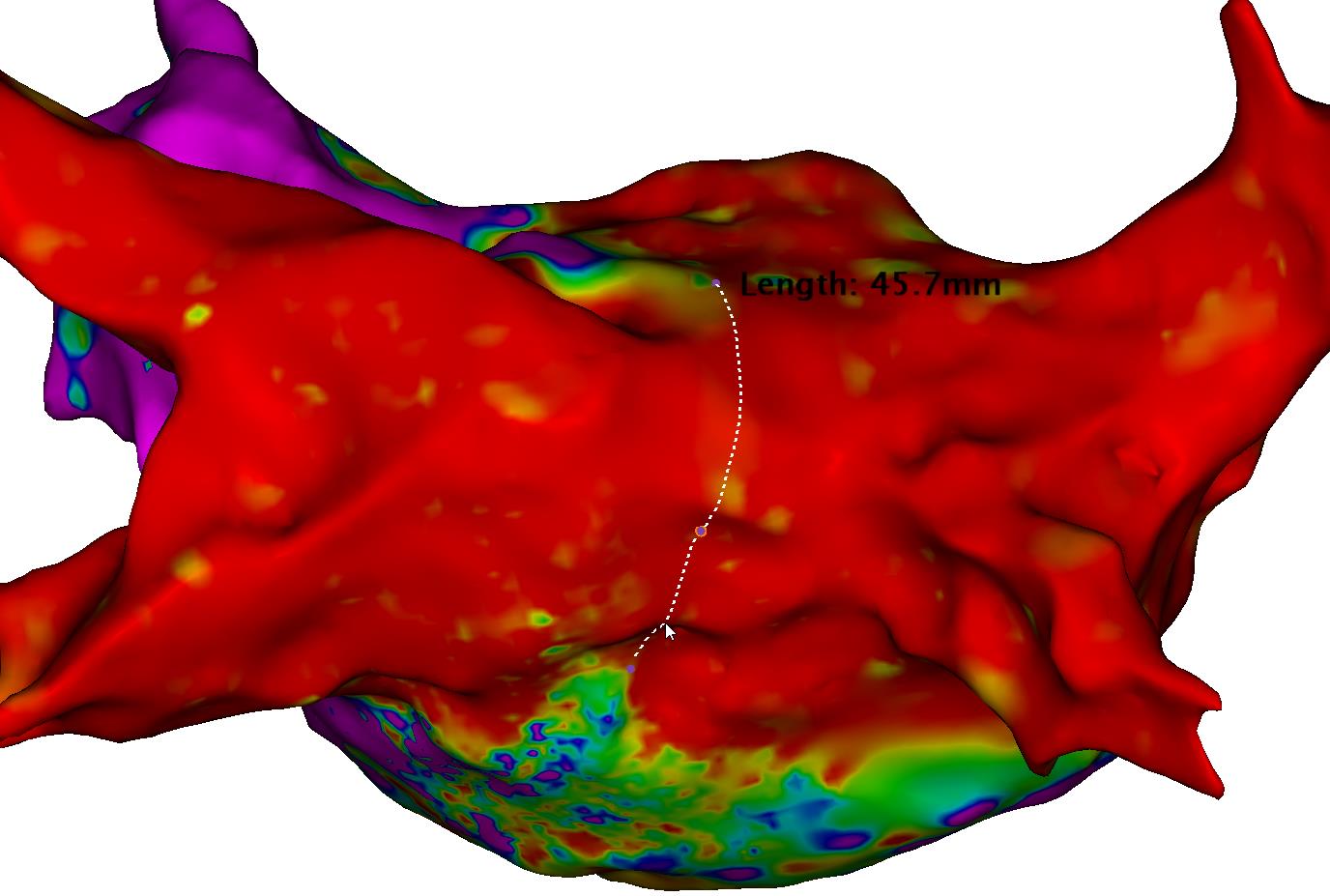 |
| 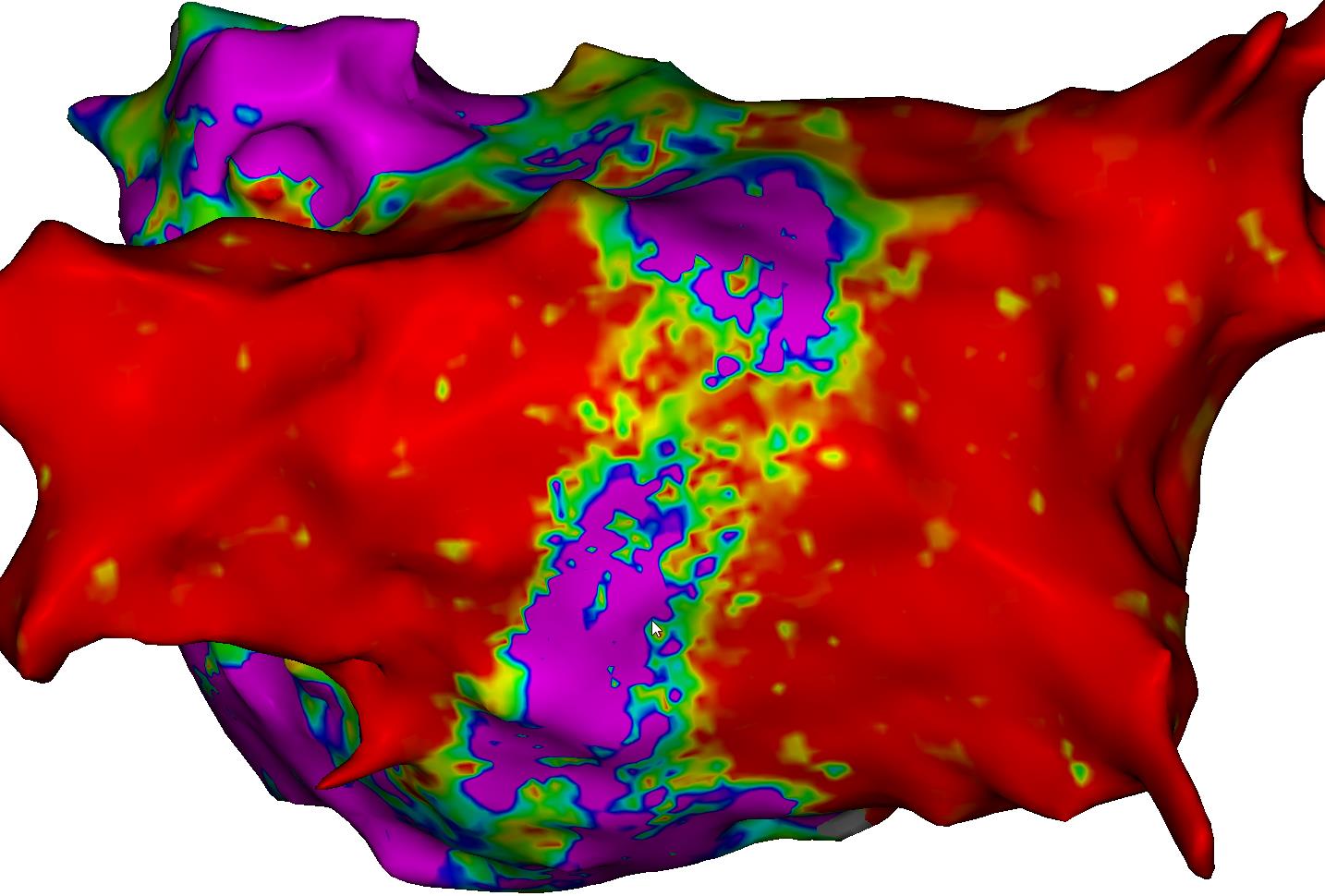 | 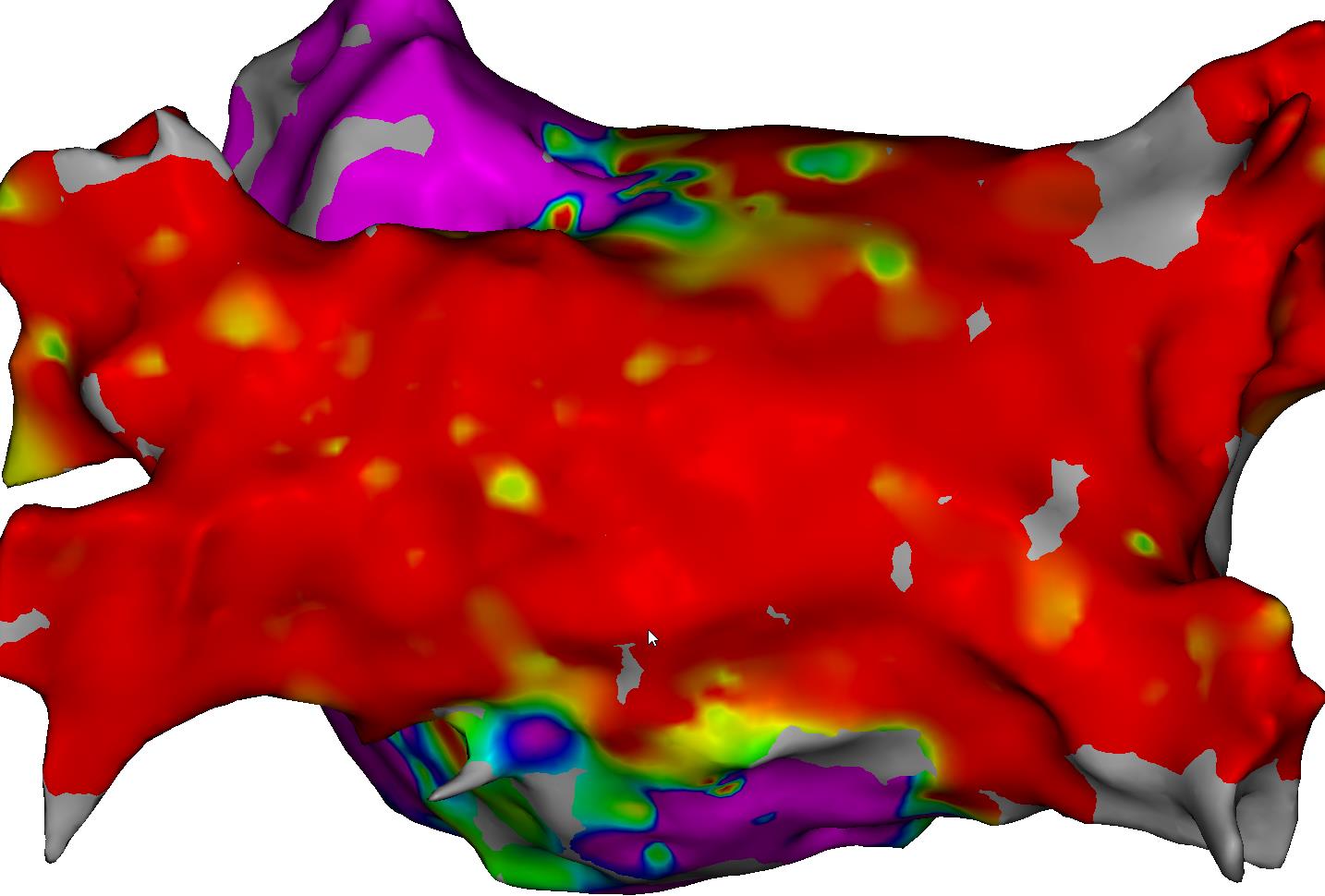 | 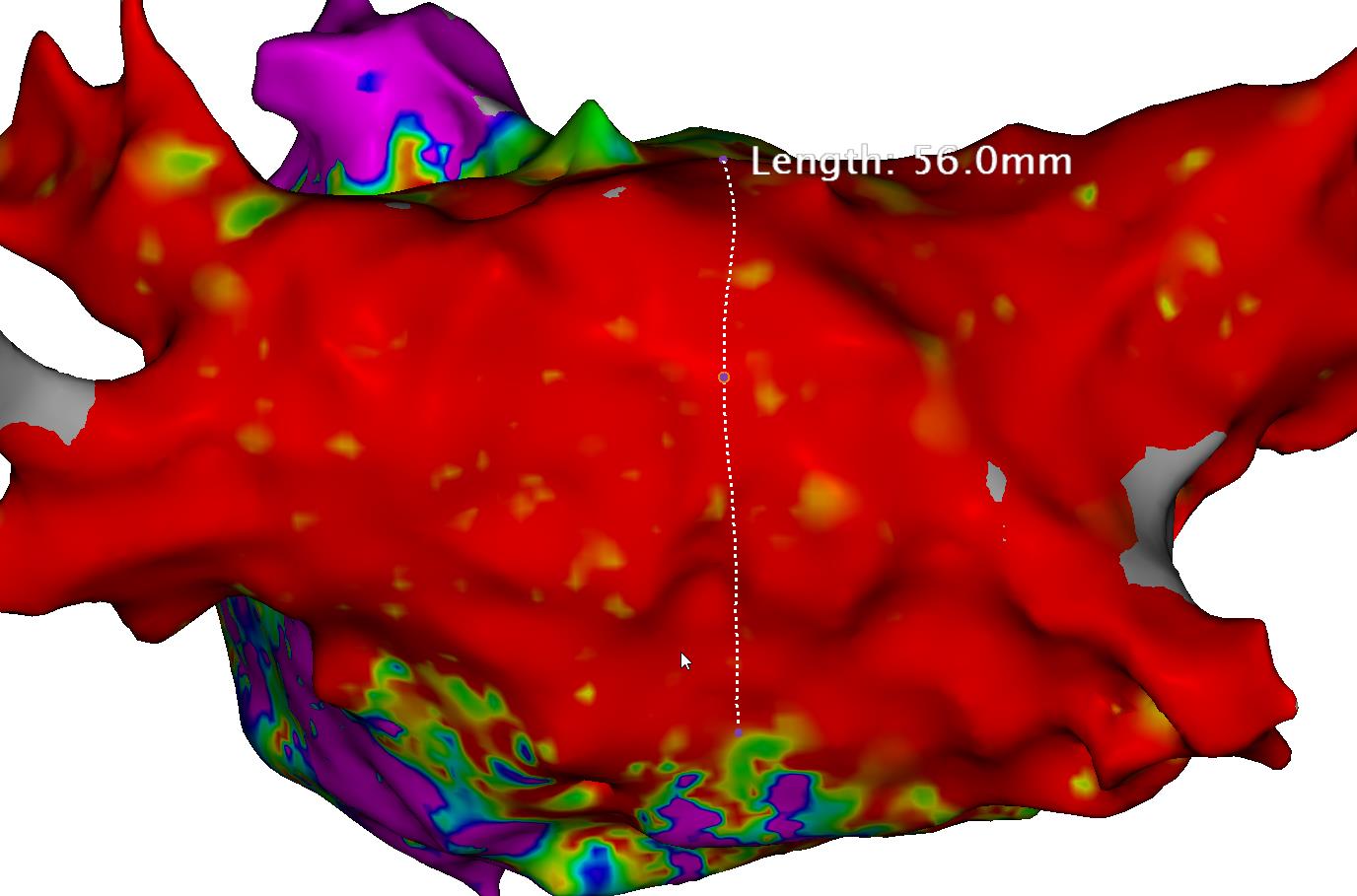 |
| 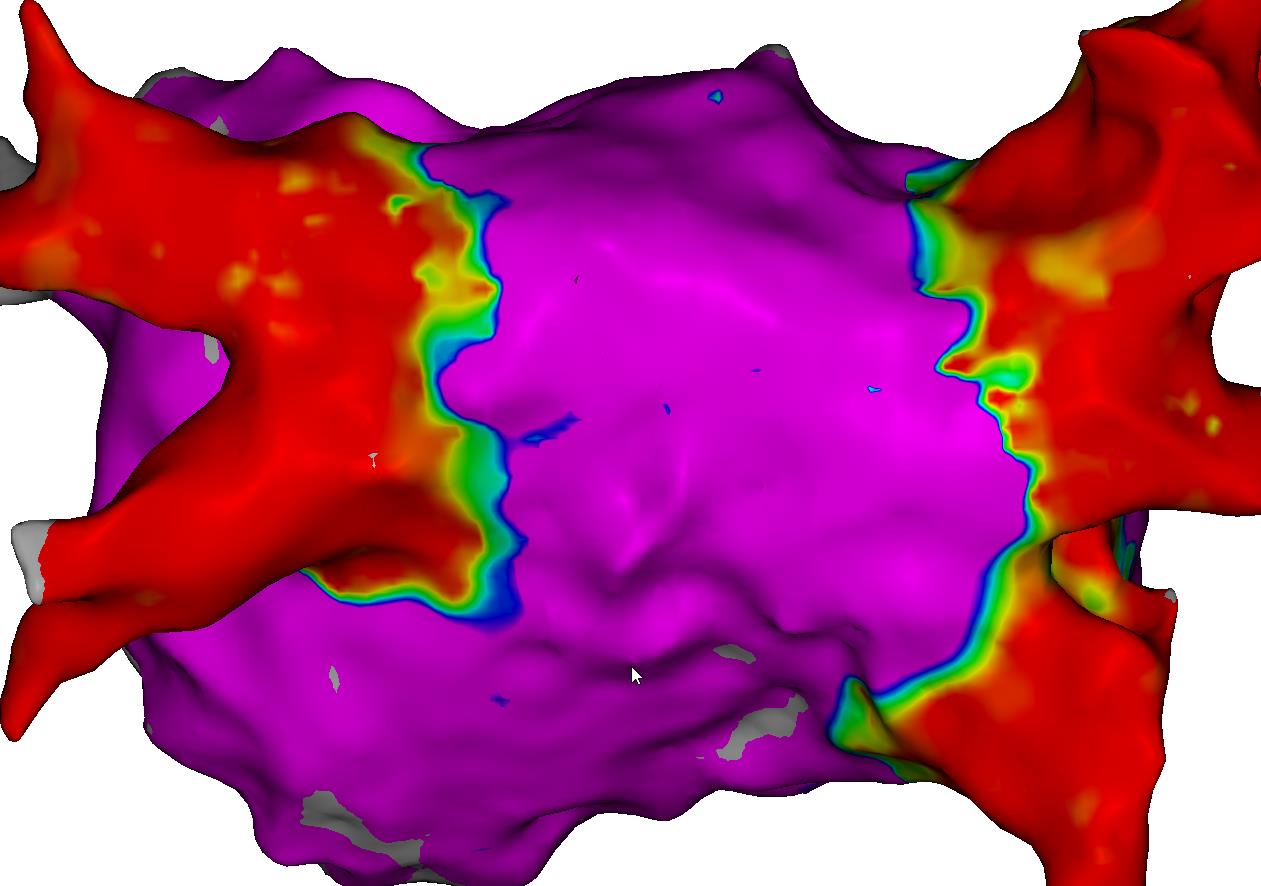 | 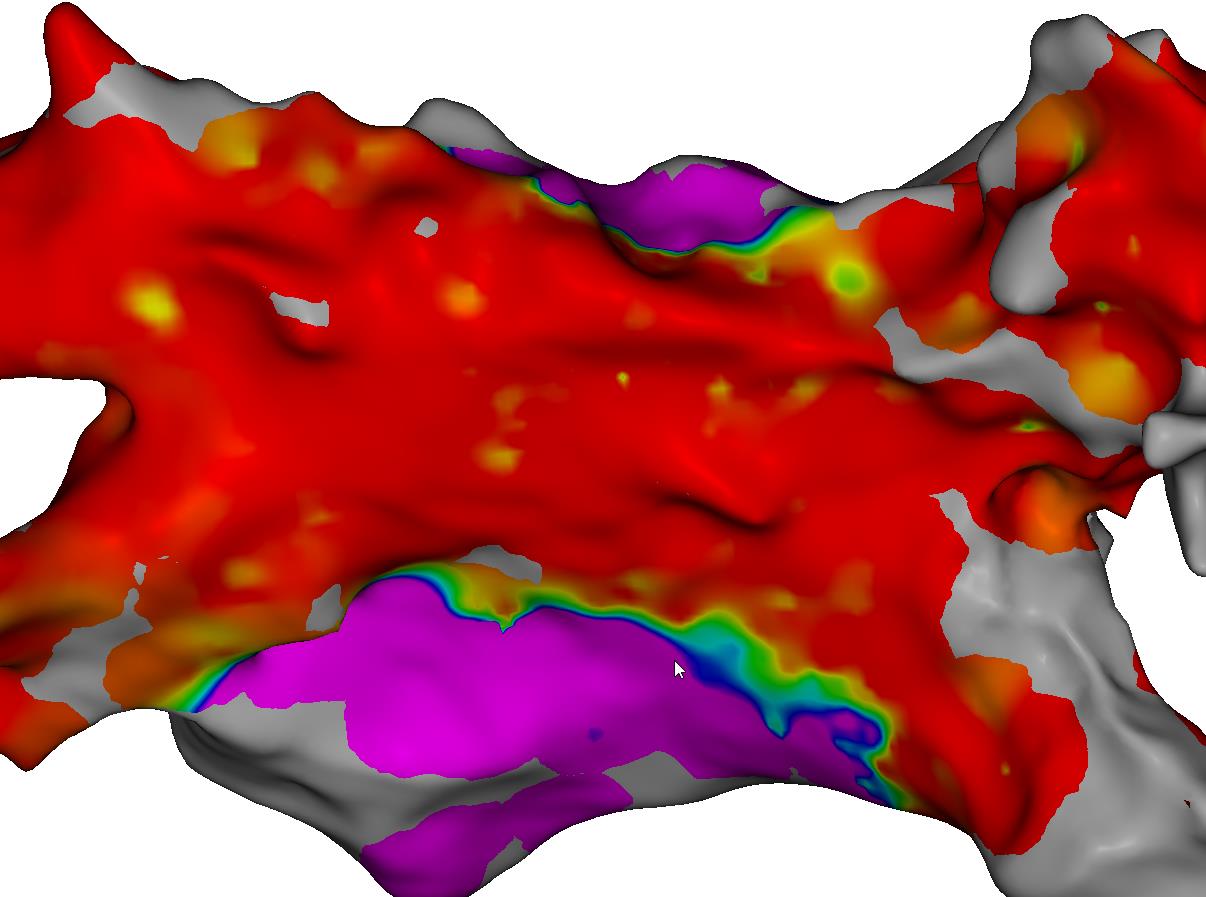 | 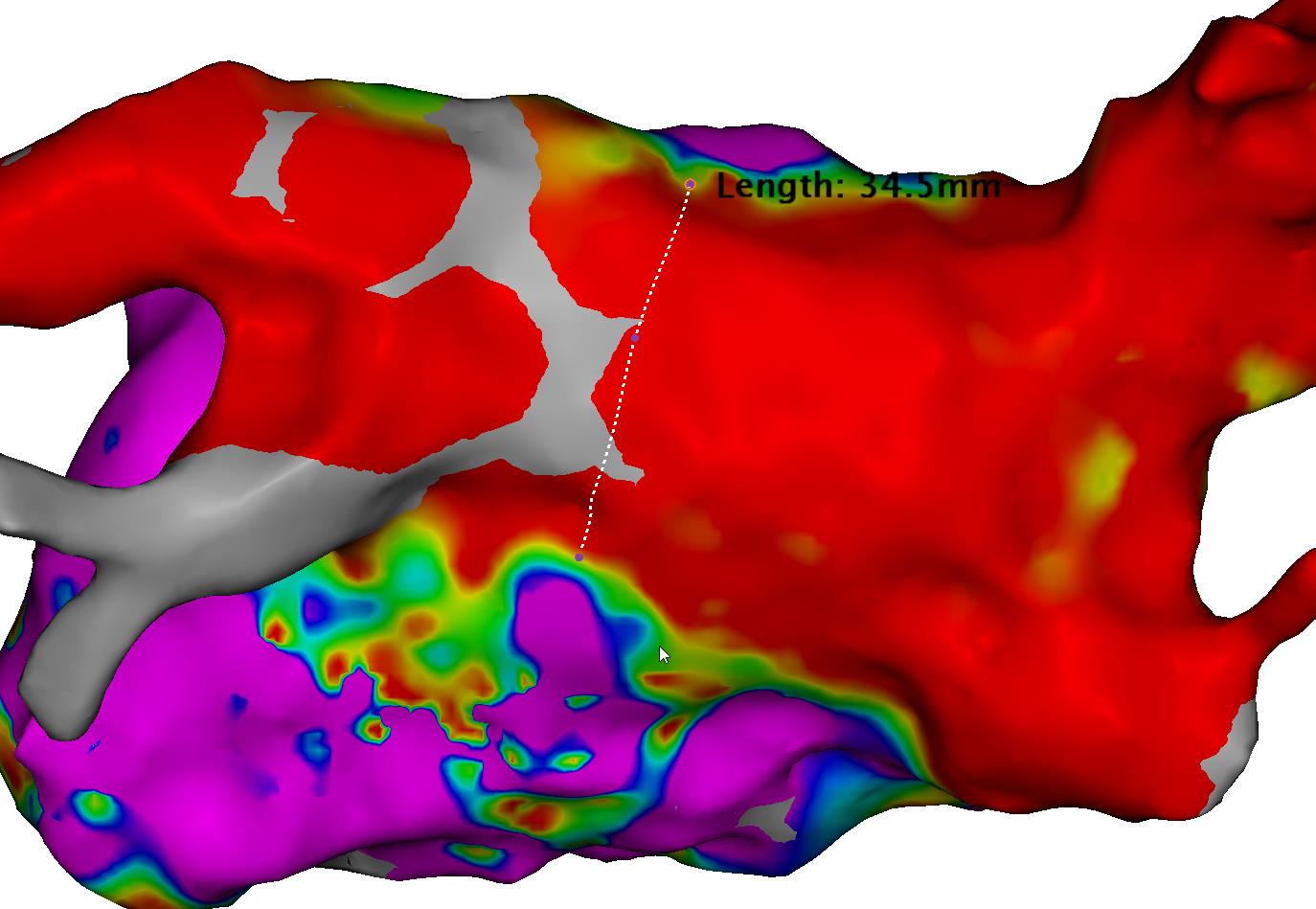 |
| 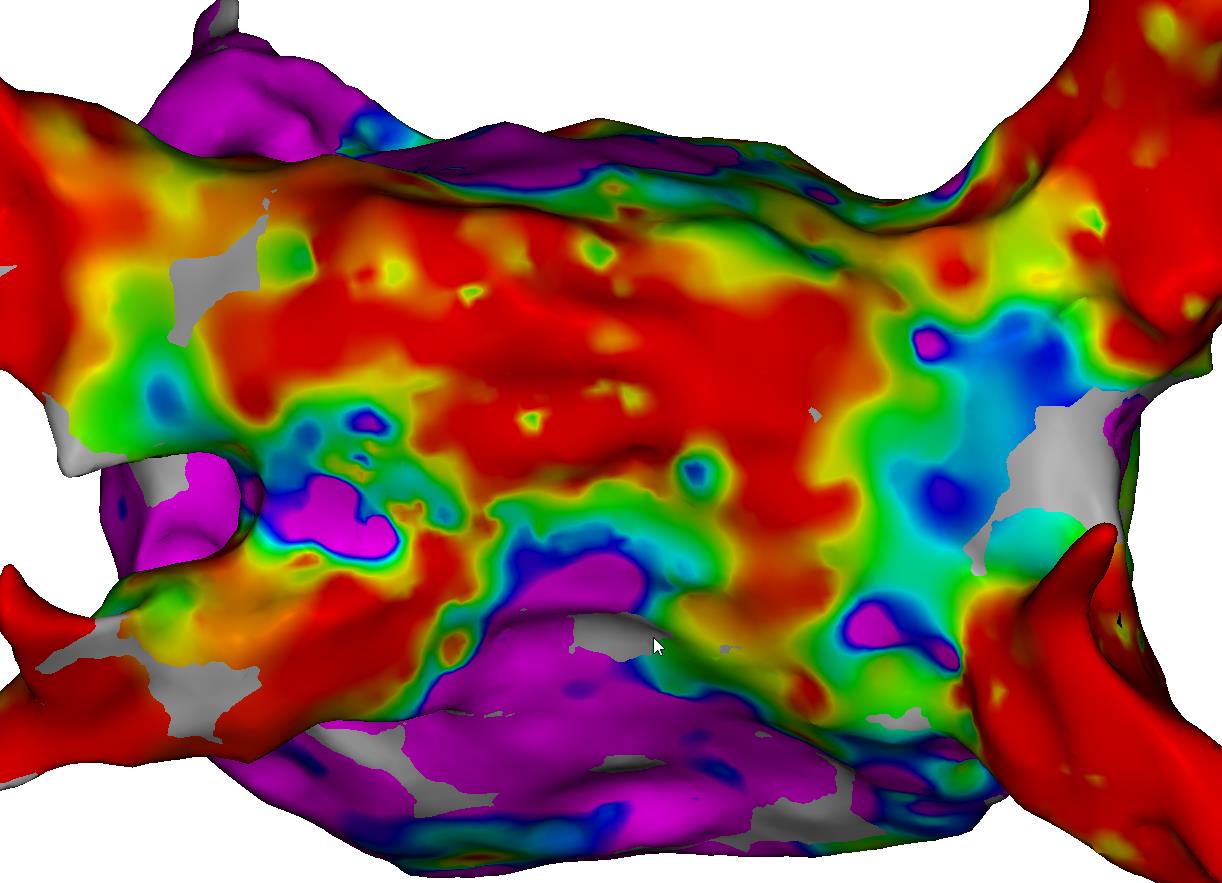 | 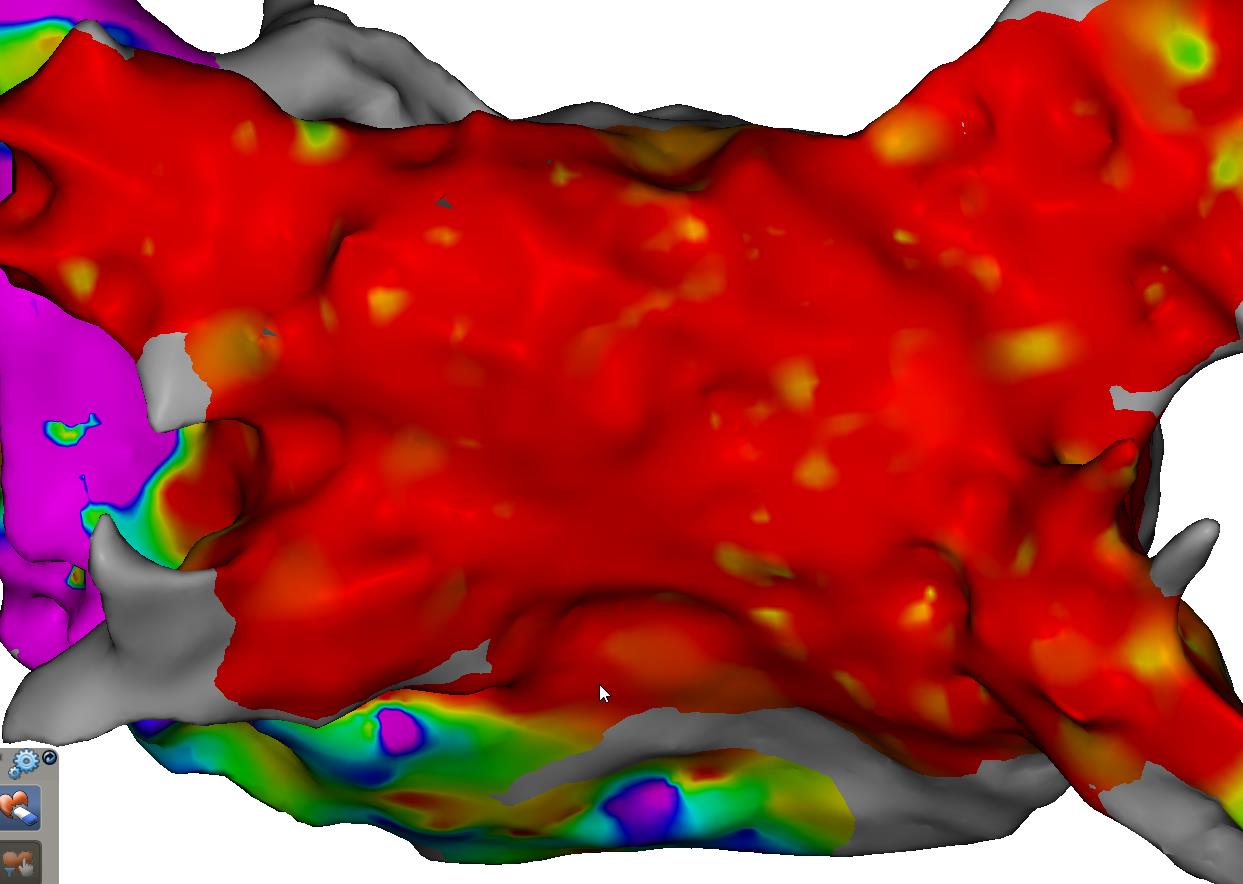 | 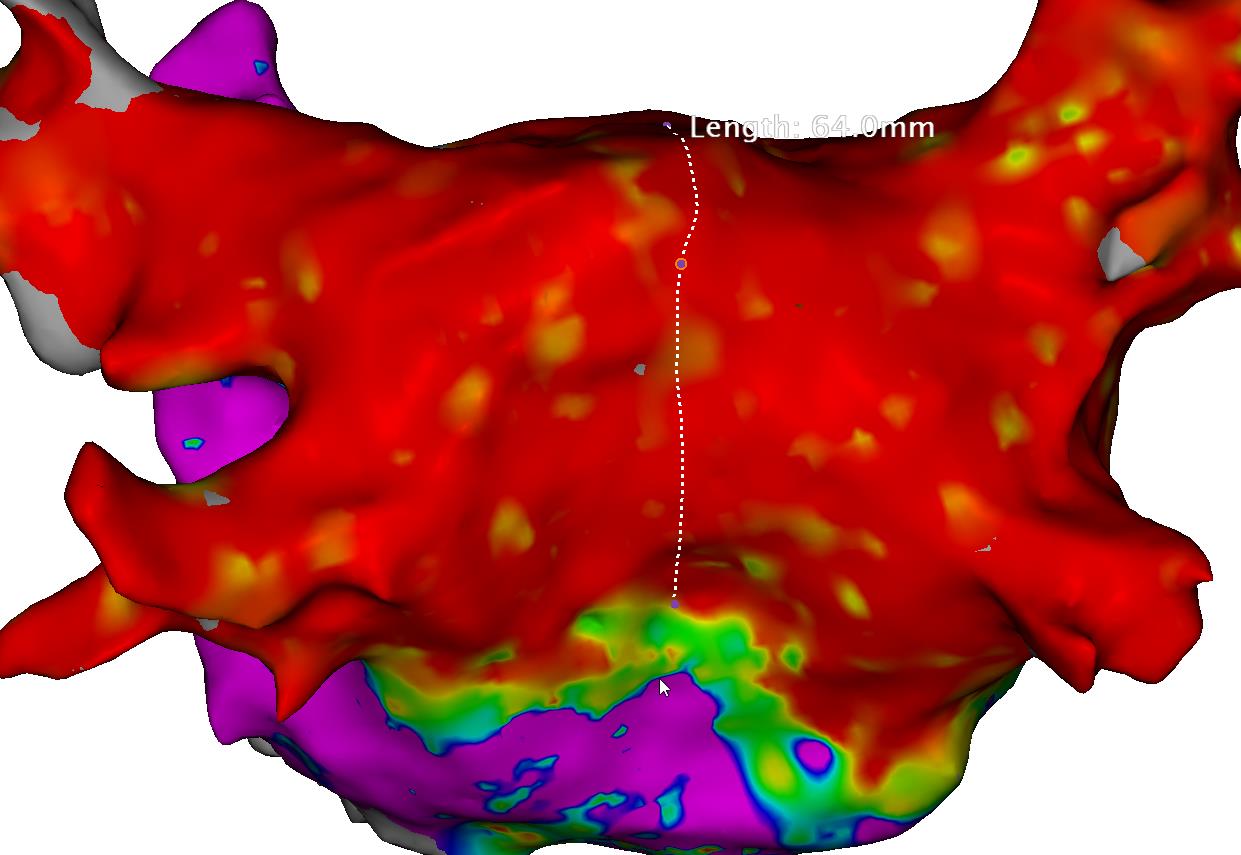 |
| 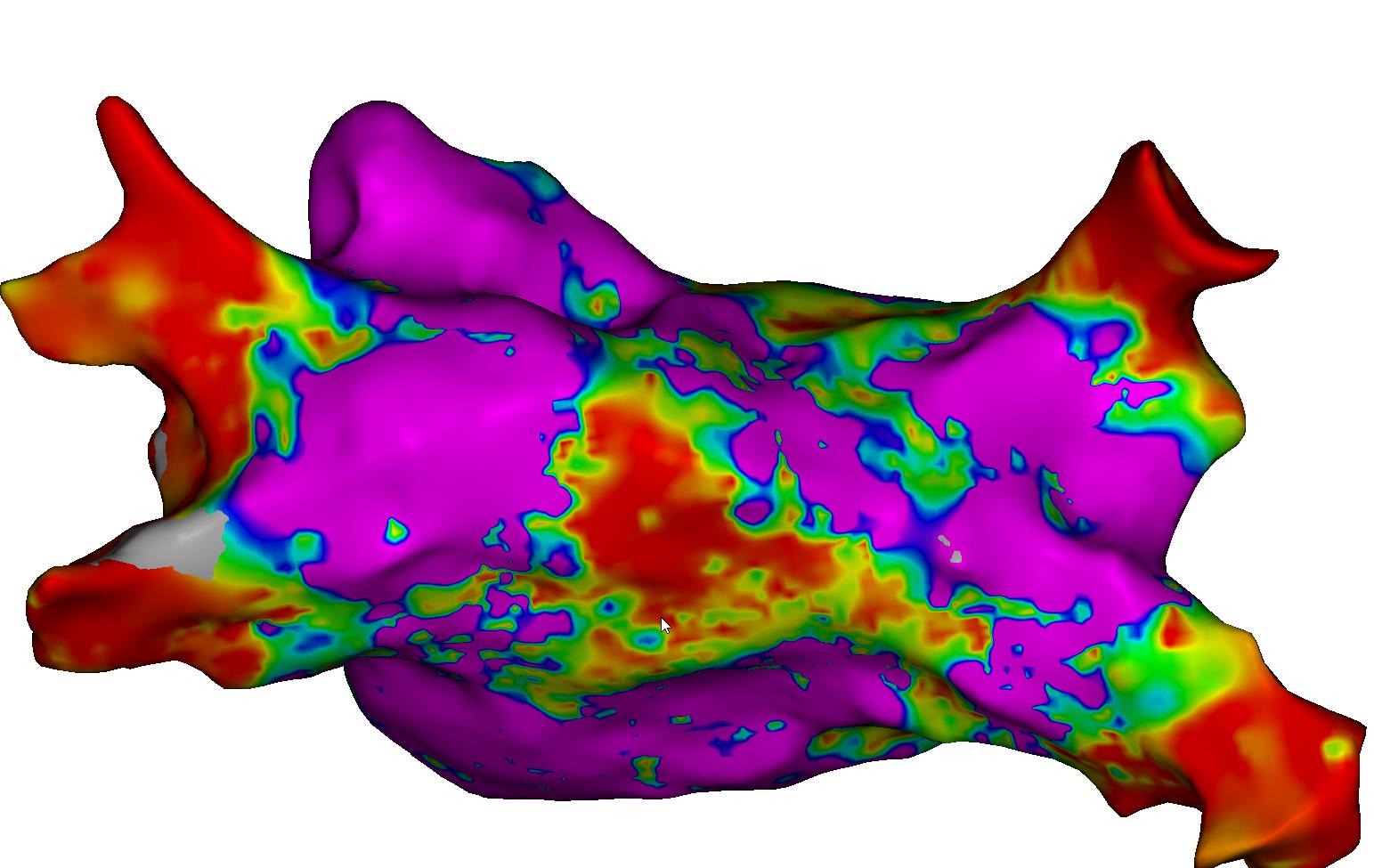 | 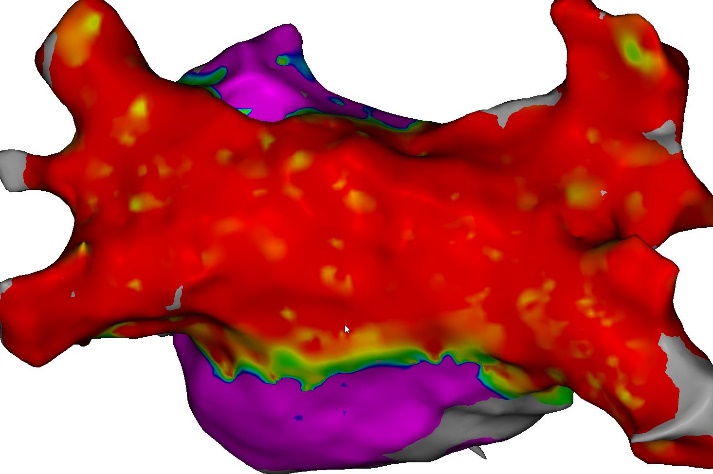 | 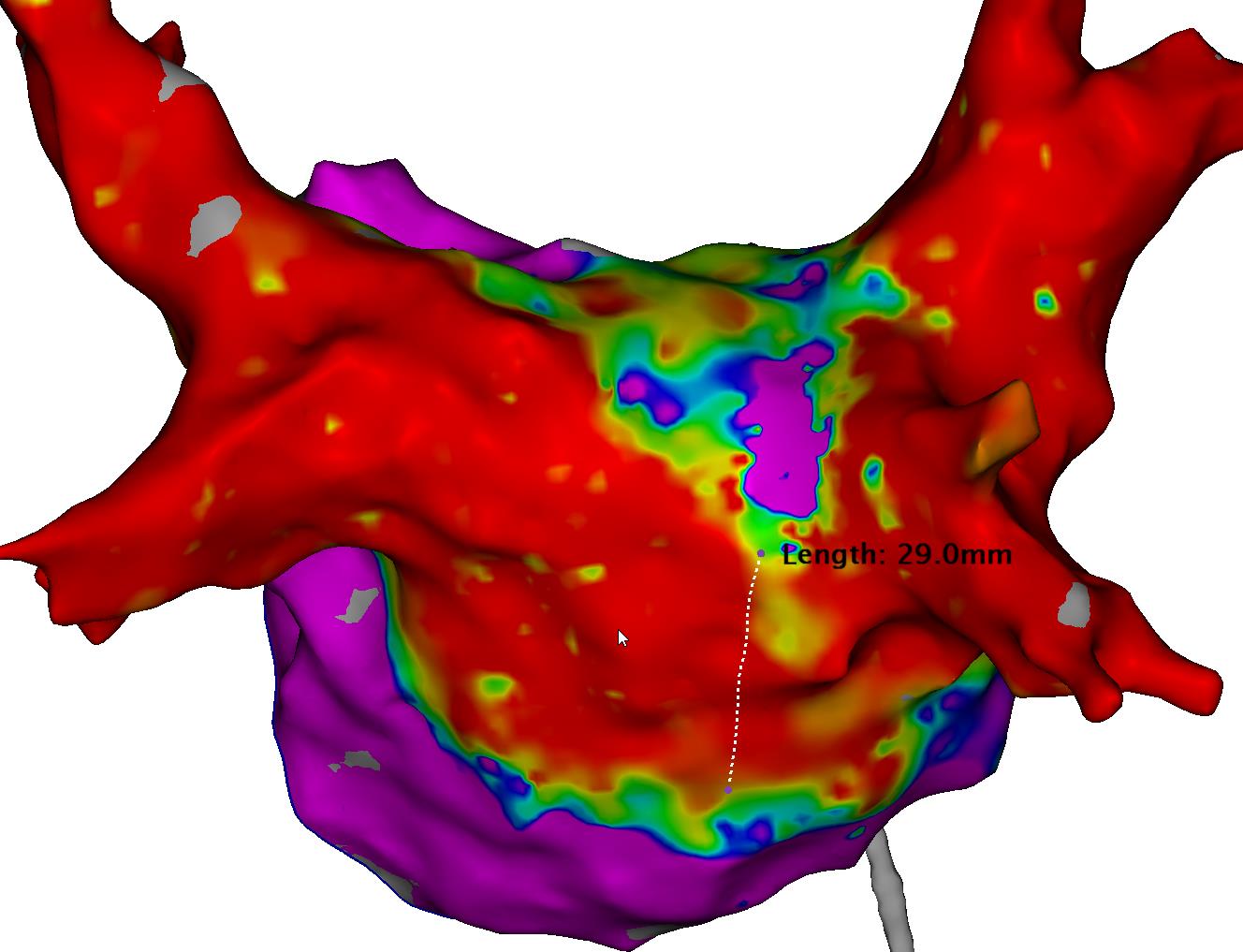 Regression but no reconnection |
| 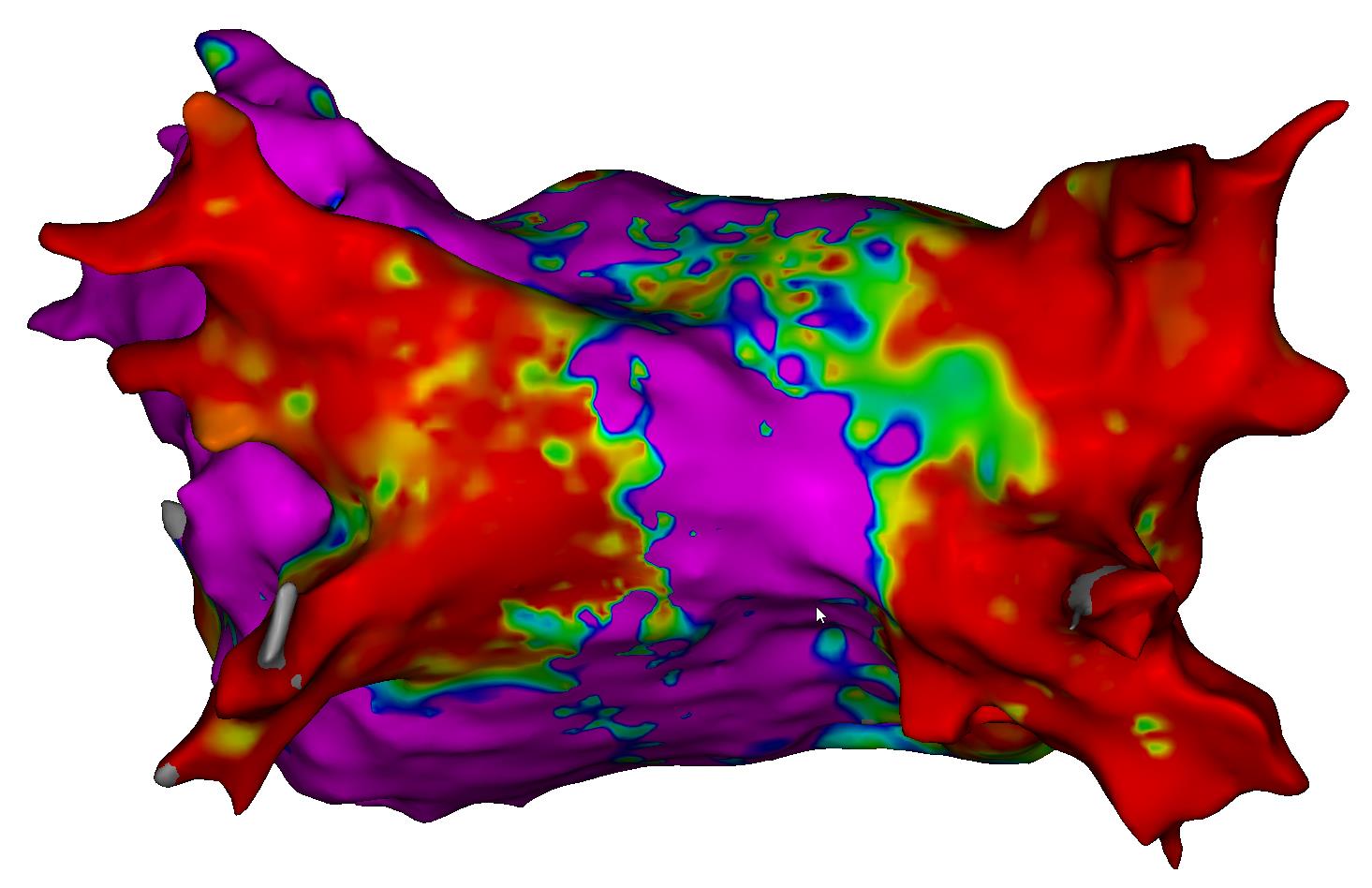 | 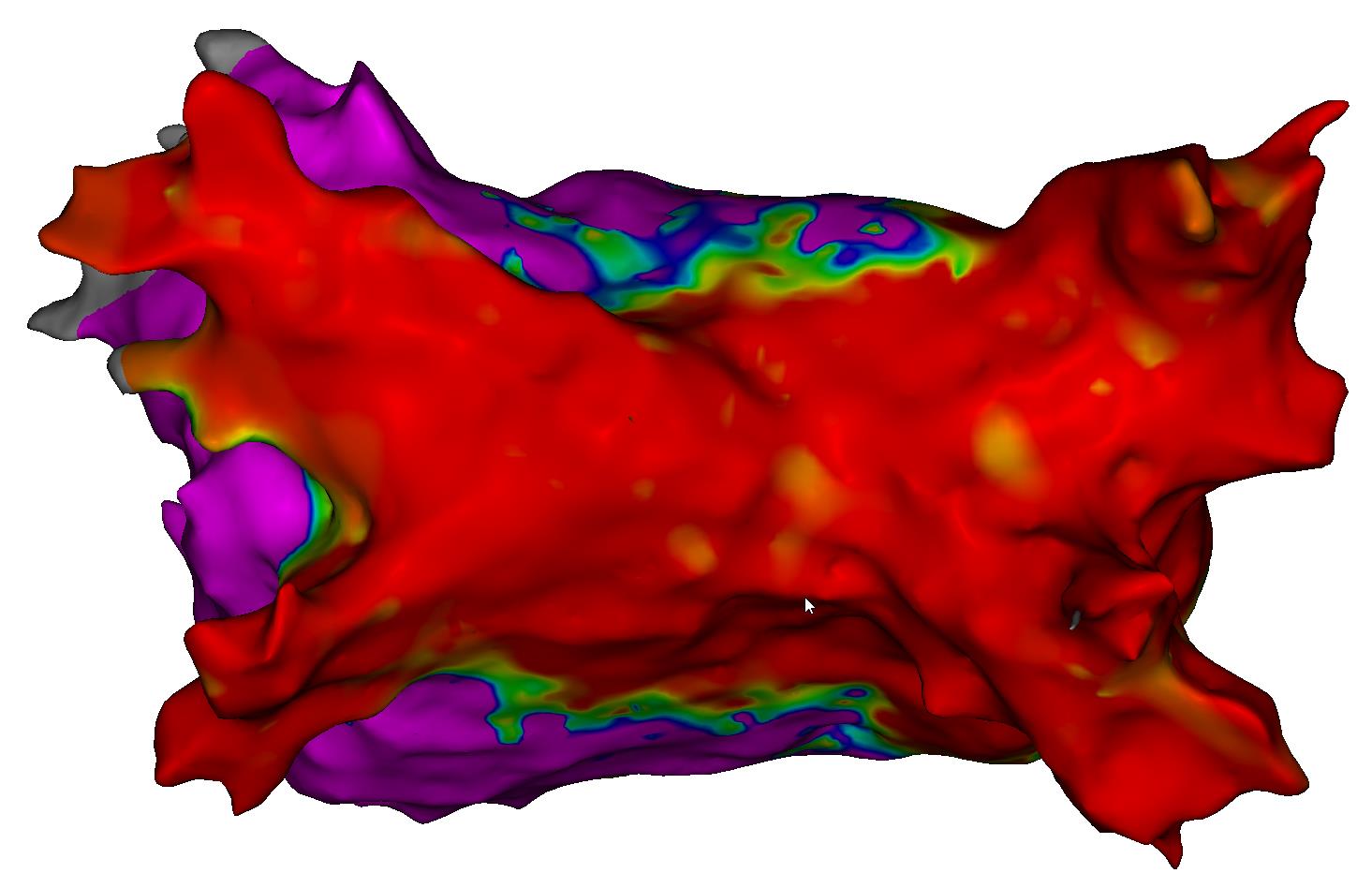 | 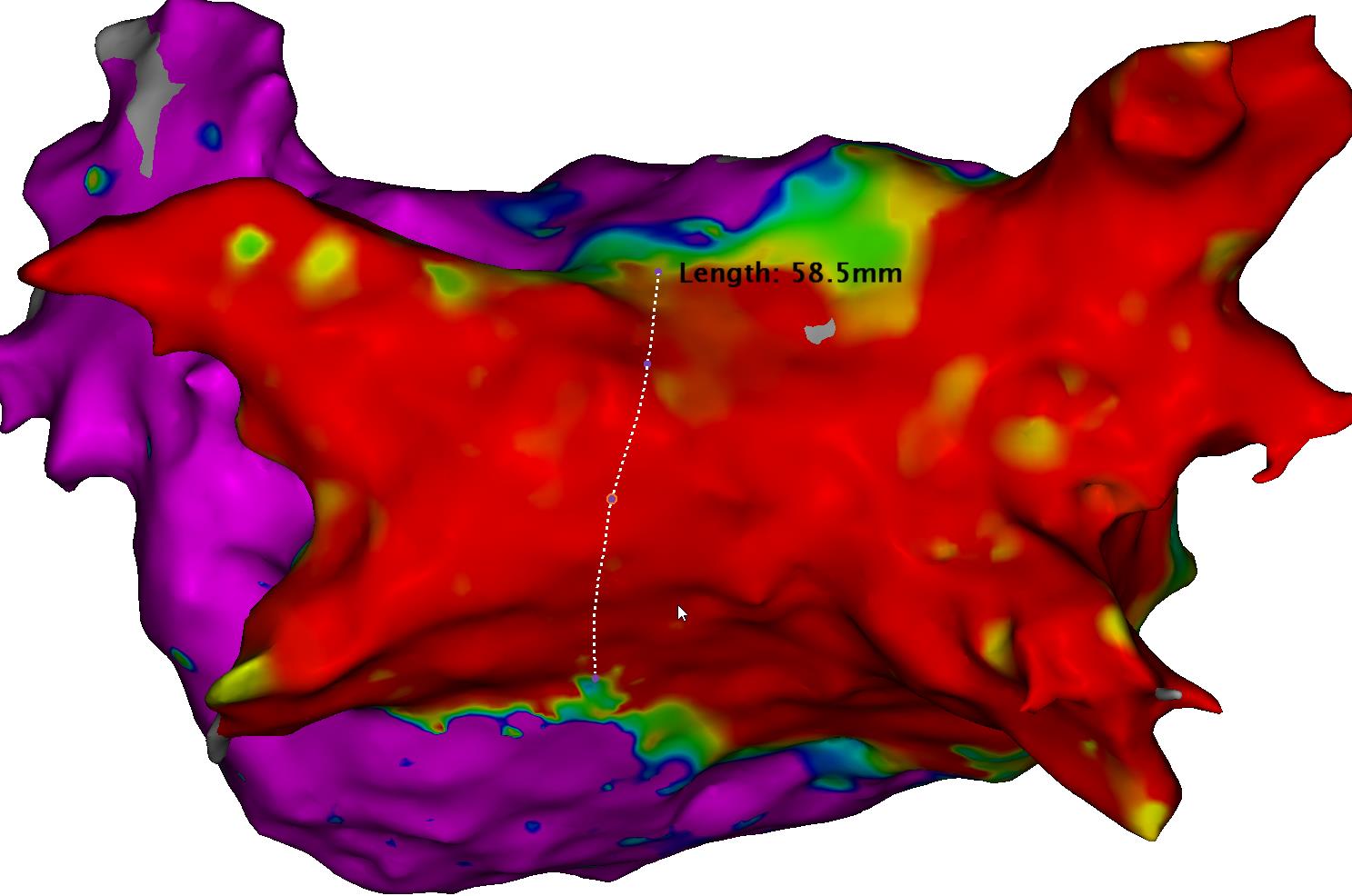 |
| No map acquired | 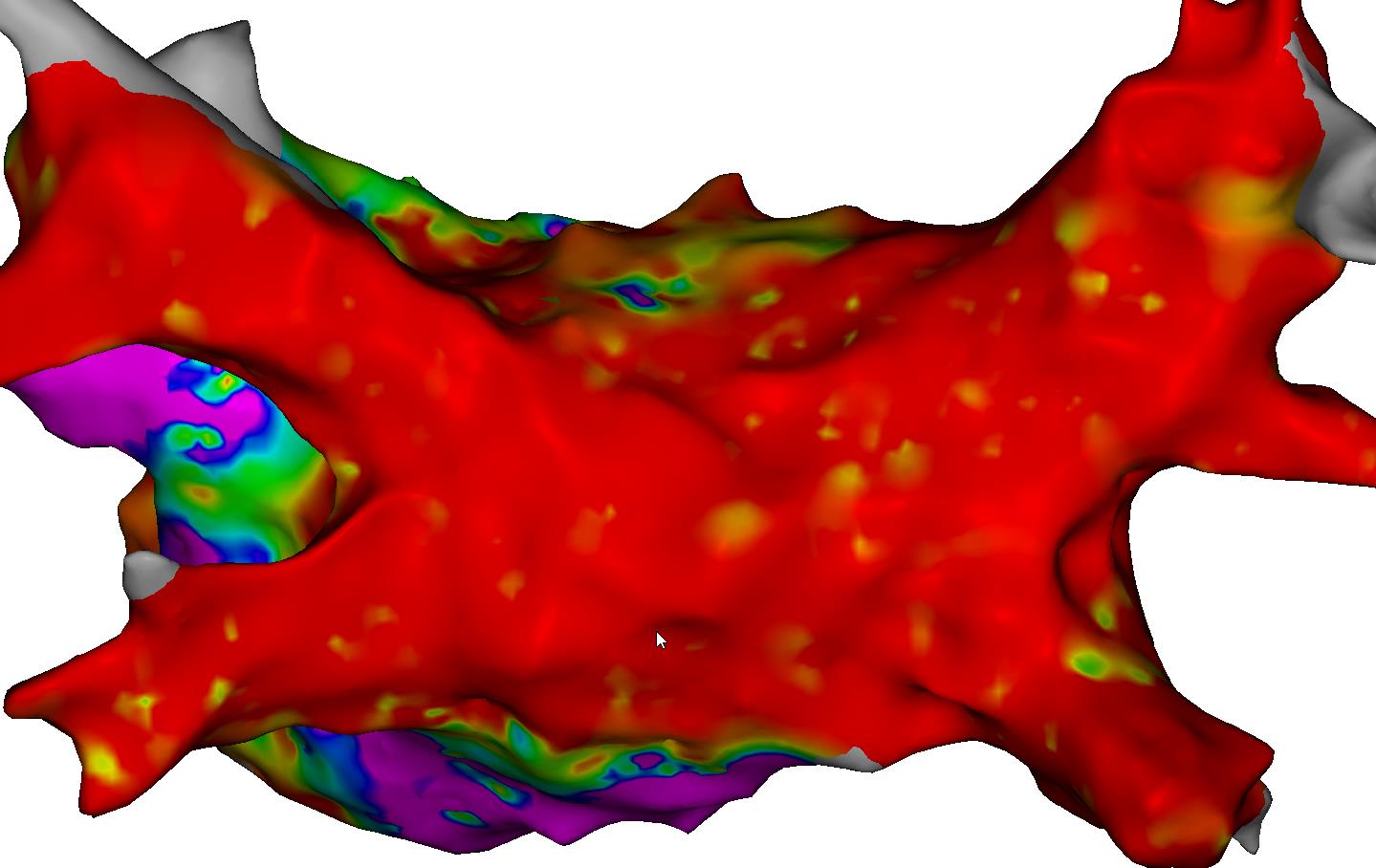 | 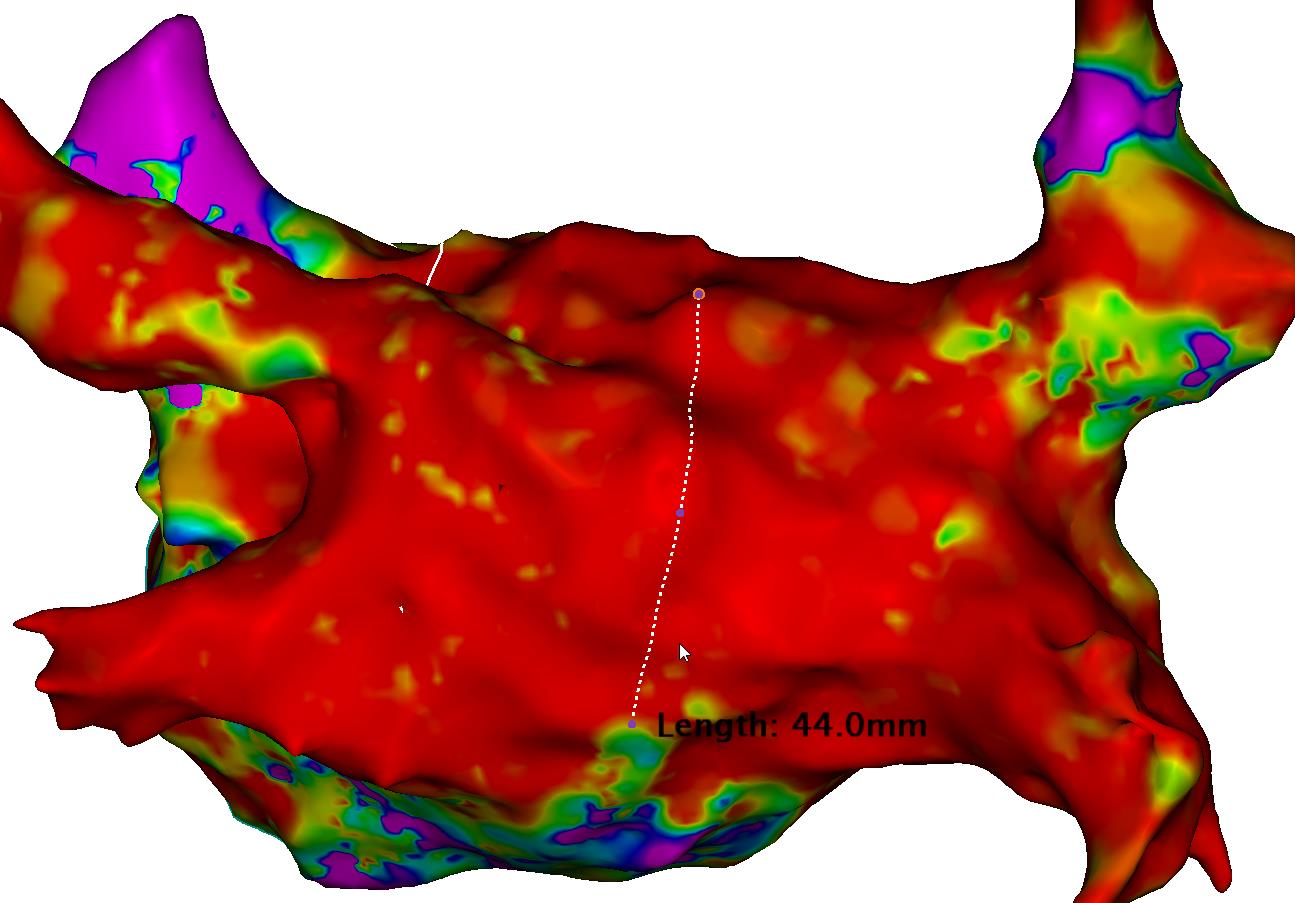 |
| 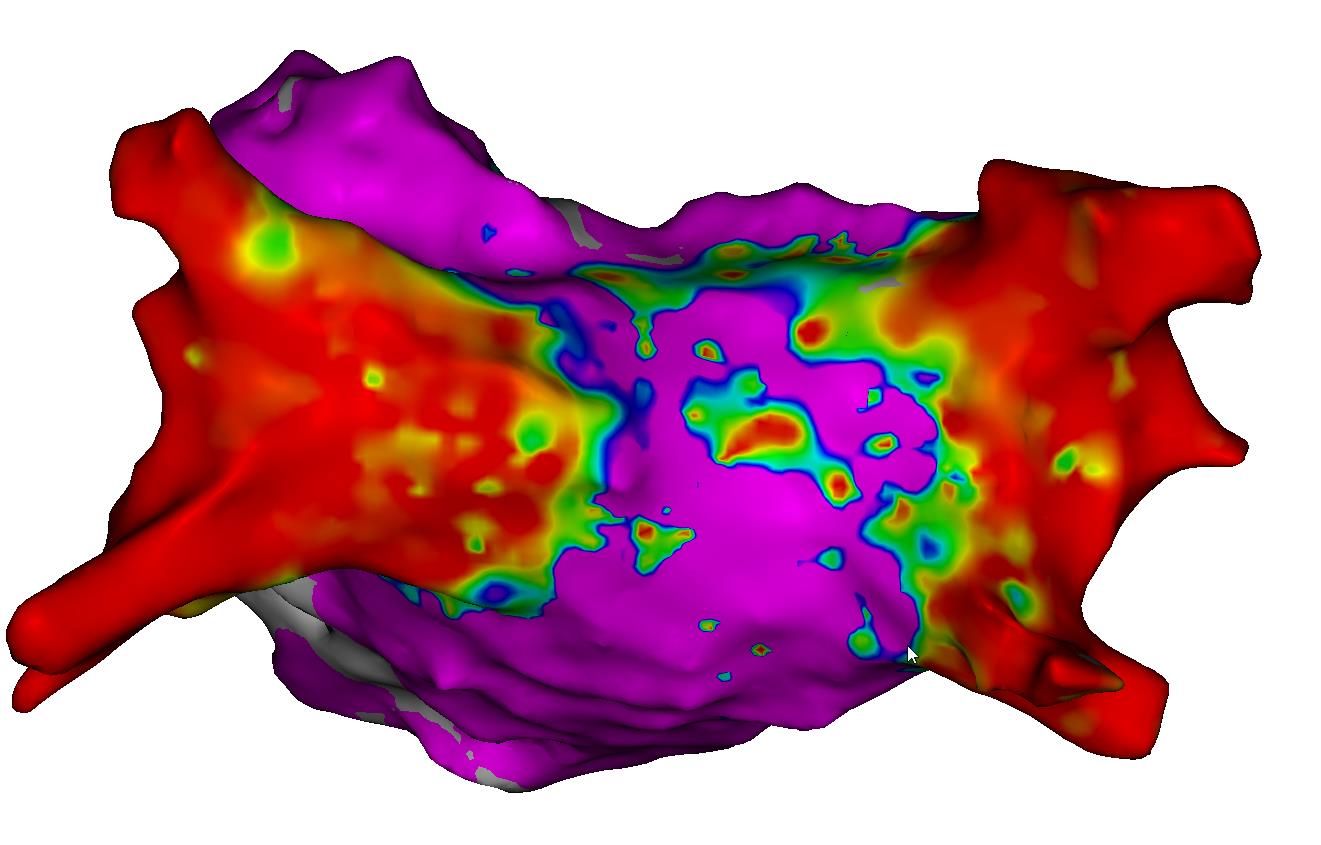 | 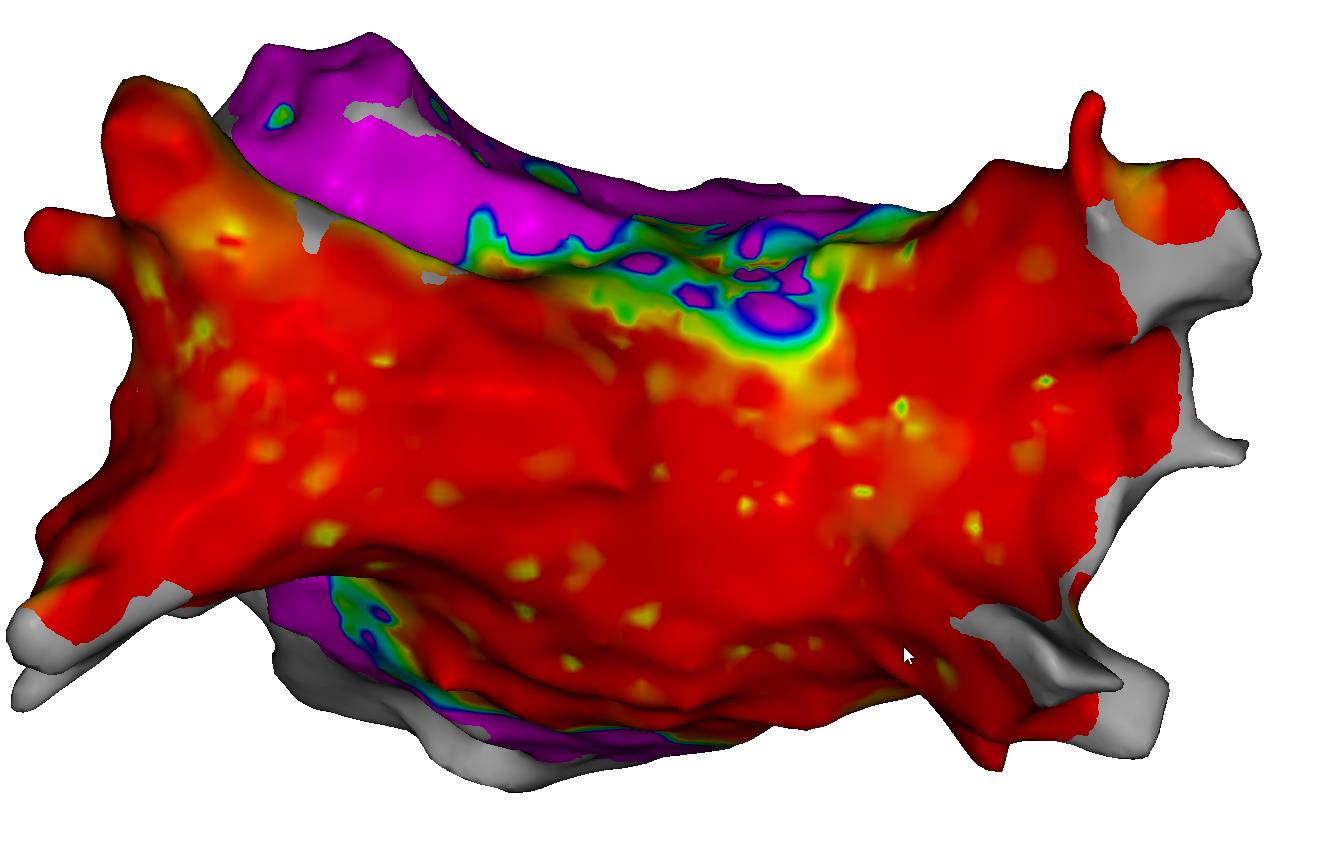 | Regression but no reconnection 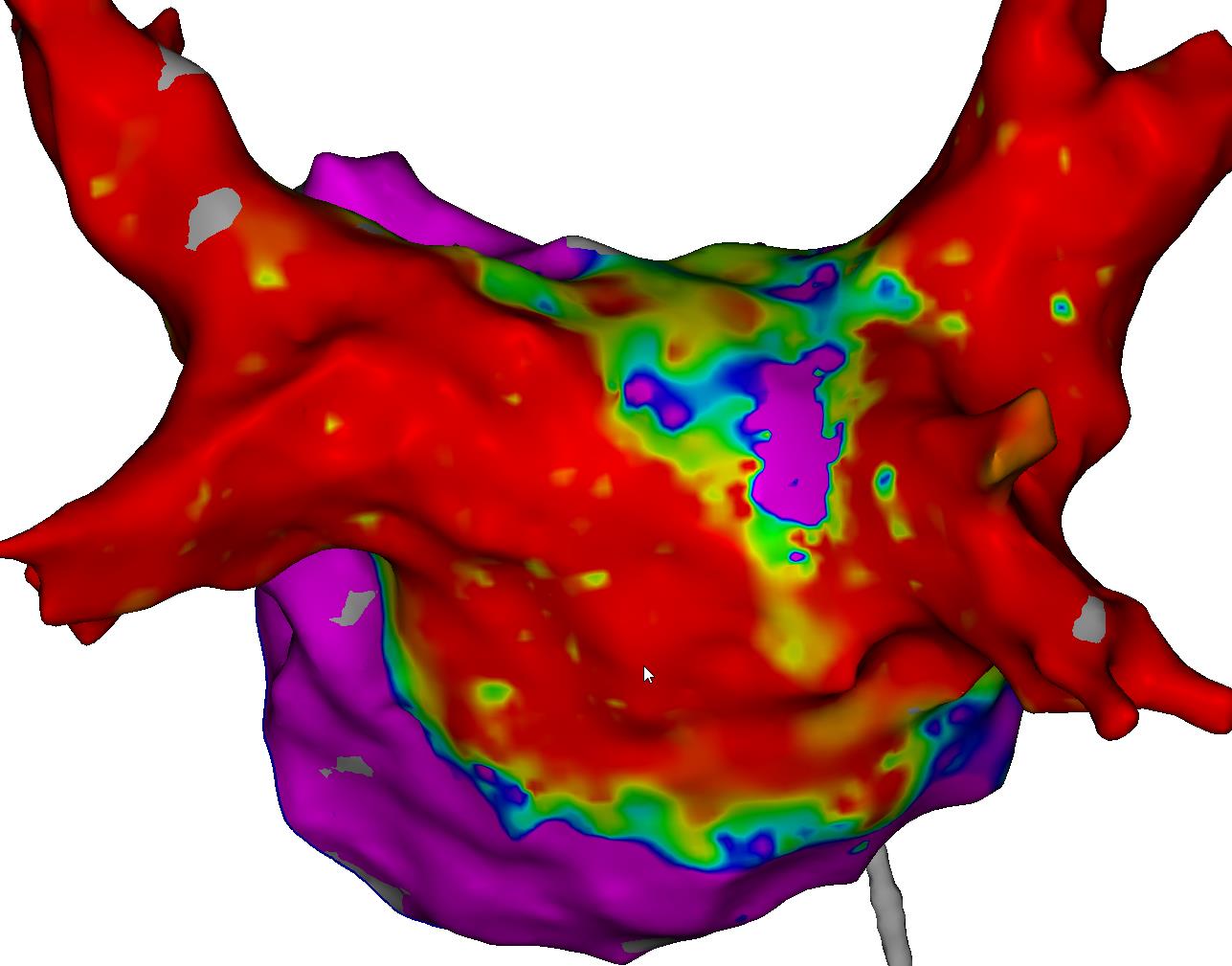 |
| 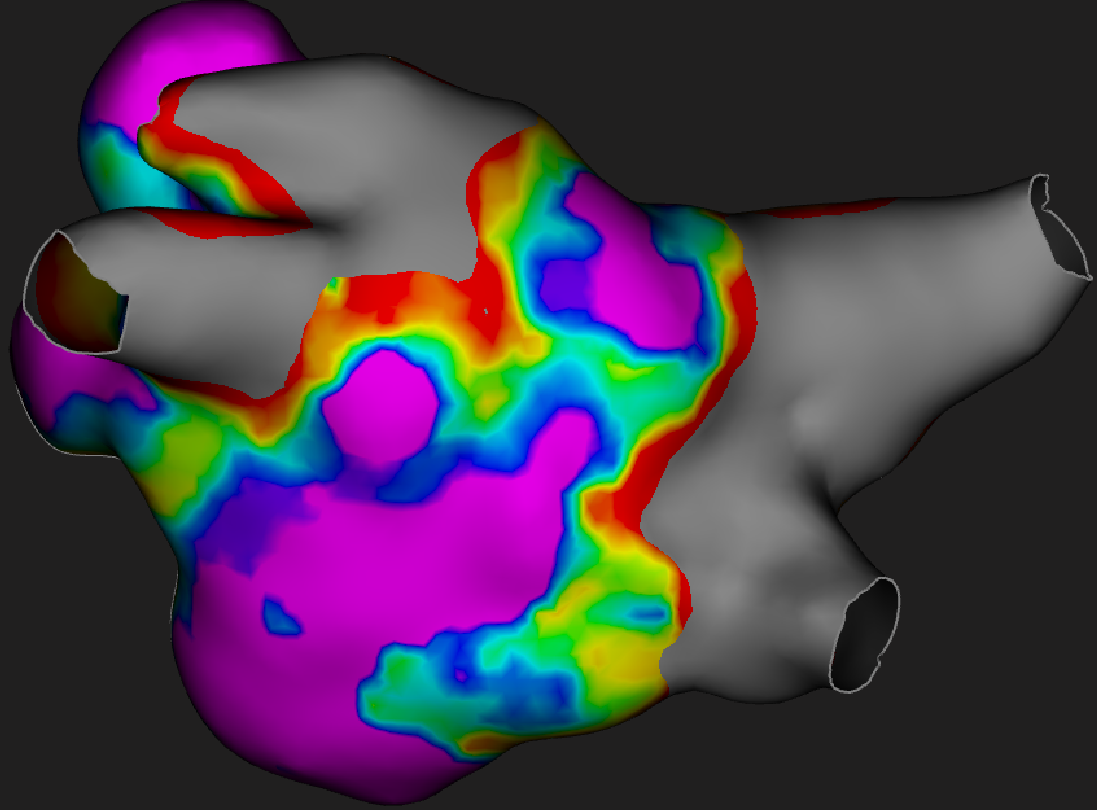 | 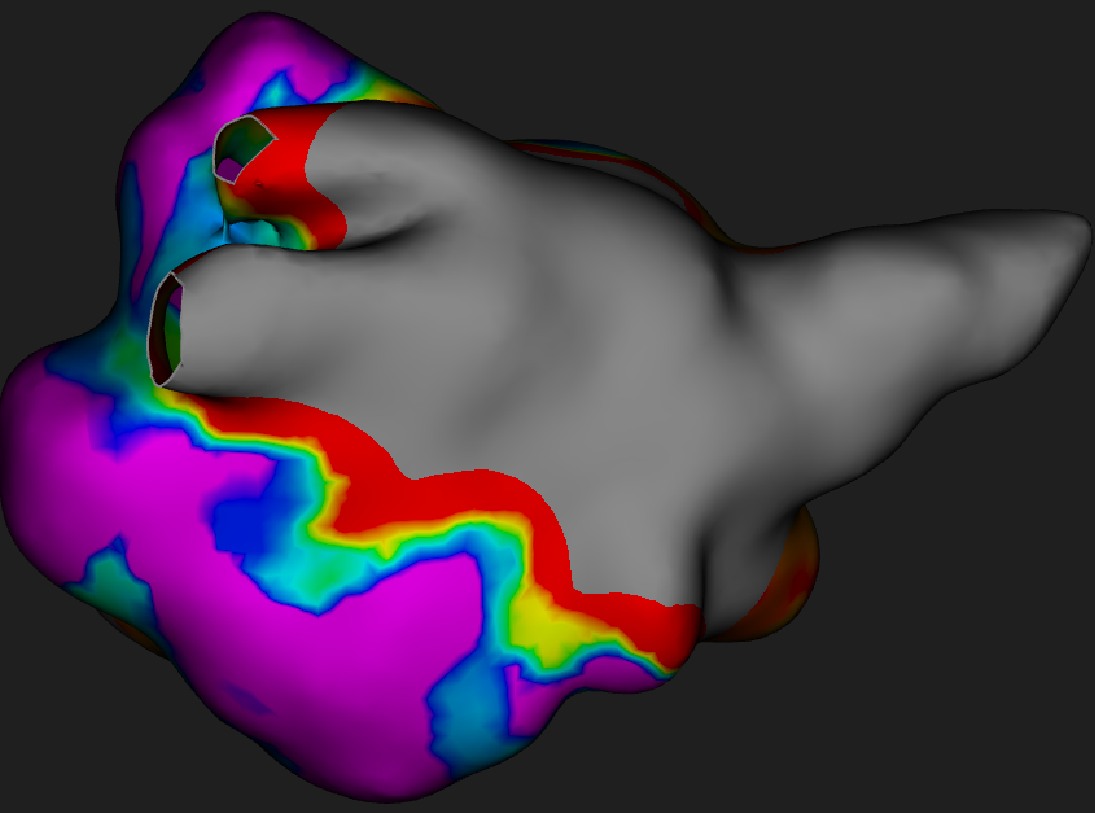 | 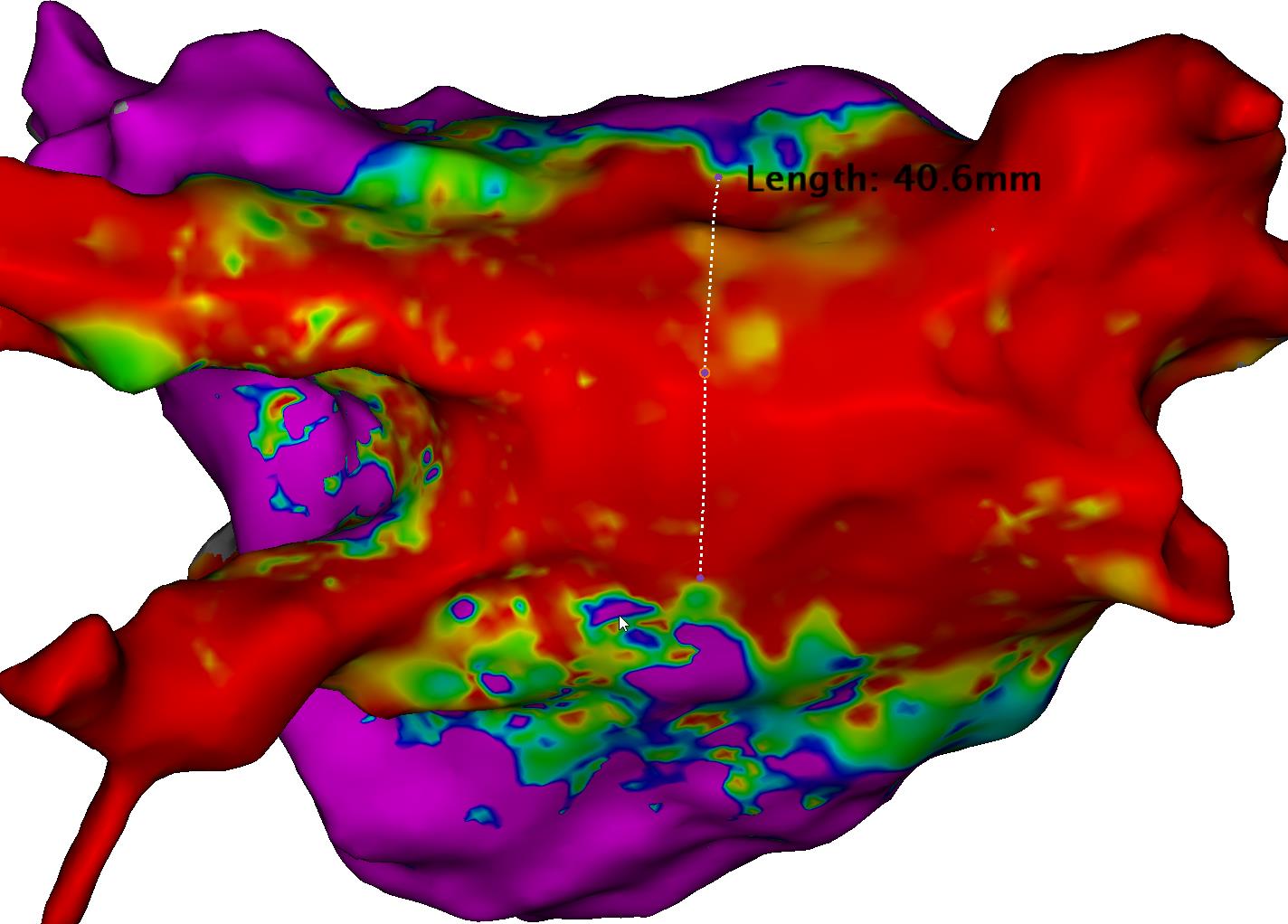 |
| 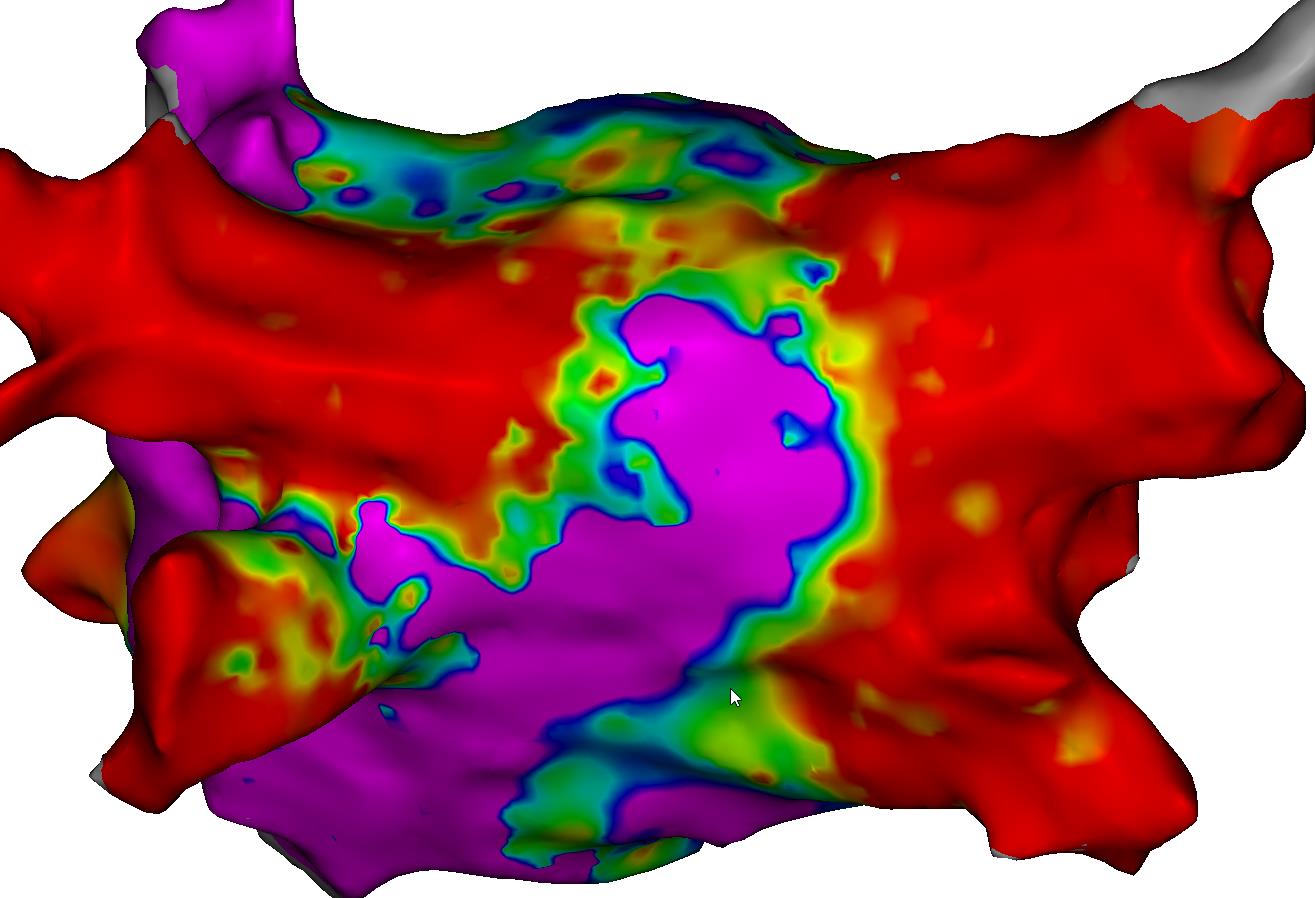 | 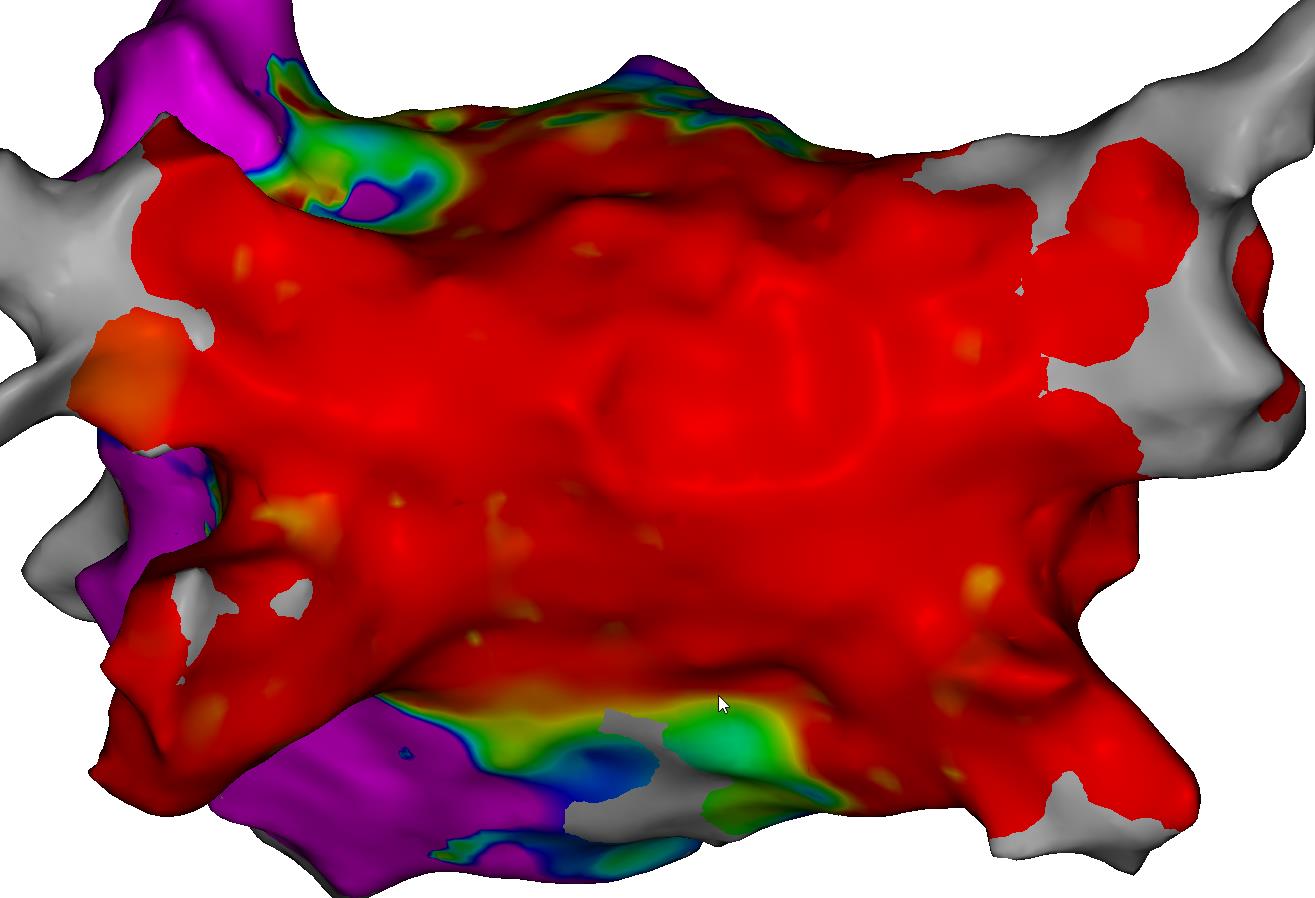 | 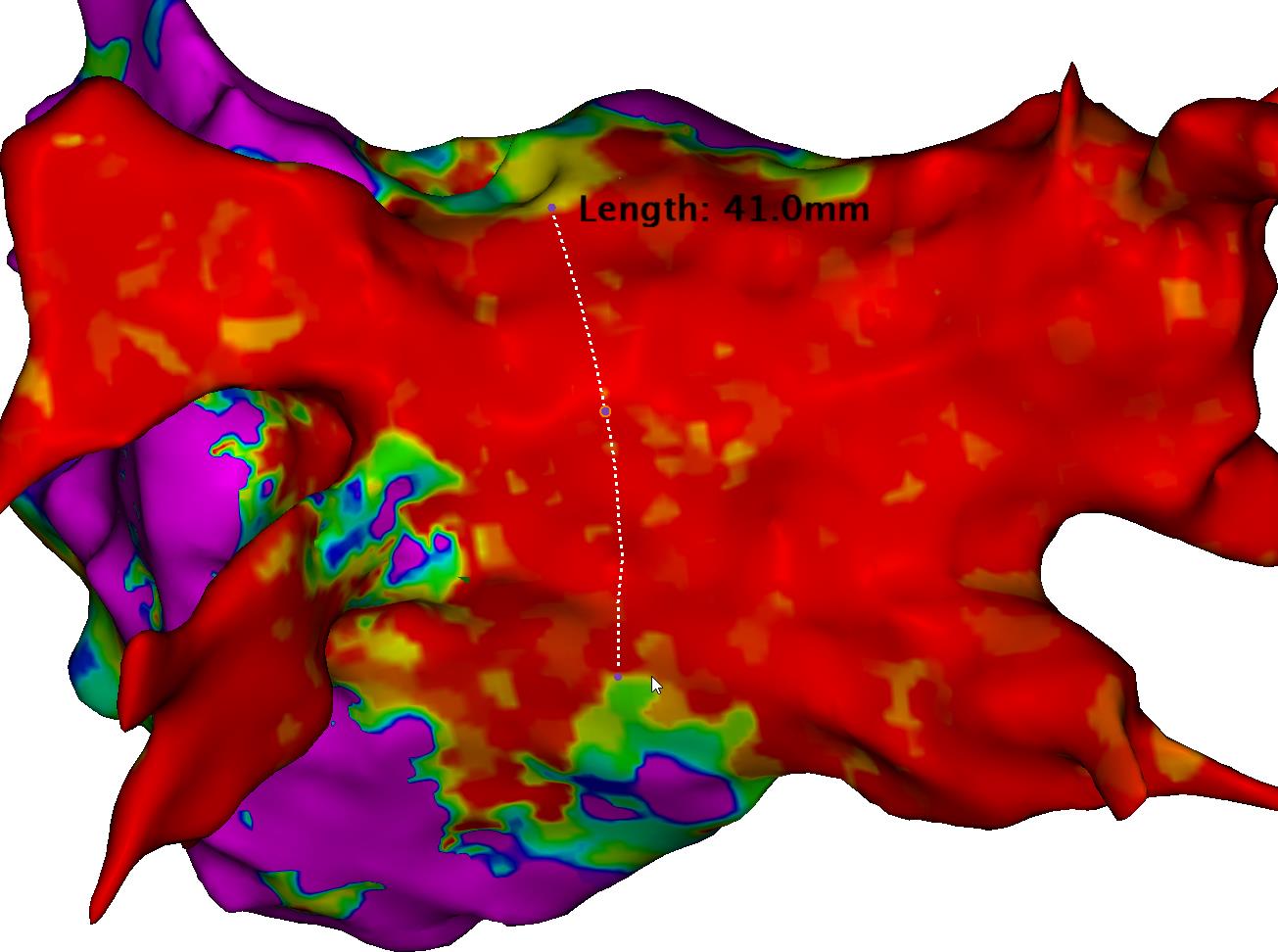 |
| 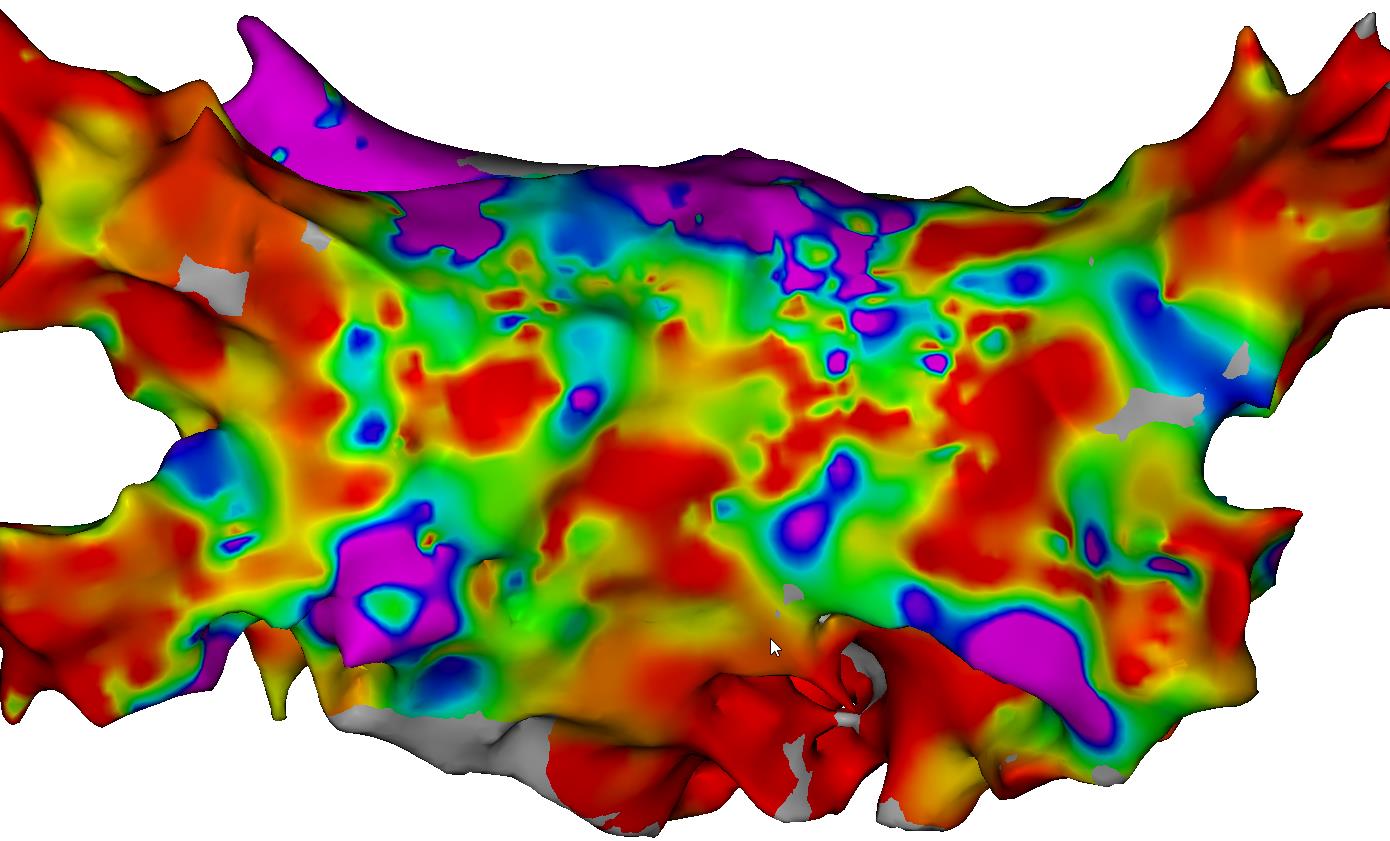 | 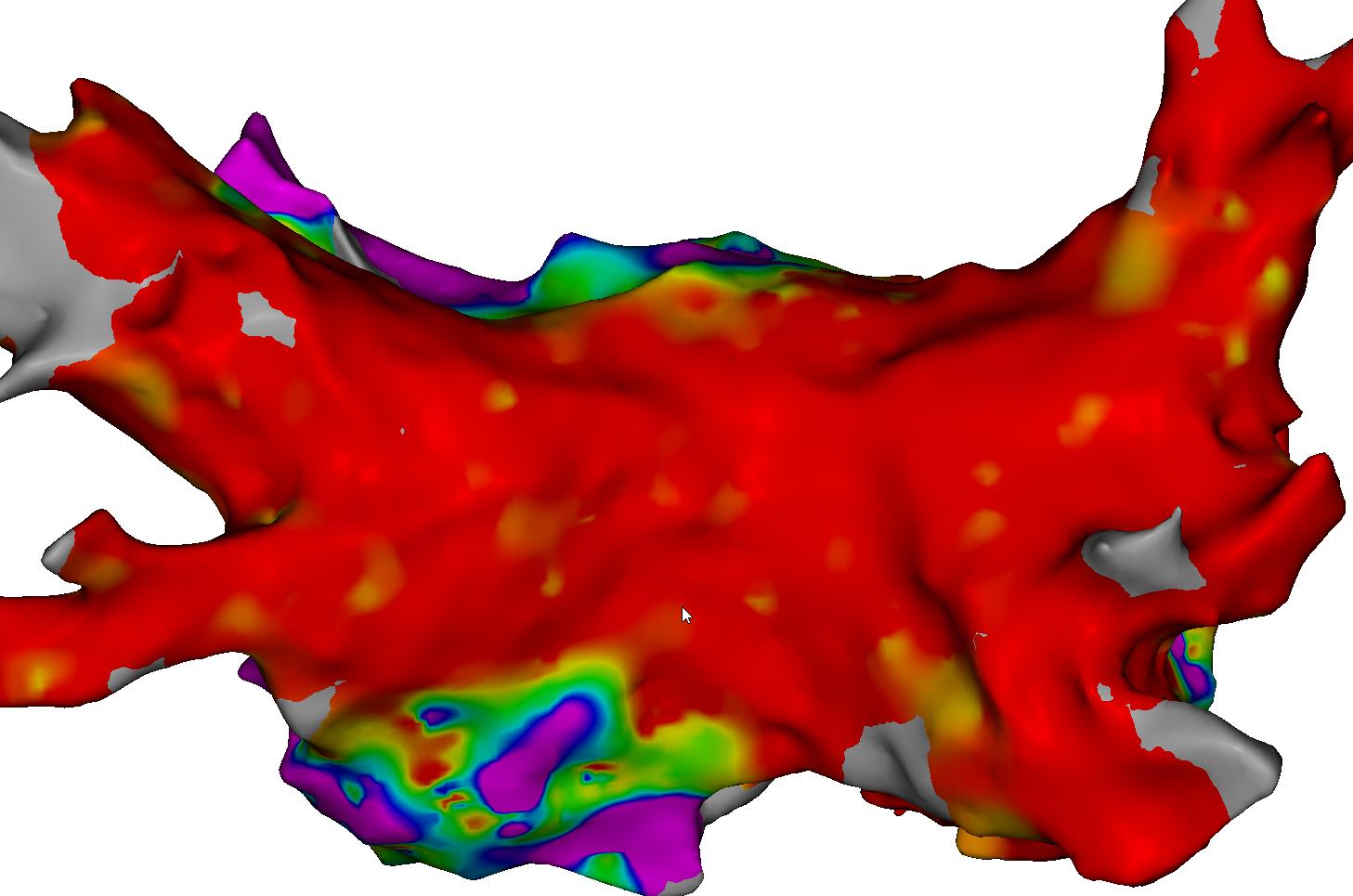 | 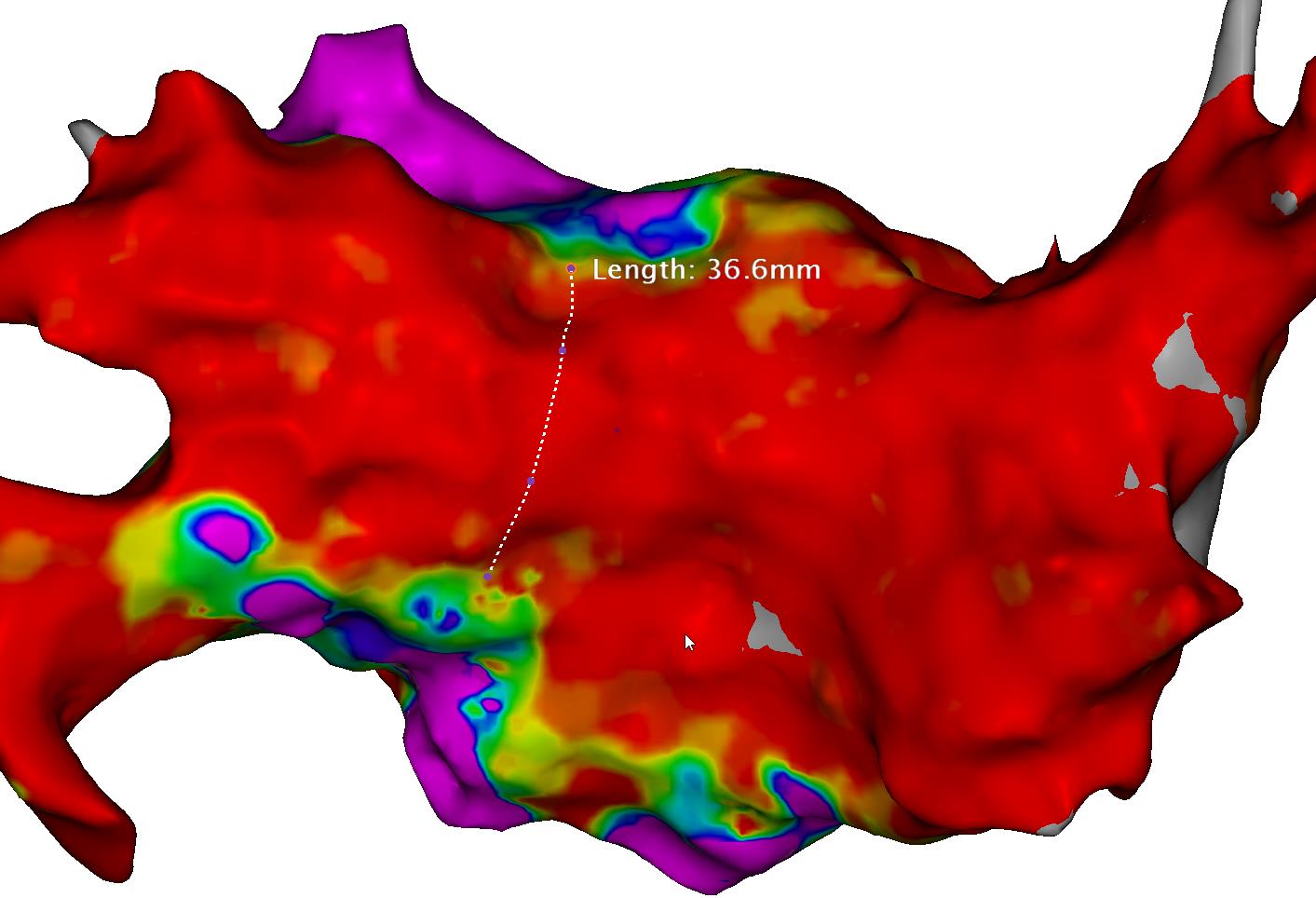 |

| **Supplementary Table. Procedural characteristics stratified by initial presenting arrhythmia** | | | | | |
| --- | --- | --- | --- | --- | --- |
| Initial presenting arrhythmia | Overall | Persistent | Paroxysmal | AT | p |
| n | 215 | 151 | 56 | 8 |  |
| Redo procedure | 145 (67.4) | 99 (65.6) | 44 (78.6) | 2 (25.0) | 0.007 |
| **Indication for PWA** |  |  |  |  | <0.001 |
| PW scar | 79 (36.7) | 63 (41.7) | 16 (28.6) | 0 |  |
| LA Flutter | 62 (28.8) | 31 (20.5) | 23 (41.1) | 8 (100.0) |  |
| No PW scar | 56 (26.0) | 47 (31.1) | 9 (16.1) | 0 |  |
| Anatomy | 18 (8.4) | 10 (6.6) | 8 (14.3) | 0 |  |
| **Previous procedure** |  |  |  |  |  |
| Previous procedures – Median [IQR] | 1 [0, 2] | 1.0 [0.0, 2.0] | 1.0 [1.0, 2.0] | 0.0 [0.0, 0.2] | 0.024 |
| Previous posterior MIL | 13 (9.0) | 6 (6.1) | 6 (13.6) | 1 (50.0) | 0.042 |
| Previous LA substrate ablation | 9 (6.2) | 3 (3.0) | 6 (13.3) | 0 | 0.055 |
| Previous CTI ablation | 43 (27.7) | 23 (22.1) | 15 (32.6) | 5 (100.0) | <0.001 |
| Previous SVC isolation | 15 (10.3) | 8 (8.1) | 7 (15.9) | 0 | 0.325 |
| **Current procedure** |  |  |  |  |  |
| Posterior MIL | 14 (6.5) | 7 (4.6) | 7 (12.5) | 0 | 0.094 |
| Technology used for post. MIL |  |  |  |  | 1.000 |
| PFA | 3 (21.4) | 1 (14.3) | 2 (28.6) | 0 |  |
| PFA and RFA | 8 (57.1) | 4 (57.1) | 4 (57.1) | 0 |  |
| RFA | 3 (21.4) | 2 (28.6) | 1 (14.3) | 0 |  |
| Anterior MIL | 28 (13.0) | 19 (12.6) | 7 (12.5) | 2 (25.0) | 0.591 |
| Technology used for ant. MIL |  |  |  |  | 0.636 |
| PFA | 20 (71.4) | 12 (63.2) | 6 (85.7) | 2 (100.0) |  |
| PFA and RFA | 5 (17.9) | 4 (21.1) | 1 (14.3) | 0 |  |
| RFA | 3 (10.7) | 3 (15.8) | 0 | 0 |  |
| LA substrate ablation | 20 (9.3) | 13 (8.6) | 7 (12.5) | 0 | 0.453 |
| CTI ablation using RFA | 37 (17.2) | 24 (15.9) | 13 (23.2) | 0 | 0.196 |
| SVC isolation using RFA | 7 (3.3) | 5 (3.3) | 2 (3.6) | 0 | 0.866 |
| **Next procedure** |  |  |  |  |  |
| LA redo procedure | 26 (12.1) | 17 (11.3) | 5 (8.9) | 4 (50.0) | 0.003 |
| Numbers are n (%) unless otherwise noted. | | | | | |
